# Global, regional, and national burden of 34 cancer groups across 204 countries and territories, 1990–2021, and projections to 2050: a systematic analysis of the Global Burden of Disease Study 2021

**DOI:** 10.3389/fonc.2025.1660125

**Published:** 2025-10-22

**Authors:** Linfeng Zhu, Jiangpeng Zhu, Qing Wang, Xiaotong Sun, YuTao Yuan, Sheng Ding, Jiafeng Zhao, Lei Liu, Shuangshuang Hou

**Affiliations:** ^1^ Department of General Surgery, Maanshan People’s Hospital, Maanshan, Anhui, China; ^2^ Department of Gastrointestinal Surgery, Wuhu Hospital, East China Normal University (The Second People’s Hospital, WuHu), Wuhu, Anhui, China; ^3^ Department of Central Laboratory, Taizhou Hospital of Zhejiang Province, Linhai, Zhejiang, China; ^4^ Department of General Surgery, Zibo Hospital of Integrated Traditional Chinese and Western Medicine, Zibo, Shandong, China; ^5^ Department of Gynecology, Obstetrics and Gynecology Hospital of Fudan University, Shanghai, China; ^6^ Department of General Surgery, Fuyang Normal University Second Affiliated Hospital, Fuyang, Anhui, China

**Keywords:** cancer, incidence, mortality, sociodemographic index, Global Burden of Disease

## Abstract

**Introduction:**

Detailed, comprehensive, and timely reporting on cancer incidence, mortality, and associated burdens is essential for understanding the evolving trends of this complex disease. This study aims to evaluate the burdens of 34 types of cancer across 204 countries and territories from 1990 to 2021, incorporating the latest epidemiological data to provide a framework for policy discussions, resource allocation, and research prioritization.

**Methods:**

Using data from the Global Burden of Disease (GBD) 2021 study, we analyzed 34 cancer types across 204 countries from 1990 to 2021, stratifying by age, sex, region, and sociodemographic index (SDI) and projecting future trends using a Bayesian age-period-cohort model.

**Results:**

In 2021, there were 23.57 million new cases and 9.83 million deaths, with a global age-standardized incidence rate (ASIR) of 275.2 per 100,000 and age-standardized mortality rate (ASMR) of 115.8 per 100,000. Cancer incidence has risen 2.3-fold, with the ASIR increasing by 8.8% and the ASMR decreasing by 21.5% over the period since 1990. Tracheal, bronchial, and lung cancer, colorectal cancer, stomach cancer, and breast cancer were the leading causes of mortality, while nonmelanoma skin cancer, tracheal, bronchial, and lung cancer, colorectal cancer, breast cancer, and prostate cancer had the highest incidence rates. Men had 1.2 times higher incidence and 1.3 times higher mortality than women. North America had the highest ASIR, while East Africa had the highest ASMR. High-SDI countries had higher incidence, whereas low-SDI countries bore the highest mortality burden. By 2050, cancer incidence is projected to exceed 26 million cases, with prevalence surpassing 100 million, while mortality is expected to decline by 14.8%.

**Conclusions:**

Despite advances in detection and treatment, the rising global cancer burden and persistent disparities highlight the urgent need for equitable prevention, early diagnosis, and resource allocation to reduce cancer mortality worldwide. The potential long-term impact of COVID-19 on cancer development and progression remains unclear, warranting further investigation in the post-pandemic era.

## 1 Introduction

Noncommunicable diseases (NCDs), particularly cancer, cardiovascular diseases, chronic respiratory diseases, and diabetes, are among the leading causes of death globally, accounting for approximately 74% of all deaths worldwide (1). Cancer is the second leading cause of death, with an estimated global mortality of over 9.3 million people annually due to noncommunicable diseases. Cancer is a major contributor to the global disease burden, and it is projected that the global cancer burden will continue to rise for at least the next 20 years (2–4). The United Nations Sustainable Development Goals highlight the importance of addressing the cancer burden under Target 3.4. This target aims to reduce premature mortality from noncommunicable diseases (NCDs), including cancer, by one-third by 2030 through prevention and treatment (5, 6). Additionally, it emphasizes the promotion of mental health and well-being as part of a broader strategy to combat NCDs. Despite growing awareness of the threat NCDs pose to human development, progress in controlling NCDs remains slow in most countries (7). Unlike other health threats, such as tuberculosis, human immunodeficiency virus, or malaria, cancer encompasses many distinct diseases requiring unique approaches for prevention, diagnosis, and treatment. Few countries have successfully addressed this challenge.

Monitoring the severity and demographic, spatial, and temporal variations of cancer burden is crucial for adjusting health planning and setting priorities for clinical care and research (8). Policy-makers need localized data to evaluate cancer control programs, establish progress benchmarks, and allocate resources effectively, although some countries lack cancer surveillance systems. Given that cancer is recognized as a national health priority by the World Health Organization and many governments, detailed epidemiological data on different cancer types are particularly important in lower- and middle-income countries (LMICs) and low-income countries (LICs), where health priorities often compete.

The Global Burden of Diseases, Injuries, and Risk Factors Study 2021 (GBD 2021) framework facilitates the comparative evaluation of cancer burdens across different regions and time periods by examining cancer incidence, mortality, and disability-adjusted life years (DALYs). The aim of this study was to compare the incidence and mortality of 34 cancers across 204 countries or regions from 1990 to 2021, providing guidance for global cancer prevention and control. The presented results offer up-to-date information to better understand the evolution and trends of cancer burden across countries with different levels of development.

## 2 Methods

Data on annual incidence, mortality, and age-standardized rates for these 34 cancer types were collected from the Global Burden of Diseases, Injuries, and Risk Factors Study (GBD) 2021, covering various demographic categories, including sex, region, country, and age. The dataset comprehensively evaluates the impact of 371 diseases and 88 risk factors across 204 countries and 811 subnational locations (9, 10). The Global Health Data Exchange (GHDx) query tool, an online health data resource provided by the Institute for Health Metrics and Evaluation (IHME), is used to collect annual frequencies and age-standardized rates (ASRs) of cancer-related incidence, mortality, prevalence, and disability-adjusted life years (DALYs) for 34 types of cancer. All statistical analyses and data visualizations were performed using R (version 4.4.2) and JD_GBDR (V2.37, Jingding Medical Technology Co., Ltd.). In this study, the R software package (version4.2.3) and JD_GBDR (V2.22, Jingding Medical Technology Co., Ltd.) was used for the drawing of the figures. The detailed methodology is provided in the **Supplementary Materials**.

## 3 Results

### 3.1 The global burden of cancer in 2021

**Figure 1:**
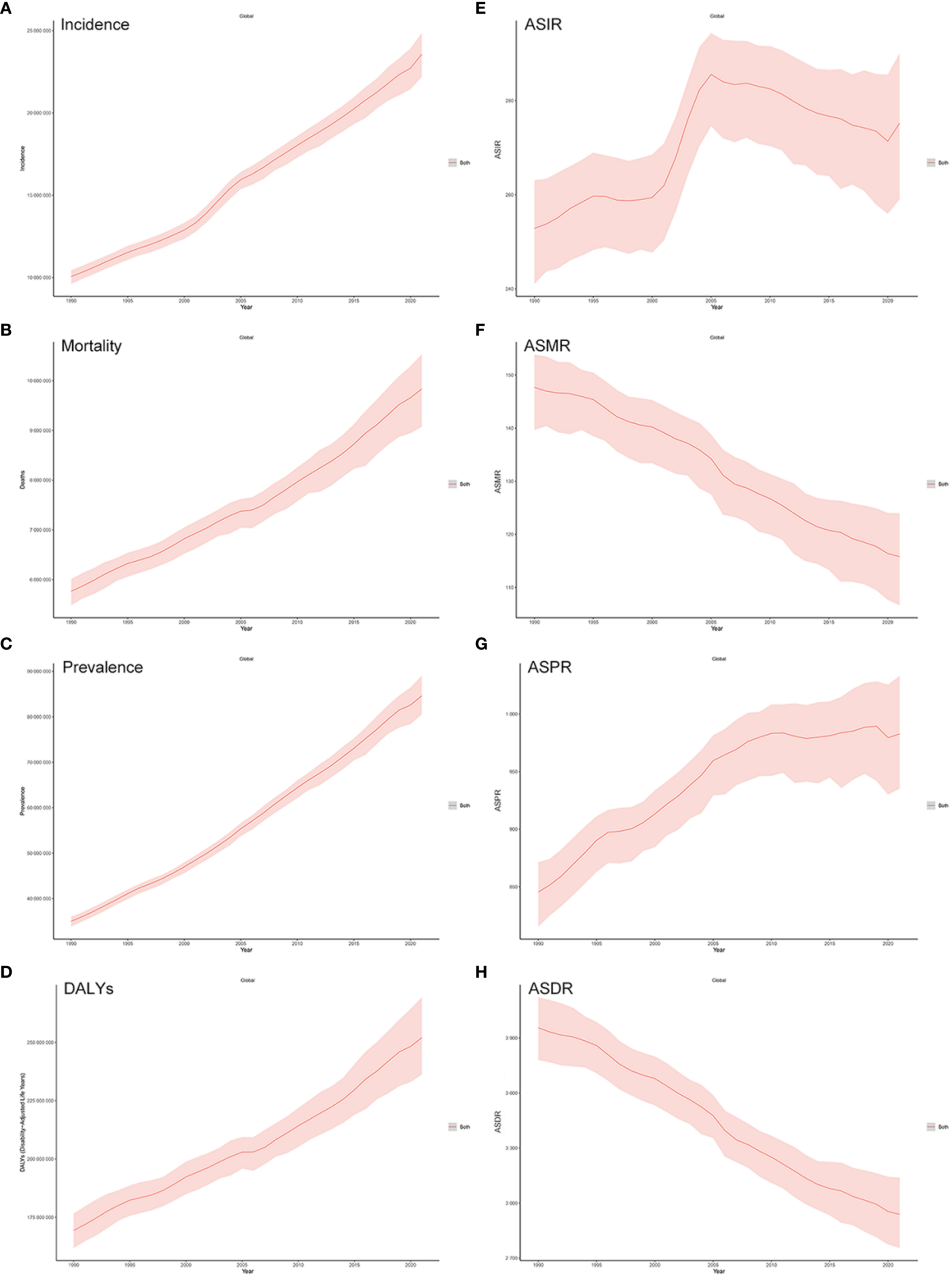
**(A–D)** Trends in global cancer incidence, mortality, prevalence, and disability-adjusted life years (DALYs) from 1990 to 2021. **(E–H)** Trends in global cancer ASIR, ASMR, ASPR, and ASDR from 1990 to 2021.

**Table 1** presents the incidence, age-standardized incidence rates (ASIRs), deaths, and age-standardized mortality rates (ASMRs) for 34 cancer types from 1990 to 2021. In 2021, an estimated 23.6 million (95% UI: 22.2–24.8 million) new cancer cases were reported globally, with an ASIR of 275.2 (95% UI: 259.0–290.0) per 100,000 population. Following the outbreak of COVID-19 at the end of 2019, from 2005 to 2019, the annual growth rate of cancer incidence was 2.43% (2.12%–2.83%), compared to 2.74% (1.73%–3.75%) during 2019–2021, indicating that observed trends post-COVID-19 onset appear broadly consistent with pre-pandemic patterns, although caution is warranted due to potential disruptions in cancer detection, diagnosis, and registry reporting. Globally, 9.83 million (95% UI: 9.07–10.53 million) cancer deaths occurred in 2021, with an ASMR of 115.8 (95% UI: 106.7–124.0). Mortality growth rates were similar before and after the pandemic: 1.84% (0.32%–2.50%) in 2005–2019 *vs*. 1.63% (1.42%–1.84%) in 2019–2021. Compared with 1990, the number of new cancer cases in 2021 increased by 2.3 times, accompanied by an 8.8% increase in the ASIR. Similarly, the total number of cancer-related deaths increased by 1.7 times, whereas the ASMR decreased by 21.5% (**Figure 1**). Cancers with the highest ASMRs included tracheal, bronchus, and lung (TBL) cancer, colon and rectum, stomach, breast, esophageal, pancreatic, liver, and prostate cancers—together accounting for 67.2% of all cancer deaths in 2021. Most cancers showed declining ASMRs since 1990, with stomach cancer decreasing nearly 50% (from 22.0 to 11.2). However, ASMRs slightly increased for pancreatic and nonmelanoma skin cancers. From 1990 to 2021, ASIRs rose for most cancers—most notably nonmelanoma skin (64.5%), breast (14.9%), prostate (12.3%), and colon and rectum cancer (6.5%)—but declined for stomach (−42.1%), esophageal (−22.9%), and TBL cancers (−7.4%). Assessment of the burden associated with major cancers (TBL cancer, colon and rectum cancer, breast cancer, stomach cancer, liver cancer, pancreatic cancer, prostate cancer, and thyroid cancer) is shown in the **Supplementary Results** in **Supplementary Material**.

**Table 1:** Global incidence and deaths in 1990 and 2021 for total cancers and 34 cancer groups.

Cancer type	1990												2021											
	Deaths						Incidence						Deaths						Incidence					
	Number (95% UI)			ASMR (95% UI)			Number (95% UI)			ASIR (95% UI)			Number (95% UI)			ASMR (95% UI)			Number (95% UI)			ASIR (95% UI)		
	Value	Upper	Lower	Value	Upper	Lower	Value	Upper	Lower	Value	Upper	Lower	Value	Upper	Lower	Value	Upper	Lower	Value	Upper	Lower	Value	Upper	Lower
Total cancer	5765726.9	6006325.6	5484721.8	147.7	153.9	139.7	10059627.7	10437810.2	9617493.9	252.8	263.1	241.1	9833234.4	10526278.6	9074786.5	115.8	124.0	106.7	23566245.4	24847937.2	22200038.2	275.2	290.0	259.0
Total cancer without NMSC	5743059.9	5982616.3	5463233.4	147.0	153.2	139.0	8397983.7	8677080.5	8065662.8	207.8	214.8	198.3	17229399.3	18333270.2	16081547.6	201.1	213.7	187.4	9776321.2	10467969.8	9021866.4	115.1	123.2	106.0
Bladder cancer	123125.4	129806.8	113327.4	3.5	3.7	3.2	260141.8	272206.6	242823.3	6.9	7.2	6.5	221888.3	242326.5	200567.2	2.7	2.9	2.4	540309.7	582579.4	494720.9	6.3	6.9	5.8
Brain and central nervous system cancer	136218.9	155197.4	114799.3	3.0	3.5	2.6	173086.4	194951.2	147452.5	3.7	4.2	3.2	258626.8	296133.9	222185.2	3.1	3.5	2.6	357482.3	407432.6	310456.7	4.3	4.9	3.7
Breast cancer	355476.5	373476.1	335469.8	9.2	9.6	8.6	875657.2	910528.9	834228.9	21.4	22.2	20.3	674199.4	720822.6	623371.5	7.9	8.4	7.3	2121564.3	2268722.6	1982142.6	24.6	26.3	22.9
Cervical cancer	211483.8	229841.0	195724.3	5.1	5.5	4.7	409548.5	438505.6	383207.2	9.3	9.9	8.7	296667.2	321905.7	272058.6	3.4	3.7	3.2	667426.4	726422.1	613030.1	7.8	8.5	7.2
Colon and rectum cancer	570318.5	597668.7	536544.8	15.6	16.3	14.5	916583.5	951895.0	866238.3	24.0	25.0	22.5	1044072.2	1120169.3	950187.6	12.4	13.3	11.2	2194143.3	2359390.1	2001271.8	25.6	27.5	23.3
Esophageal cancer	356263.4	390154.3	319362.8	9.0	9.9	8.1	354730.8	388914.5	317512.4	8.9	9.7	8.0	538601.9	603405.5	475944.0	6.3	7.0	5.5	576529.3	645648.5	509492.1	6.7	7.4	5.9
Eye cancer	7518.7	8631.0	5756.3	0.2	0.2	0.1	18002.2	21233.1	14772.1	0.4	0.5	0.3	10432.8	12230.9	8013.5	0.1	0.2	0.1	33950.8	39857.3	26532.9	0.4	0.5	0.3
Gallbladder and biliary tract cancer	98683.1	109189.4	88940.5	2.7	3.0	2.4	107797.8	117511.8	96890.2	2.9	3.1	2.6	171961.2	194238.4	142351.8	2.0	2.3	1.7	216768.3	245237.6	181888.0	2.6	2.9	2.2
Hodgkin lymphoma	28977.1	33106.9	21389.7	0.6	0.7	0.5	54671.4	59832.2	45648.6	1.1	1.2	0.9	28180.0	35653.0	20895.4	0.3	0.4	0.3	65182.0	77143.0	53167.4	0.8	0.9	0.6
Kidney cancer	77420.7	79691.3	74807.4	2.0	2.1	1.9	159774.3	163926.3	154831.2	3.9	4.0	3.8	161194.5	169348.3	150317.6	1.9	2.0	1.8	387828.7	406635.2	365359.7	4.5	4.7	4.3
Larynx cancer	85789.7	91207.6	80408.8	2.1	2.3	2.0	125175.1	131639.0	118981.1	3.1	3.2	2.9	117251.6	125952.4	109354.6	1.3	1.4	1.3	200883.0	216097.6	186941.1	2.3	2.5	2.1
Leukemia	248105.2	277354.5	217786.3	5.6	6.1	5.0	311647.6	343177.4	278619.9	6.9	7.5	6.2	320283.6	349049.9	274969.5	3.9	4.2	3.3	461422.7	504397.0	397547.9	5.6	6.2	4.8
Lip and oral cavity cancer	97402.3	102361.0	92506.4	2.5	2.6	2.3	174077.5	181621.6	167404.2	4.3	4.5	4.1	208379.4	224162.1	191288.0	2.4	2.6	2.2	421577.1	449782.1	389878.8	4.9	5.2	4.5
Liver cancer	238969.1	263037.0	218717.0	5.9	6.5	5.4	244689.4	268549.0	224795.5	5.9	6.5	5.4	483875.1	540177.1	440400.3	5.6	6.3	5.1	529202.5	593849.1	480339.5	6.2	6.9	5.6
Malignant neoplasm of bone and articular cartilage	35552.2	42011.1	32334.3	0.8	0.9	0.7	46583.0	54215.4	42150.5	1.0	1.1	0.9	66114.3	74466.9	53305.4	0.8	0.9	0.6	91375.1	102469.7	73780.4	1.1	1.3	0.9
Malignant skin melanoma	33061.0	34529.5	30546.4	0.8	0.9	0.8	124319.8	127610.5	119603.9	3.0	3.1	2.9	61549.7	66265.0	54852.4	0.7	0.8	0.7	303104.6	318904.8	281717.6	3.6	3.7	3.3
Mesothelioma	15083.8	16241.4	14040.8	0.4	0.4	0.4	16493.2	17783.4	15324.9	0.4	0.5	0.4	29618.9	31764.6	27424.5	0.3	0.4	0.3	31907.9	34115.3	29643.1	0.4	0.4	0.3
Multiple myeloma	47569.0	51416.5	44137.5	1.3	1.4	1.2	55710.1	59687.8	52022.5	1.5	1.6	1.4	116359.6	128470.6	103078.6	1.4	1.5	1.2	148754.6	162049.2	131780.4	1.7	1.9	1.5
Nasopharynx cancer	64932.9	71096.2	58544.6	1.5	1.7	1.4	76255.8	83570.2	68714.0	1.7	1.9	1.6	75358.7	83706.3	67515.4	0.9	1.0	0.8	118877.9	135884.4	104836.1	1.4	1.6	1.2
Neuroblastoma and other peripheral nervous cell tumors	2674.8	3138.4	2298.2	0.1	0.1	0.0	5854.2	7643.1	4516.8	0.1	0.1	0.1	5193.8	5932.3	4294.6	0.1	0.1	0.1	10867.4	13557.0	8279.0	0.1	0.2	0.1
Non-Hodgkin lymphoma	146657.4	160542.1	136931.0	3.6	3.9	3.4	255667.8	272800.9	242749.3	6.1	6.5	5.8	267061.2	288695.7	246094.7	3.2	3.4	2.9	604554.1	648746.2	558229.2	7.1	7.7	6.6
Non-melanoma skin cancer	22667.0	24674.9	20674.1	0.7	0.7	0.6	1661644.0	1901240.3	1430711.6	45.0	51.0	39.4	56913.2	63037.4	48761.4	0.7	0.8	0.6	6336846.1	6896046.6	5744729.4	74.1	80.7	67.3
Other malignant neoplasms	142713.7	155425.7	126682.7	3.5	3.8	3.1	205977.2	222043.3	184968.5	4.9	5.2	4.4	222208.4	240746.7	199406.3	2.7	2.9	2.4	422295.0	462634.9	380537.2	5.0	5.5	4.5
Other neoplasms	18690.1	23975.7	16001.9	0.6	0.7	0.5	24715051.7	30340404.7	19991541.9	505.4	613.1	405.9	55179.1	64295.5	46761.0	0.7	0.8	0.6	42913361.9	51701423.4	34917159.4	515.2	620.5	420.9
Other pharynx cancer	44343.7	48189.9	40993.7	1.1	1.2	1.0	64528.9	68919.7	60828.8	1.6	1.7	1.5	98435.1	105484.9	91566.8	1.1	1.2	1.0	169819.5	179704.2	159847.1	1.9	2.0	1.8
Ovarian cancer	100584.1	109087.0	92970.8	2.6	2.8	2.4	159096.0	174055.0	145708.9	3.8	4.2	3.5	185608.7	201012.7	167962.0	2.2	2.3	1.9	298876.0	324501.0	270729.8	3.5	3.8	3.1
Pancreatic cancer	211612.7	221951.0	199990.3	5.7	5.9	5.3	207905.2	217778.5	196649.4	5.5	5.7	5.2	505752.2	543899.4	461224.4	5.9	6.4	5.4	508532.7	547207.6	462090.9	6.0	6.4	5.4
Prostate cancer	211971.5	224327.7	194221.9	6.3	6.7	5.8	506405.2	524697.4	480851.4	13.7	14.2	13.0	432463.3	463645.3	381872.8	5.3	5.6	4.6	1324382.9	1400222.2	1217320.9	15.4	16.3	14.1
Soft tissue and other extraosseous sarcomas	31878.3	37708.1	26445.8	0.7	0.9	0.6	54630.9	63999.6	46757.2	1.2	1.4	1.0	50203.1	61280.4	43232.0	0.6	0.7	0.5	96201.0	116184.9	83423.5	1.2	1.4	1.0
Stomach cancer	854184.5	939972.7	772885.1	22.0	24.2	20.0	980899.4	1072236.0	891306.8	24.8	27.0	22.6	954373.6	1089576.6	821750.8	11.2	12.7	9.6	1230232.6	1409969.7	1052350.1	14.3	16.4	12.2
Testicular cancer	7618.4	8075.6	7191.6	0.2	0.2	0.2	38833.5	40260.8	37572.5	0.7	0.8	0.7	11388.3	12055.6	10770.7	0.1	0.1	0.1	91507.4	95709.7	87965.9	1.1	1.2	1.1
Thyroid cancer	21893.0	24108.1	20437.5	0.6	0.6	0.5	89885.5	96998.8	84681.3	2.1	2.2	2.0	44798.5	48541.0	39924.7	0.5	0.6	0.5	249538.0	274638.2	223290.3	2.9	3.2	2.6
Tracheal, bronchus, and lung cancer	1080127.6	1135557.1	1023326.9	27.6	29.0	26.1	1132063.6	1186163.4	1075370.9	28.5	29.9	27.1	2016547.4	2218371.9	1820497.7	23.5	25.8	21.2	2280688.2	2509739.7	2063251.9	26.4	29.1	23.9
Uterine cancer	54848.9	59116.1	48803.6	1.4	1.6	1.3	191290.8	201941.4	175002.6	4.7	5.0	4.3	97672.1	108061.5	86515.8	1.1	1.3	1.0	473613.9	513666.9	429915.6	5.4	5.9	4.9

NMSC, Non-melanoma skin cancer.

### 3.2 Impact of sex and age on the cancer burden

**Figure 2:**
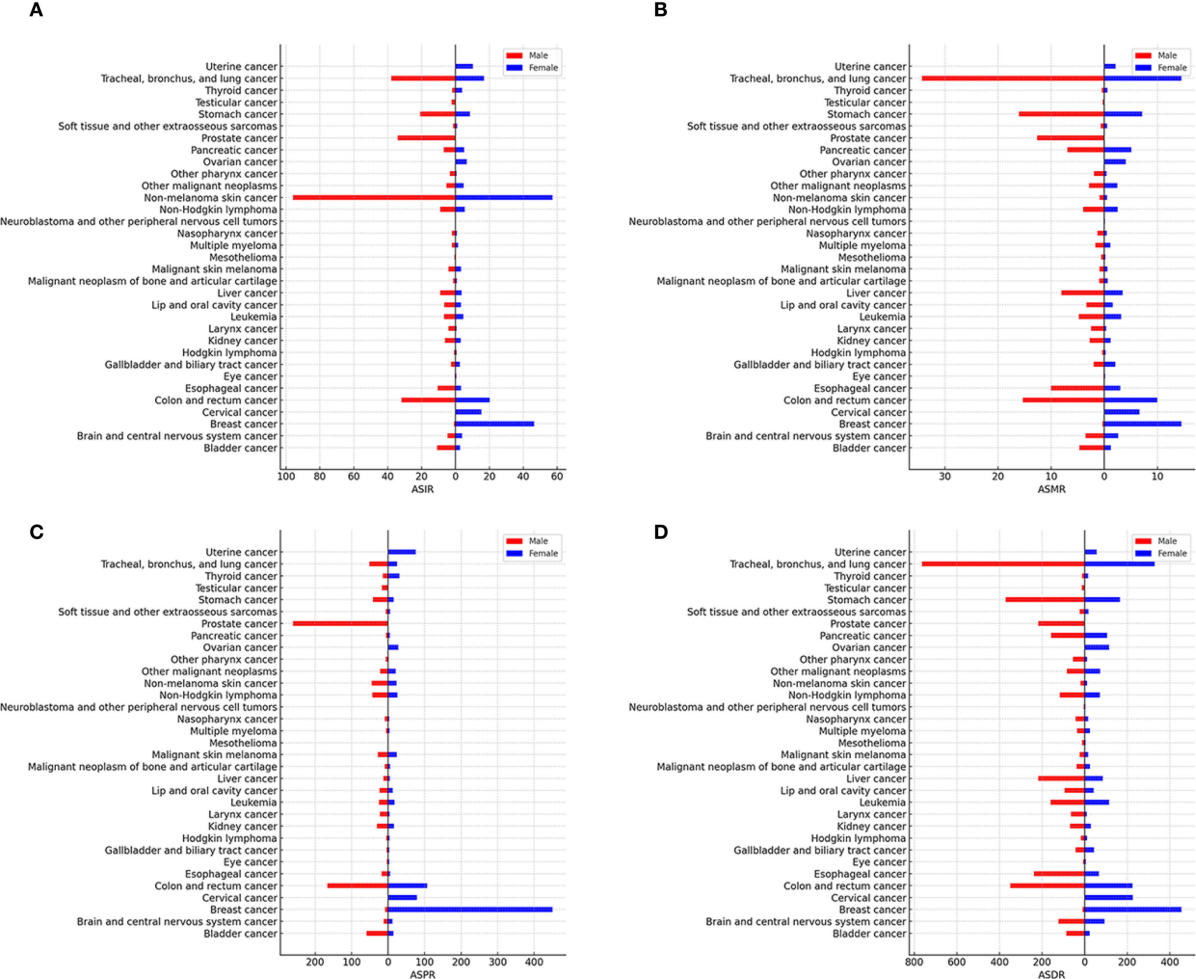
Age-standardised global cancer incidence **(A)**, mortality **(B)**, prevalence **(C)**, and disability-adjusted life years (DALYs) **(D)** for 34 specific cancer types analysed for 2021 and stratified by sex.

In 2021, cancer incidence and mortality were markedly higher in men than women, with rates approximately 1.2 and 1.3 times greater, respectively (**Supplementary Table S1**, **Supplementary Figure S1**). Total cancer deaths numbered 5.57 million in males and 4.26 million in females. Among men, tracheal, bronchus, and lung (TBL) cancer had the highest ASMR (34.3), accounting for 1.34 million deaths (24.1% of male cancer deaths), followed by stomach, colon and rectum, prostate, and esophageal cancers. (**Figure 2**, **Supplementary Table S1**). Additionally, these cancers presented significantly elevated ASIRs in males (**Figure 2**, **Supplementary Table S1**). For women, breast cancer ranked first in ASMR (14.5), causing 660,925 deaths (15.5% of female cancer deaths), followed by TBL, colon and rectum, stomach, and cervical cancers. Breast cancer also led in disability-adjusted life years (DALYs) and prevalence, highlighting its significant burden (**Figure 2**).

Age-specific analyses (**Supplementary Figures S2, S3** **, Supplementary Tables S3, S4**) showed distinct patterns. Incident case distribution was 5.0% (0–14 years), 35.6% (15–49), 36.6% (50–69), and 22.8% (≥70). Mortality followed a different distribution: 0.8%, 10.0%, 39.6%, and 49.6% across the same age groups. In children aged 0–14 years, leukemia was the leading cause of both incidence and mortality, followed by brain and central nervous system tumors. Among individuals aged 15–49 years, breast, nonmelanoma skin, cervical, colon and rectum, and stomach cancers were most common, with breast cancer, TBL cancer, cervical cancer, colon and rectum cancer, and stomach cancer being the top causes of death. In the 50–69 year group, TBL (860,322 deaths), stomach (380,834), colon and rectum (373,101), breast (297,805), and esophageal cancer (245,400) were major contributors to mortality. For those aged ≥70, TBL, colon and rectum, and stomach cancers accounted for the majority of deaths. Cancer prevalence was highest among individuals aged 50–69 (39.7%), followed by 15–49 (32.3%), ≥70 (24.5%), and 0–14 (3.5%). TBL cancer caused the greatest number of DALYs, comprising 89.9% of the burden in those aged ≥50. Overall, the distribution of cancer incidence and mortality varied significantly by age.

### 3.3 Geographic differences in the cancer burden

**Figure 3:**
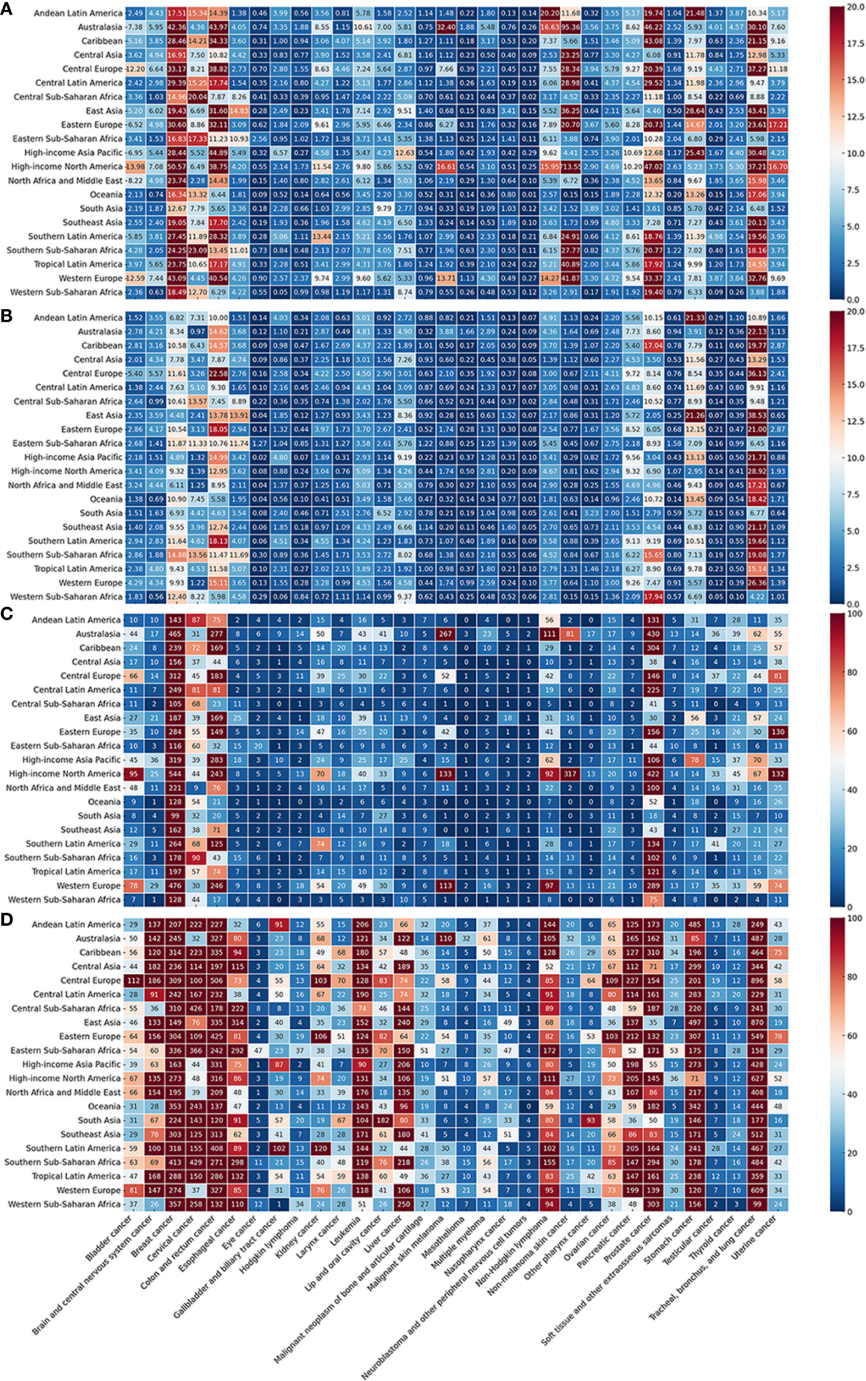
Age-standardised global cancer incidence **(A)**, mortality **(B)**, prevalence **(C)**, and DALYs **(D)** for 34 specific cancer types across 21 global regions in 2021.

The ASMRs, ASIRs, ASDRs, and ASPRs of the 34 cancer types in 21 regions in 2021 are shown in **Figure 3**. Global rankings of cancer types by incidence, prevalence, mortality, and DALY loss are detailed in **Supplementary Figures S4–S7**. In 2021, the highest number of incident cases occurred in High-income North America (6.76 million), East Asia (5.49 million), and Western Europe (2.97 million), accounting for 28.7%, 23.2%, and 12.6% of global incidence, respectively. Cancer deaths were most numerous in East Asia (2.91 million), Western Europe (1.23 million), and South Asia (1.11 million), comprising 29.4%, 12.5%, and 11.2% of total global deaths. High-income North America had the highest ASIR (3,138.7 per 100,000), followed by Central Europe (1,308.5) and High-income Asia Pacific (1,253.3). In contrast, Eastern, Western, and Central Sub-Saharan Africa had the lowest ASIRs. The highest ASMRs were observed in Southern Sub-Saharan Africa (144.2), East Asia (137.1), and Southern Latin America (129.2), while South Asia reported the lowest. High-income North America, High-income Asia Pacific, and Central Europe recorded the highest ASPRs. Central Europe had the highest ASDR, while South Asia had the lowest, reflecting regional differences in cancer-related health loss. By cancer type, nonmelanoma skin cancer had the highest ASIR globally, especially in High-income North America. TBL cancer had the highest ASMR in multiple regions, including East Asia, Central Europe, and Western Europe. Breast cancer showed the highest ASPR, particularly in High-income North America, Western Europe, and Australasia. TBL cancer also contributed the most to DALYs, leading ASDRs in Central Europe, East Asia, Western Europe, and several other regions (**Figure 3**).

### 3.4 Cancer burden by socioeconomic development level

**Figure 4:**
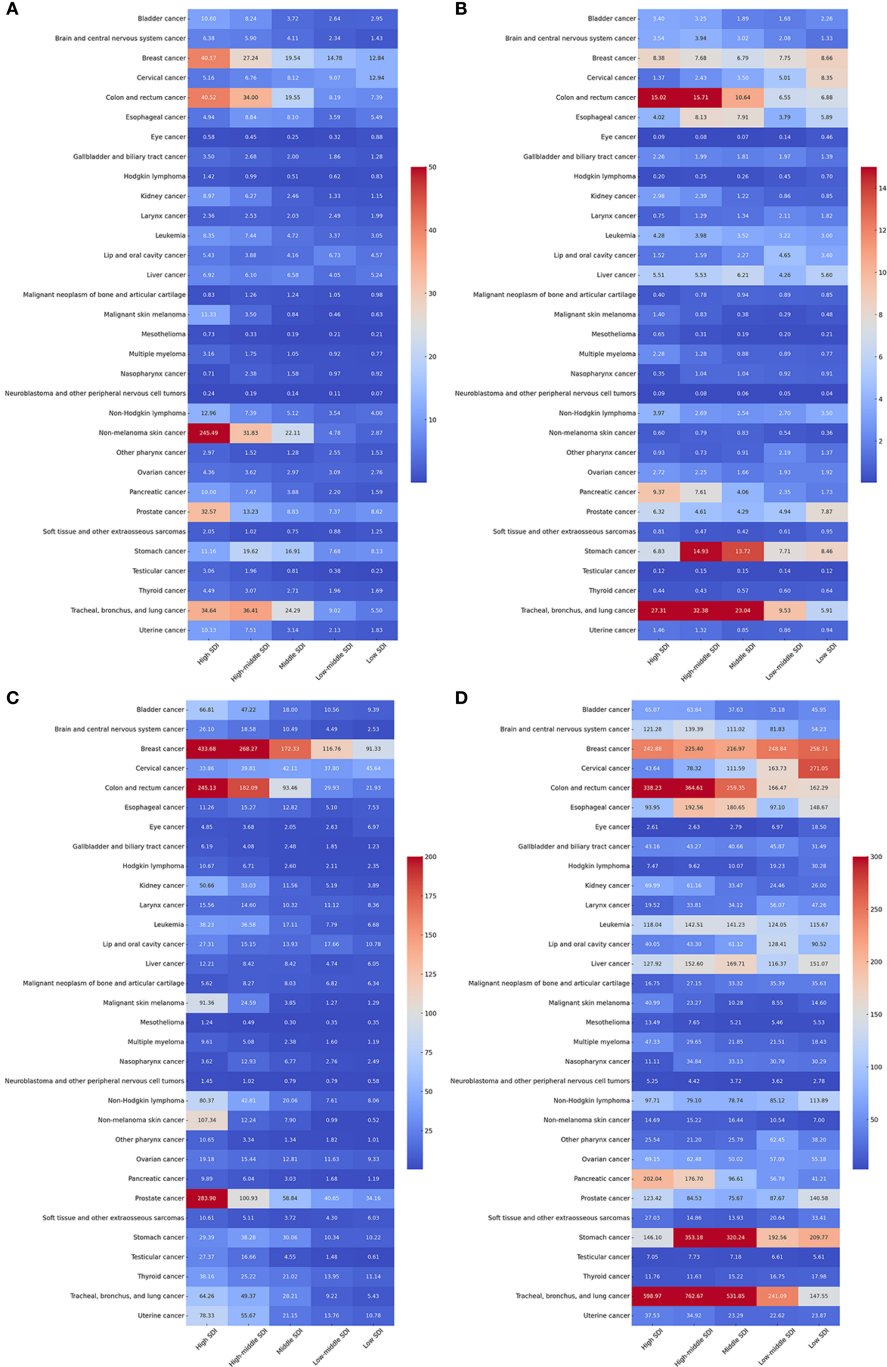
Age-standardised global cancer incidence **(A)**, mortality **(B)**, prevalence **(C)**, and DALYs **(D)** for 34 specific cancer types by sociodemographic index status in 2021.

The global burden of 34 cancer groups varied substantially across the five levels of the SDI, as detailed in **Figure 4**. Trends in global cancer incidence, mortality, prevalence, and DALYs, along with age-standardized incidence, mortality, prevalence, and DALYs from 1990 to 2021, by SDI, are shown in **Supplementary Figure S8**. High SDI regions had the highest cancer incidence (46.2%), followed by high-middle (22.2%), middle (21.5%), low-middle (7.4%), and low SDI (2.7%). Cancer deaths were most concentrated in middle SDI regions (29.1%), followed by high (27.0%), high-middle (26.5%), low-middle (12.5%), and low SDI (4.9%). ASIR decreased with SDI level, highest in high SDI regions. ASMR peaked in high-middle SDI, followed by high, middle, low, and low-middle SDI. ASPR showed a similar declining trend.

Cancer types exhibit significant variations in ASMR, ASIR, ASPR, and ASDR across SDI groups (**Figure 4**). Cancer types varied across SDI levels. TBL cancer had the highest ASMR in high and high-middle SDI (27.3 and 32.4) and the lowest in low SDI. Colorectal and stomach cancers showed similar patterns. Nonmelanoma skin cancer had the highest ASIR in high SDI (245.5). Colorectal, breast, TBL, and prostate cancers had higher ASIRs in higher SDI groups. Neuroblastoma, mesothelioma, and eye cancer remained low across all SDI levels. Breast cancer showed high prevalence across all SDI groups; prostate cancer was also highly prevalent in high SDI. TBL cancer had the highest ASDR globally, followed by colorectal, stomach, and breast cancers. Neuroblastoma, mesothelioma, and eye cancer had the lowest ASDRs. **Supplementary Figure S9** shows the correlation between SDI and cancer incidence, mortality, prevalence, and DALYs across regions.

### 3.5 Global cancer burden: country and region comparisons

**Figure 5:**
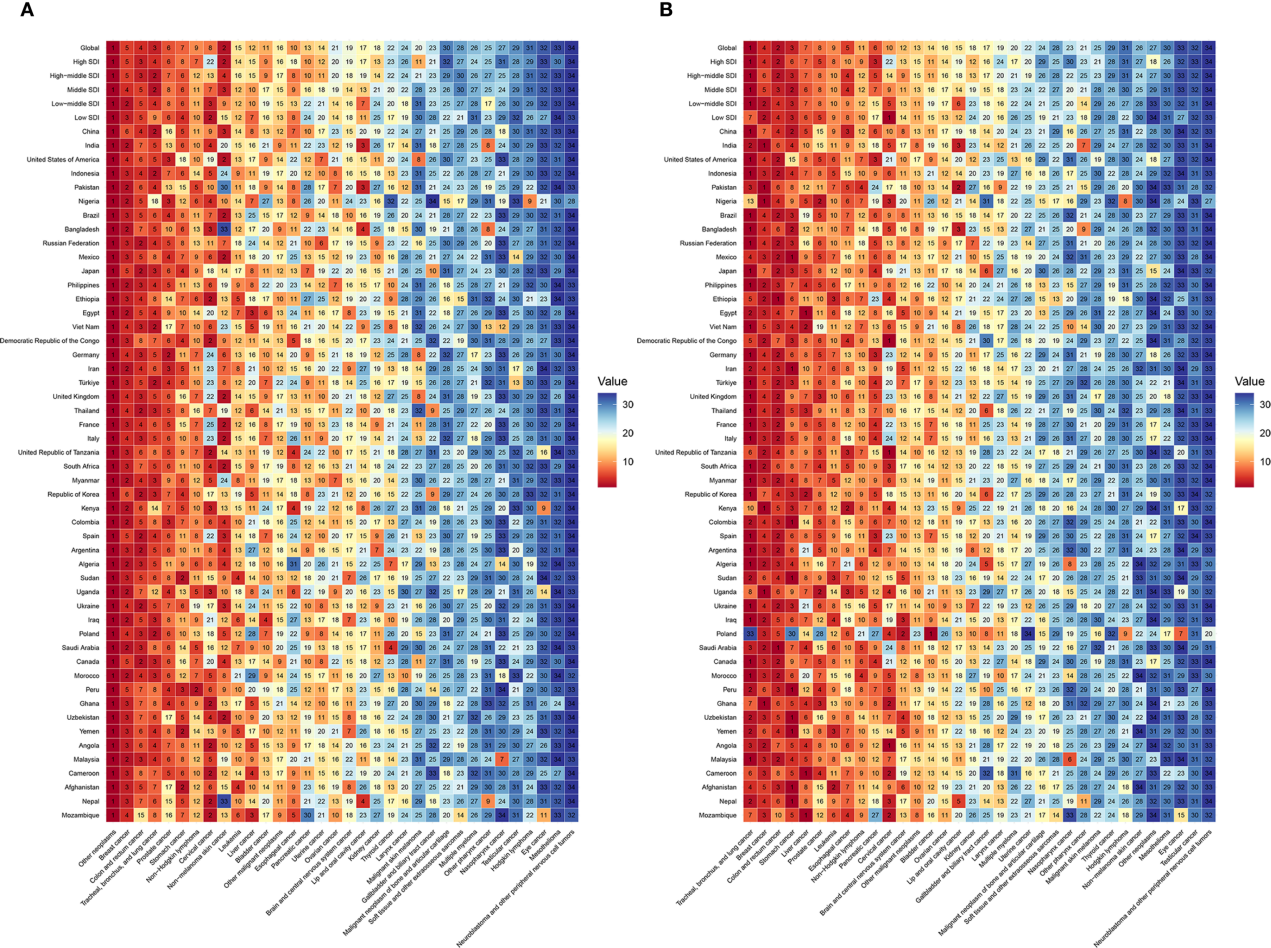
34 specific cancer types ranked by incident cases **(A)** and deaths **(B)** in both sexes, globally, by sociodemographic index status, and in the 50 most populous countries in 2021.

**Figure 6:**
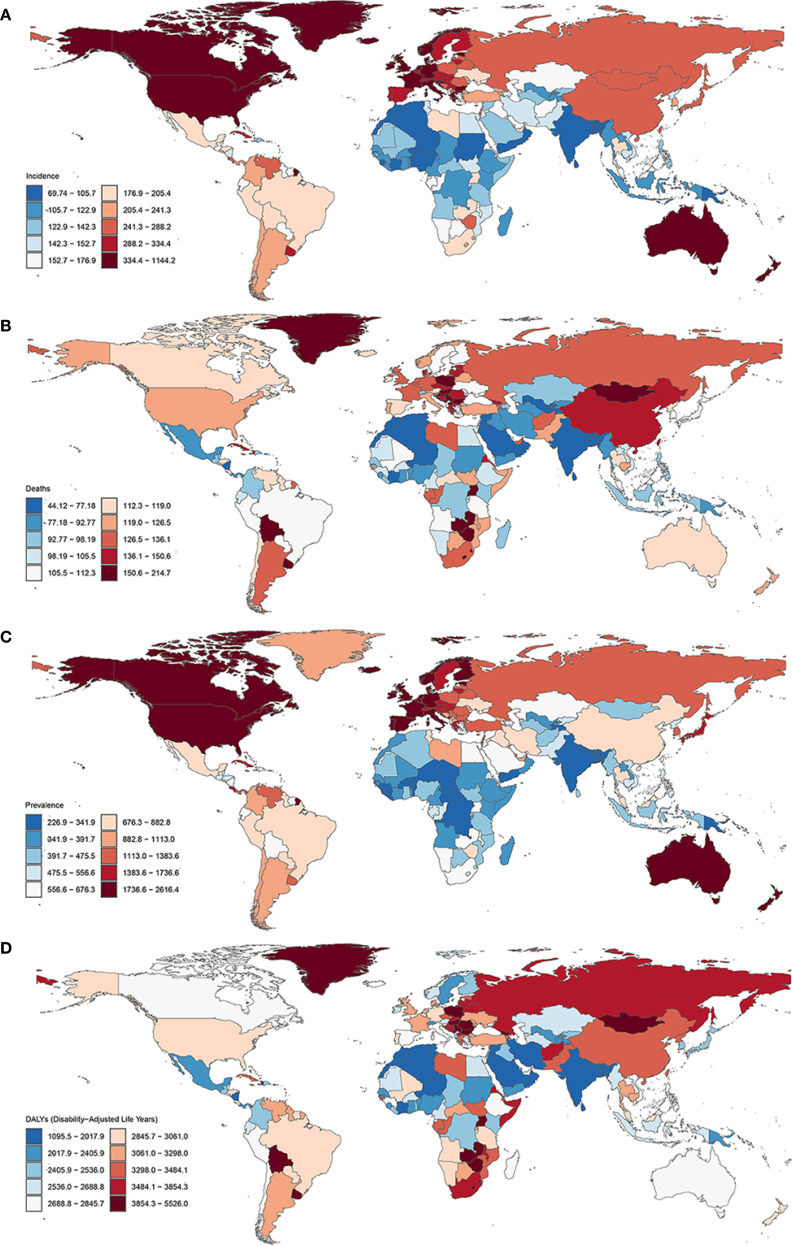
Global maps of age-standardized cancer incidence **(A)**, mortality **(B)**, prevalence **(C)**, and disability-adjusted life years (DALYs) **(D)** for total cancer, both sexes combined, in 2021.

The cancers were ranked by the number of incident cases and deaths in both sexes globally, by SDI, and in the fifty most populous countries in 2021 (**Figure 5**). Global maps of cancer incidence, mortality, prevalence, and DALYs for total cancer, both sexes combined, in 2021, are shown in **Supplementary Figure S10**. In 2021, the countries with the highest cancer incidence were the United States (6.53 million cases, accounting for 27.7% of the global total), China (5.33 million, 22.6%), India (1.18 million, 5.0%), Japan (894,000, 3.8%), and Russia (647,000, 2.7%). The highest cancer mortality was reported in China (2.81 million deaths, 28.6%), followed by India (853,000, 8.7%), the United States (705,000, 7.2%), Japan (458,000, 4.7%), and Russia (311,000, 3.2%). The highest cancer prevalence and DALY losses were observed in China, the United States, and India. Geographic disparities in cancer statistics are also evident in the age-standardized rates (ASMR, ASIR, ASPR, and ASDR) (**Figure 6**). For instance, Mongolia had the highest ASMR at 214.7, while Oman reported the lowest at 44.1. The USA, Monaco, and New Zealand recorded the highest ASIR and ASPR values, with the USA leading in both categories. ASDRs were most severe in Mongolia (5526.0) and Zimbabwe (5381.8), while the lowest were found in Oman (1095.5) and Maldives (1146.9). Correlations with the SDI revealed strong positive associations between SDI and cancer incidence (ASIR) and prevalence (ASPR), while cancer mortality (ASMR) showed a weaker correlation. The ASDR, however, did not show a significant relationship with SDI, highlighting notable regional differences in healthcare systems and cancer burden (**Supplementary Figures S11–S14**).

### 3.6 Forecasting the global cancer burden to 2050

**Figure 7:**
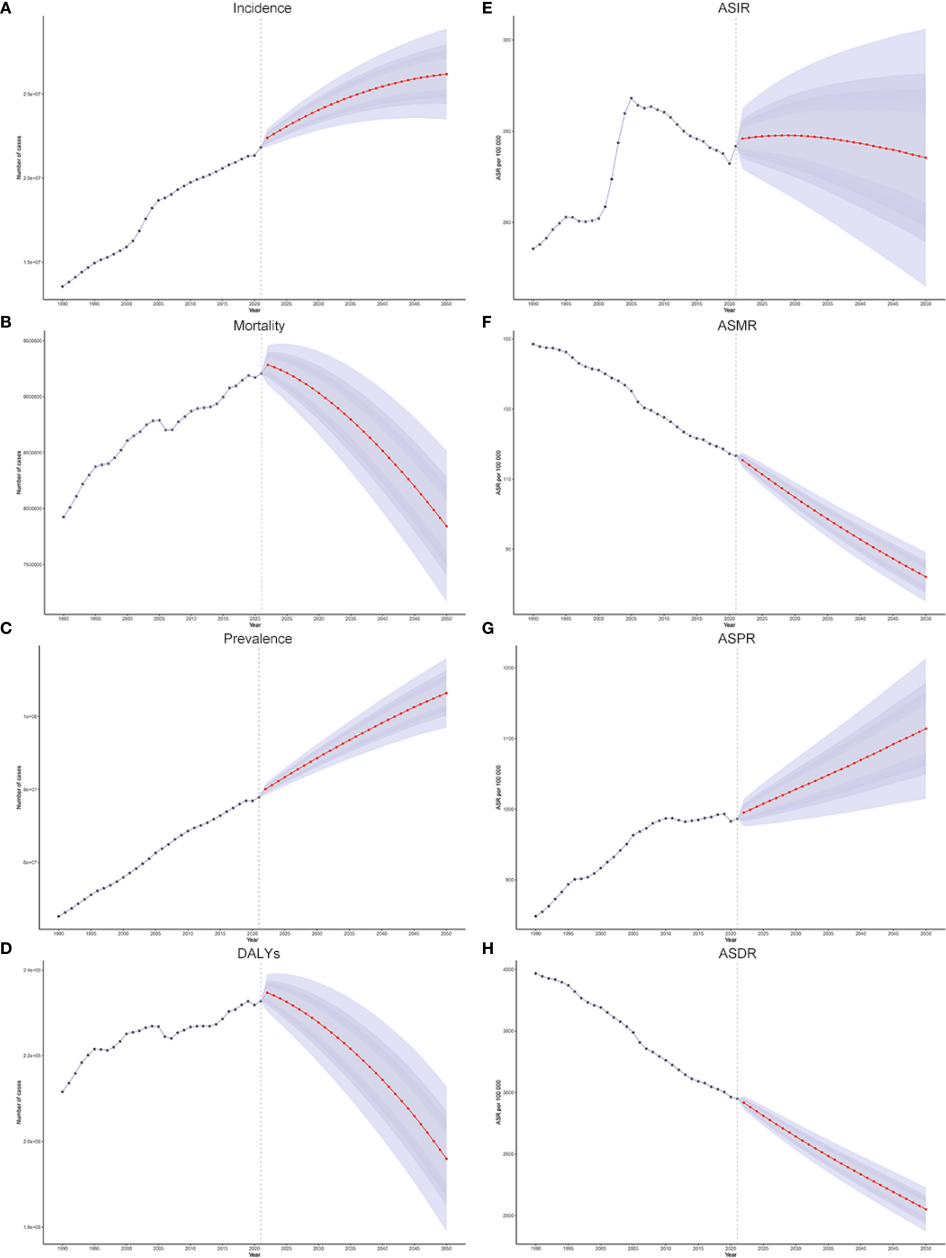
**(A–D)** The historical trends and future projections of global cancer from 1990 to 2050: incidence **(A)**, mortality **(B)**, prevalence **(C)**, and DALYs **(D)**. **(E–H)** The historical trends and future projections of global cancer ASIR, ASMR, ASPR, and ASDR from 1990 to 2050.

The evolving patterns of cancer burden and the factors influencing health outcomes are of significant concern to policymakers and the general public. The global cancer burden is projected to rise significantly by 2050, with a 19.9% increase in incidence, from 21.8 million cases in 2021 to 26.2 million cases (**Figure 7**, **Supplementary Table S5**). Prevalence is expected to grow even more, by 36.7%, reaching 106.4 million cases in 2050 (**Supplementary Table S6**). Cancer mortality and DALYs are projected to decrease by 2050. In 2021, there were 9.21 million cancer deaths and 232.72 million DALYs globally. By 2050, these numbers are expected to drop to 7.84 million deaths and 195.94 million DALYs, reflecting reductions of 14.8% and 15.8%, respectively (**Figure 7**, **Supplementary Tables S7, S8**). By 2050, global age-standardized rates (ASIR, ASMR, ASPR, ASDR) will also show mixed trends. While ASIR, ASMR, and ASDR are expected to decline by 0.9%, 29.6%, and 30.5%, respectively, ASPR will increase by 12.9%. Our analysis suggests that in 2021, the global cancer burden was higher in males than in females. The cancer burden is higher in males, and this disparity is expected to continue. In 2050, males are projected to account for more than half of global cancer incidence, mortality, prevalence, and DALYs, surpassing females in all categories (**Supplementary Figure S15**). Between 2021 and 2050, cancer incidence and prevalence are expected to rise in individuals aged 60 and older, while declining in those aged 19 and younger. Similarly, cancer-related deaths and DALYs are projected to increase in individuals aged 60 and above, while decreasing in those younger than 50 (**Supplementary Figures S16, S17**). The predicted burden of major cancers (TBL cancer, colon and rectum cancer, breast cancer, stomach cancer, liver cancer, pancreatic cancer, prostate cancer, and thyroid cancer) is presented in **Supplementary Figures S18–25**.

## 4 Discussion

This study assessed the burden of 34 major cancers across 204 countries and territories from 1990 to 2021. In 2021, there were 23.57 million new cancer cases and 9.83 million deaths, largely driven by population growth and aging. While ASIR increased slightly, ASMR declined, reflecting improvements in early detection and treatment. Men had higher rates than women, and most cases occurred in individuals aged 50 and older. High-income regions had the highest ASIRs, whereas low-SDI regions, especially East Africa, had the highest ASMRs due to limited healthcare access. By 2050, cancer incidence is projected to rise by 19.9% and prevalence by 36.7%, while mortality and DALYs are expected to decline. These findings highlight progress in reducing cancer mortality but emphasize the need for equitable healthcare strategies to address disparities across regions, sex, and socioeconomic groups.

From 1990 to 2021, the global cancer burden significantly increased, with new cases increasing 2.3-fold and deaths increasing 1.7-fold. However, the ASIR increased by only 8.8%, whereas the ASMR decreased by 21.5%. Similar results have been confirmed in previous studies (11, 12). The global increase in the ASIR for cancer is largely due to population ageing, as older populations are more susceptible to cancer (13–15). Improvements in diagnostic technologies and screening programs have also led to more cancers being detected (16, 17). Additionally, lifestyle factors, such as smoking, poor diet, and physical inactivity, contribute to the increasing incidence (18–20). On the other hand, advancements in diagnostic and therapeutic technologies, including advanced imaging, molecular diagnostics, targeted therapies, and immunotherapies, have significantly reduced cancer mortality, improved cure rates, and extended survival for advanced-stage patients (21–23). These technological improvements, combined with progress in early detection, public health interventions, and enhanced healthcare systems, are key drivers of the declining ASMR (24–26). There are notable sex differences in cancer burden, with men experiencing higher incidence and mortality rates than women. These disparities may be linked to factors such as higher smoking prevalence among men, greater occupational exposure to carcinogens, differences in genetic and epigenetic factors, and hormone levels that influence cancer susceptibility (27–30). A significant portion of cancer cases occur in individuals aged 50 years and above, and the growing proportion of high-risk groups due to ageing, coupled with population growth, has greatly contributed to the increase in absolute cancer cases, particularly in low- and middle-income countries (31). Furthermore, there are significant differences in the contributions of various cancer types. Nonmelanoma skin cancer, TBL cancer, colon and rectum cancer, and breast cancer are the most common types of cancer globally, while TBL cancer, stomach cancer, breast cancer, and liver cancer account for the highest mortality rates. This highlights the need to tailor prevention, diagnosis, and treatment strategies by cancer type. In addition, cancer burden patterns vary by income level. High-income countries report higher incidence rates due to ageing populations and widespread screening, whereas low-income countries face higher mortality rates due to limited healthcare resources and late-stage diagnoses. However, emerging risk factors, including obesity, sedentary behavior, and environmental pollution, are gaining prominence and may drive future cancer burdens, underscoring the complex interplay of ageing, medical progress, and lifestyle changes in shaping global cancer trends. Similar to trends predicted by previous research (32–34), global cancer incidence and prevalence are projected to rise significantly by 2050, primarily among individuals aged 60 years and older, underscoring a substantial burden.

Significant geographical disparities exist in the global cancer burden (32, 35), highlighting the diverse impacts of socioeconomic development, healthcare infrastructure, and exposure to environmental and behavioral risk factors. High-income countries typically exhibit higher ASIRs and ASPRs, reflecting widespread access to cancer screening, early diagnosis, and longer survival among patients. For example, the ASIRs for breast and prostate cancer in High-income North America rank among the highest in the world. However, these regions have relatively low ASMRs and ASDRs, highlighting the effectiveness of advanced cancer management and treatment strategies that reduce mortality and improve patient outcomes. In contrast, low- and middle-income countries face disproportionately higher cancer mortality rates and disease burdens, as evidenced by their elevated ASMRs and ASDRs. This disparity is particularly pronounced in Sub-Saharan Africa, where liver and cervical cancer burdens remain high owing to the prevalence of HBV and HPV infections, compounded by limited access to vaccination programs and insufficient healthcare resources. Moreover, disparities exist even within the same regions, reflecting localized variations in risk factors and healthcare access. For example, High-income Asia Pacific, Japan, and South Korea report significantly higher gastric cancer burdens than neighboring nations, driven by dietary patterns and higher rates of *Helicobacter pylori* infection (36). These regional and national differences underscore the need for tailored cancer prevention and control strategies that address specific risk factors and healthcare challenges in each context.

Sex and age are critical determinants of the global cancer burden. In 2021, men experienced a 1.2-fold higher cancer incidence and 1.3-fold higher mortality than women did, with significantly higher rates of cancers such as TBL cancer, stomach cancer, and colon and rectum cancer among men (32, 37–39). This disparity is driven by multiple factors. Men are more likely to engage in high-risk behaviors such as smoking, excessive alcohol consumption, and occupational exposure to carcinogens, all of which increase their cancer risk (40–42). Additionally, men’s lower health awareness and reduced participation in routine screenings often lead to later-stage diagnoses, resulting in poorer outcomes. Biological differences, including variations in hormone levels, may further influence susceptibility to certain cancers and their progression rates, adding complexity to the observed disparities (43, 44). Age also plays a crucial role in shaping the global cancer burden, as the disease is closely associated with the human life cycle. In children and adolescents, leukemia is the primary contributor to the cancer burden, highlighting the importance of early diagnosis and specialized pediatric care (45). While leukemia is more common among children and adolescents, the greatest cancer burden is borne by individuals aged 50 years and above, who account for the majority of new cases and deaths worldwide. Notably, individuals aged 70 years and above have the highest incidence and mortality rates for several cancers, including TBL cancer, colon and rectum cancer, stomach cancer, and prostate cancer, underscoring the profound impact of cancer on the older adult population. This age-related pattern reflects the cumulative effects of prolonged exposure to carcinogens, biological ageing, and reduced immune surveillance, highlighting the importance of targeted prevention and care strategies for ageing populations (46, 47). Understanding the interplay between sex and age in cancer epidemiology is essential for developing comprehensive approaches to reduce disparities and address the unique challenges faced by different demographic groups.

The relationship between socioeconomic development and cancer burden, as analyzed through SDI stratification, reveals complex and contrasting patterns. Studies have revealed that countries with high SDIs have significantly higher cancer incidence and prevalence rates, whereas those with low SDIs have greater burdens on cancer mortality and DALYs (9, 48–50). In addition, in the past decade, low- and low-middle-SDI regions have experienced faster increases in cancer cases and deaths than high-SDI regions. Projections align with this trend, indicating that by 2040, over two-thirds of global cancer cases are expected to occur in low- and middle-income countries, highlighting the shifting global burden of cancer (2). High-SDI countries demonstrate the highest ASIRs and ASPRs, reflecting widespread access to advanced healthcare systems, early diagnostic technologies, and effective treatment options. These nations benefit from well-established cancer management programs, resulting in relatively low ASMRs and ASDRs. For example, prostate and breast cancers have the highest ASPRs in high-SDI countries, yet their mortality rates remain comparatively low, highlighting the effectiveness of prevention and treatment strategies in these settings. Conversely, low-SDI countries face substantial challenges, including the highest mortality and DALYs, which stem from limited healthcare infrastructure, delays in diagnosis, and a lack of comprehensive preventive measures. Liver cancer and cervical cancer, in particular, exhibit significantly higher mortality rates in low-SDI countries than in their high-SDI counterparts, underscoring the impact of inadequate vaccination coverage, insufficient access to screening programs, and limited treatment options (51, 52). These disparities reflect the pressing need for targeted interventions and equitable healthcare resource allocation to address the disproportionate burden borne by low-SDI nations. Understanding these contrasting patterns is essential for tailoring global cancer prevention and control efforts to the specific needs and capacities of countries at different levels of socioeconomic development.

Despite being based on high-quality GBD data and advanced modelling methods and providing a systematic analysis of global cancer burden trends from 1990 to 2021, this study has several limitations that may affect the interpretation and application of its results. First, the data quality and sources are limited in low-income countries and regions with insufficient data, especially in areas with low cancer registry coverage, leading to increased uncertainty in some estimates. Additionally, differences in data collection standards and reporting practices across countries may impact the accuracy of cross-country comparisons. Second, while the modelling methods and time series analysis used in this study are efficient, they may not fully capture the nonlinear changes in cancer burden and their complex driving factors, such as short-term fluctuations caused by the introduction of screening policies or treatment technologies. Moreover, the study provides limited discussion on the role of specific risk factors (e.g., smoking, alcohol consumption, and obesity) and social policies (e.g., tobacco control and vaccination) and does not sufficiently assess the impact of treatment advances on patient survival and quality of life. Furthermore, external factors such as the disruption of cancer screening and treatment due to the COVID-19 pandemic, environmental risks due to climate change, and the influence of cultural and behavioral differences on cancer patterns have not been fully incorporated into the analysis framework (53, 54). Non-melanoma skin cancer (NMSC) contributes substantially to incident cases in several regions. Nevertheless, its ascertainment varies considerably across cancer registries, with some providing comprehensive reporting while others underreport or exclude NMSC altogether. Such heterogeneity may introduce bias into incidence estimates and complicate cross-country comparisons. Accordingly, while our analysis offers valuable insights into the global and regional cancer burden, trends involving NMSC should be interpreted with caution. Although our analysis does not show marked deviations in cancer incidence growth rates following the onset of the COVID-19 pandemic, this observation should be interpreted cautiously. The pandemic likely affected cancer detection and reporting through multiple mechanisms, including disruptions in routine screening programs, delays in diagnostic services, changes in health-seeking behavior, and potential lag in registry reporting. These factors could mask short-term fluctuations or temporarily reduce observed incidence, particularly in regions where healthcare systems were heavily impacted. Therefore, while the observed post-2019 trends appear broadly consistent with pre-pandemic patterns, we refrain from concluding that COVID-19 had no substantial impact on cancer incidence growth, and emphasize that ongoing monitoring and further analyses will be necessary to fully understand the pandemic’s effects. Nevertheless, these limitations do not diminish the value of this study in revealing the spatiotemporal patterns of the global cancer burden but rather provide direction for future research to optimize data sources, explore driving factors in greater depth, and strengthen cross-disciplinary discussions.

On the basis of the findings of this study, several key strategies are recommended to advance global cancer prevention and control efforts. First, expanding vaccination programs targeting HBV and HPV is crucial, as these initiatives have the potential to substantially lower the incidence and mortality of related cancers, including liver and cervical cancer. Second, enhancing screening and early diagnostic capabilities is essential, particularly for breast, cervical, pancreatic, and colorectal cancers (55, 56). This effort is especially important in resource-limited settings, where access to timely and effective diagnostic tools remains a significant challenge. Third, optimizing cancer treatment and improving resource allocation are vital. Expanding access to advanced therapies, such as targeted treatments and immunotherapies, in low-SDI countries, along with bolstering international support to enhance healthcare infrastructure, can help reduce disparities in cancer outcomes. In addition to these medical interventions, raising public health awareness through education campaigns is necessary to address modifiable risk factors. These campaigns should focus on reducing smoking, alcohol consumption, and unhealthy diets while promoting physical activity to mitigate cancer risk. Finally, the establishment and enhancement of cancer registries in underrepresented regions are imperative for generating reliable data to inform policy decisions and resource distributions. Recent advancements in the Internet of Things (IoT) have demonstrated considerable potential in the field of cancer prevention. Through the integration of wearable sensors, mobile health platforms, and real-time data analytics, IoT technologies enable continuous monitoring of lifestyle behaviors, facilitate the early detection of warning signals, and enhance adherence to cancer screening and follow-up programs. Moreover, the emerging concept of the Internet of Surgical Things (IoST) highlights how IoT can be embedded in surgical practice, supporting telesurgery, surgical telementoring, image-guided procedures, and telemonitoring of patients (57). While IoST has primarily been applied to improve surgical precision and perioperative care, these advances also illustrate the broader capacity of IoT-based systems to enhance early detection, optimize treatment pathways, and strengthen long-term surveillance in oncology. Collectively, these strategies emphasize the need for a multifaceted and equitable approach to cancer prevention and control, addressing both systemic healthcare barriers and individual-level behaviors to reduce the global burden of cancer.

Future research on global cancer trends should prioritize improving data quality, especially in low-SDI countries, to strengthen cancer registry systems and ensure more accurate and comprehensive data. This would facilitate better tracking of incidence and mortality rates over time, enabling targeted interventions. Understanding the complex interplay between behavioral, genetic, and environmental risk factors is also crucial for identifying modifiable risks and developing effective prevention strategies. Furthermore, evaluating existing healthcare interventions—such as HPV vaccination, smoking cessation, and cancer screening programs—can offer insights into their effectiveness in reducing the cancer burden, particularly in resource-limited settings. Merging strategies such as liquid biopsies, including circulating tumor DNA, with polygenic risk scores (PRS) for risk stratification offers promising avenues for the early detection of deadly cancers (55, 56). Nonetheless, their clinical implementation entails important ethical and practical considerations, including informed consent, data privacy, equitable access, and potential psychosocial impacts (58). Rigorous validation and careful evaluation will be essential to fully realize the potential of these approaches in personalized cancer prevention and early detection. As cancer trends evolve, focusing on emerging cancers with increasing incidence rates, such as pancreatic and thyroid cancers, is essential. Research should investigate the underlying causes of these trends and develop targeted therapies for early detection and treatment. A multidisciplinary approach combining epidemiology, genomics, and health economics is necessary to create comprehensive cancer control strategies. Finally, the COVID-19 pandemic may continue to affect estimates of cancer burden trends in the coming years, with reporting delays and the ongoing impact of the pandemic. Future research should focus on gaining a comprehensive understanding of the effects of the 2022 pandemic on cancer burden and its long-term implications. These research directions are critical for addressing disparities, improving prevention, and tailoring treatment approaches, ultimately enhancing cancer outcomes and quality of life for patients worldwide.

## 5 Conclusion

Cancer is a complex group of diseases with diverse risk factors, mechanisms, and treatment strategies, making it a challenge to understand and manage. The GBD 2021 study provides updated global cancer burden estimates, revealing significant disparities by sex, age, region, country, and SDI. By 2050, global cancer incidence is expected to exceed 26 million, with prevalence surpassing 100 million. However, cancer mortality is projected to decrease by 14.8%, and DALYs by 15.8%. These findings underscore the need for personalized cancer control strategies based on regional and socioeconomic contexts. Policymakers can use this data to guide resource allocation and implement targeted prevention efforts. Addressing sex- and age-related disparities is crucial for improving cancer care. However, reducing the global cancer burden requires coordinated efforts across policy, research, and international collaboration. The long-term effects of the COVID-19 pandemic on cancer remain uncertain, highlighting the need for further research to refine cancer control strategies in the post-pandemic era.

## Data Availability

The original contributions presented in the study are included in the article/**Supplementary Material**. Further inquiries can be directed to the corresponding author.
