# Supplementary material for: Global, regional, and national burden of 34 cancer groups across 204 countries and territories, 1990–2021, and projections to 2050: a systematic analysis of the Global Burden of Disease Study 2021
Source: Front Oncol. 2025 Oct 22;15:1660125. doi: 10.3389/fonc.2025.1660125 (PMC12586085; doi:10.3389/fonc.2025.1660125)
Supplement: Supplementary file 1 [file DataSheet1.docx]

**Supplementary material**

1. **Supplementary Materials** (Methods)
2. **Supplementary Results** (Assessment of the burden associated with major cancers)
3. **Table S1**. Global incidence and deaths in 2021 for total cancers and 34 cancer groups by gender
4. **Figure S1** Trends in global cancer metrics (1990–2021) by sex
5. **Table S2** Age-standardised global cancer incidence (ASIR), mortality (ASMR), prevalence (ASPR), and disability-adjusted life years (ASDR) for 34 specific cancer types in 2021, by sex
6. **Figure S2** Global cancer incidence, mortality, prevalence, and disability-adjusted life years (DALYs), along with age-standardized incidence, mortality, prevalence, and DALYs by age group and sex in 2021
7. **Figure S3** Age-specific global contributions of 34 cancer types to total cancer incidence (A), mortality (B), prevalence (C), and disability-adjusted life years (DALYs) (D) in 2021
8. **Table S3** Global incidence and deaths in 2021 for total cancers and 34 cancer types by age group
9. **Table S4** Global incidence, prevalence, deaths, and DALYs in 2021 for 34 cancer types by age group
10. **Figure S4** 34 specific cancer types ranked by the number of incident cases globally and across 21 global regions in 2021
11. **Figure S5** 34 specific cancer types ranked by the number of deaths globally and across 21 global regions in 2021
12. **Figure S6** 34 specific cancer types ranked by the number of prevalent cases globally and across 21 regions in 2021
13. **Figure S7** 34 specific cancer types ranked by DALYs losses globally and across 21 regions in 2021
14. **Figure S8** Trends in global cancer incidence, mortality, prevalence, and disability-adjusted life years (DALYs), along with age-standardized incidence, mortality, prevalence, and DALYs from 1990 to 2021, by sociodemographic index status
15. **Figure S9** Correlations of the sociodemographic index with age-standardized global cancer incidence, mortality, prevalence, and DALYs across 21 global regions in 2021
16. **Figure S10** Global maps of cancer incidence, mortality, prevalence, and disability-adjusted life years (D) for total cancer, both sexes combined, in 2021
17. **Figure S11** Correlations of the sociodemographic index with age-standardised global cancer incidence for total cancers in 204 countries and territories
18. **Figure S12** Correlations of the sociodemographic index with age-standardised global cancer prevalence for total cancers in 204 countries and territories
19. **Figure S13** Correlations of the sociodemographic index with age-standardised global cancer mortality for total cancers in 204 countries and territories
20. **Figure S14** Correlations of the sociodemographic index with age-standardised global cancer DALYs for total cancers in 204 countries and territories
21. **Table S5** Global cancer incidence projections: 1990–2050
22. **Table S6** Global cancer prevalence projections: 1990–2050
23. **Table S7** Global cancer deaths projections: 1990–2050
24. **Table S8** Global projections of cancer-related DALYs: 1990–2050
25. **Figure S15** The historical trends and future projections of global cancer from 1990 to 2050: incidence, mortality, prevalence, and DALYs, by sex
26. **Figure S16** The historical trends and future projections of global cancer incidence and prevalence from 1990 to 2050, by age group
27. **Figure S17** The historical trends and future projections of global cancer mortality and disability-adjusted life years (DALYs) from 1990 to 2050, by age group
28. **Figure S18** Projected burden of tracheal, bronchus, and lung cancer
29. **Figure S19** Projected burden of breast cancer
30. **Figure S20** Projected burden of colon and rectum cancer
31. **Figure 21** Projected burden of stomach cancer
32. **Figure 22** Projected burden of liver cancer
33. **Figure 23** Projected burden of pancreatic cancer
34. **Figure 24** Projected burden of prostate cancer
35. **Figure 25** Projected burden of thyroid cancer

**Supplementary Materials (**Methods**)**

**Estimation methods**

The general estimation methods of the GBD 2021, as well as the approach used to estimate the cancer burden, have been previously described [1-3]. In summary, this information is derived from vital registration systems, population-based cancer registries, and verbal autopsy studies. To ensure data consistency, various statistical techniques, such as noise-reduction algorithms, garbage code redistribution, and misclassification correction, have been employed to minimize data heterogeneity. To assess mortality by factors such as age, sex, location, and year, a cause-of-death ensemble model (CODEm) was used. This model employs Bayesian geospatial regression analysis, a statistical technique that accounts for spatial relationships between data points. CODEm uses an ensemble of statistical models while also systematically testing combinations of covariates on the basis of their out-of-sample predictive validity. It then combines the results to estimate the number of deaths from a given cause by location, age, sex, and year. CODEm is run separately for countries and regions with and without extensive vital registration data to mitigate the potential for uncertainty inflation due to highly heterogeneous data. Multiple iterations of out-of-sample predictive validity are assessed for each model, and the models with the minimum root mean square error are weighted to generate the ensemble model for the given cause. The GBD study provides a straightforward quality assessment tool that assigns a score ranging from 0 to 5 to evaluate the quality of data provided by each country or region. To categorize countries on the basis of their level of development, the GBD 2021 study used the sociodemographic index (SDI), a composite measure of lag distributed income per capita, average years of education for those aged 15 years or older, and fertility rates among females younger than 25 years.

**Projection methods**

Numerous methods, such as the age-period-cohort (APC) model, Joinpoint model, and Poisson regression, have been extensively applied to predict cancer incidence and mortality based on cancer registry data [4, 5]. To identify the most accurate predictive model, we conducted a comprehensive comparison of several approaches, including the Bayesian age-period-cohort (BAPC) model, generalized additive model, smooth spline model, Joinpoint model, and Poisson regression, utilizing cancer case data at both global and national levels. Through detailed analysis, the BAPC model demonstrated the lowest absolute percentage deviation, highlighting its superior predictive performance. Therefore, we selected the BAPC model to project cancer incidence rates and case numbers through 2050.

**Statistical analyses**

The estimates for the incidence of new cases and deaths were reported with 95% uncertainty intervals (UIs), calculated as the 2.5th and 97.5th ranked values from a posterior distribution of 1000 draws. ASRs were calculated via the direct method on the basis of population estimates. The Joinpoint regression program V.4.6.1.0 (Statistical Research and Applications Branch, National Cancer Institute, Bethesda, Maryland, USA) was used to estimate the average annual percent change (AAPC) and corresponding 95% CI in age-standardised rates. When both the annualised rate of change and the lower boundary of the 95% CI were positive, this was considered an increasing trend. We employed age-standardised incidence rates (ASIRs) and age-standardised mortality rates (ASMRs) to assess regional patterns of morbidity and mortality across 34 cancer types. The age-standardised rates (expressed per 100,000 population) were derived via the direct method. Standardisation played a pivotal role in this analysis, as it mitigated bias when comparing proportions or rates across populations with varying age distributions. Data processing and analyses were conducted via Python, version 3.7.0 (Python Software Foundation); Stata, version 15.1 (Stata Corp); and R, version 3.4.1 (R Foundation).

**References**

1. Global incidence, prevalence, years lived with disability (YLDs), disability-adjusted life-years (DALYs), and healthy life expectancy (HALE) for 371 diseases and injuries in 204 countries and territories and 811 subnational locations, 1990-2021: a systematic analysis for the Global Burden of Disease Study 2021. Lancet 403, 2133-2161 (2024).
2. Global incidence, prevalence, years lived with disability (YLDs), disability-adjusted life-years (DALYs), and healthy life expectancy (HALE) for 371 diseases and injuries in 204 countries and territories and 811 subnational locations, 1990-2021: a systematic analysis for the Global Burden of Disease Study 2021. Lancet 403, 2133-2161 (2024).
3. Kocarnik JM, et al. Cancer Incidence, Mortality, Years of Life Lost, Years Lived With Disability, and Disability-Adjusted Life Years for 29 Cancer Groups From 2010 to 2019: A Systematic Analysis for the Global Burden of Disease Study 2019. JAMA Oncol 8, 420-444 (2022).
4. Lee TC, Dean CB, Semenciw R Short-term cancer mortality projections: a comparative study of prediction methods. Stat Med 30, 3387-3402 (2011).
5. Jürgens V, Ess S, Cerny T, Vounatsou P A Bayesian generalized age-period-cohort power model for cancer projections. Stat Med 33, 4627-4636 (2014).

**Supplementary Results** (Assessment of the burden associated with major cancers)

**Tracheal, bronchus, and lung (TBL) cancer cancer**

In 2021, TBL cancer caused a total of 2,016,547 deaths worldwide (95% UI, 1,820,498–2,218,372), with 2,280,688 new cases (95% UI, 2,063,252–2,509,740). The ASMR was 23.5 (21.2–25.8), making TBL cancer the leading cause of cancer-related deaths globally. The ASIR was 26.4 (23.9–29.1). From 1990 to 2021, the average annual percentage change (AAPC) in the ASIR (-0.071, -0.079–-0.062), ASMR (-0.136, -0.145–-0.127), and ASDR (-5.077, -5.354–-4.801) for TBL cancer showed a declining trend. However, the ASPR increased overall (0.089, 0.082–0.097), although it significantly declined between 2011 and 2021 (Fig. 1A-D). In terms of sex differences, the ASMR, ASIR, ASPR, and ASDR for TBL cancer were significantly greater in males, with values of 763.7, 37.8, 51.5, and 34.3, respectively, than in females, with values of 56.1, 10.4, 75.7, and 2.1, respectively. This disparity may be attributed to factors such as higher smoking rates and occupational exposures among men. On the basis of the number of cases and deaths, TBL cancer incidence and mortality among individuals aged 50 years and above accounted for 94.6% and 95.1% of the total, respectively. Regionally, the burden of TBL cancer varies significantly. East Asia reported the highest ASMR and ASIR, while High-income Asia Pacific recorded the highest ASPR, and Central Europe presented the highest ASDR. In contrast, Western Sub-Saharan Africa displayed the lowest values across these indicators, highlighting the influence of economic development, environmental pollution, and health behaviours on the burden of lung cancer. At the country level, the three countries with the highest TBL cancer incidence were China [934,704 (95% UI, 750,040–1,136,938)], the United States of America [223,958 (95% UI, 206,061–235,174)], and Japan [121,731 (95% UI, 105,282–131,198)], accounting for 41.0%, 9.8%, and 5.3% of the global incidence, respectively. Similarly, the three countries with the highest mortality were China [814,364 (95% UI, 652,636–987,795)], the United States of America [175,480 (95% UI, 161,001–184,735)], and Japan [92,119 (95% UI, 78,912–98,959)], accounting for 40.4%, 8.7%, and 4.6% of the global deaths, respectively. Monaco had the highest ASMR, ASIR, and ASPR, whereas Greenland presented the highest ASDR. In terms of the SDI, high-SDI countries had the highest ASPRs for TBL cancer, whereas high-middle-SDI countries presented the highest ASIRs, ASMRs, and ASDRs. Correlation analysis revealed that the ASMR (r=0.5799, p<0.001), ASIR (r=0.6968, p<0.001), ASPR (r=0.6202, p<0.001), and ASDR (r=0.7553, p<0.001) for TBL cancer were positively correlated with the SDI, suggesting that higher SDI regions tend to have greater lung cancer burdens (Fig. 1E-H).


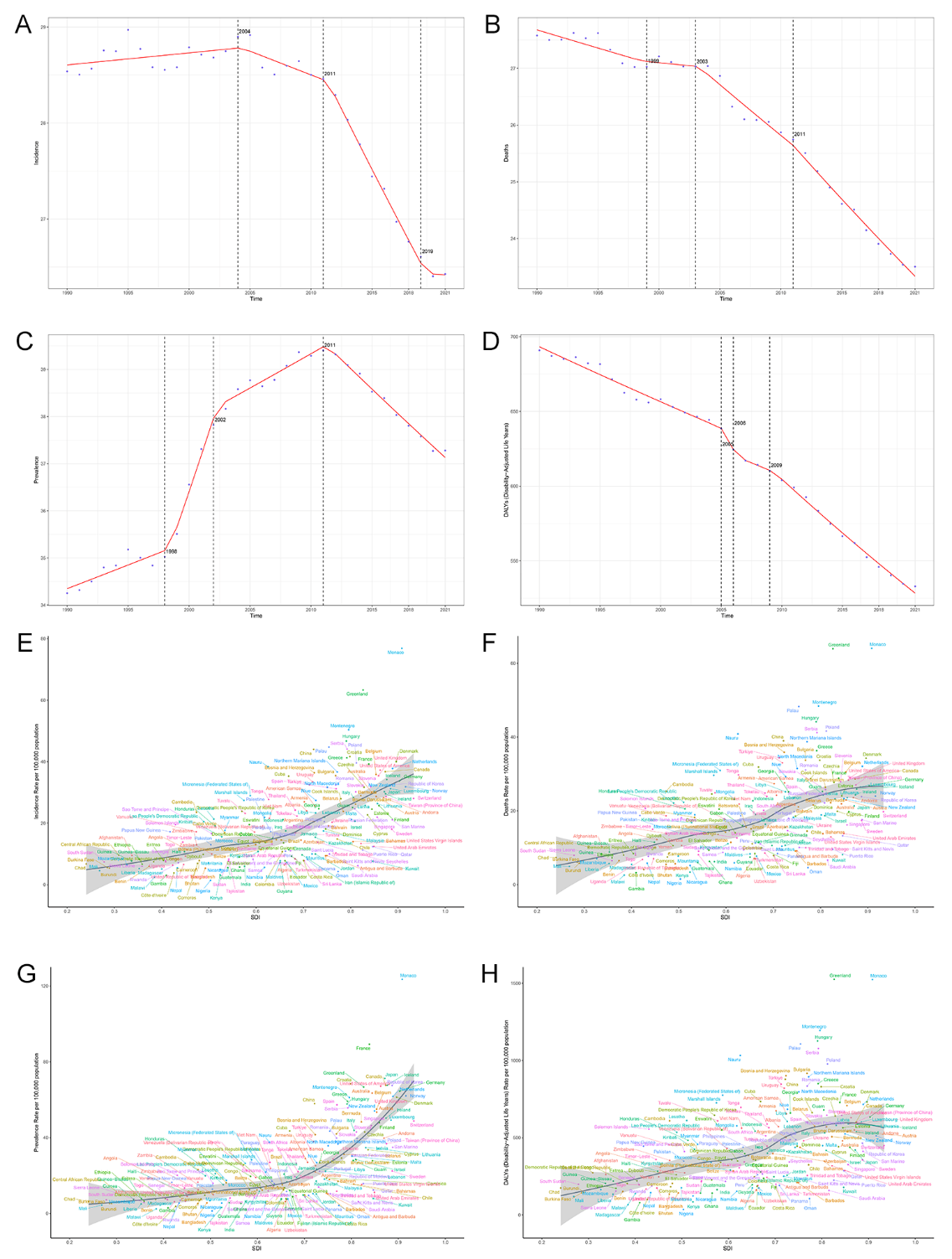


**Fig 1.** A–D Average annual percent changes in age-standardised global cancer incidence (A), mortality (B), prevalence (C), and disability-adjusted life years (DALYs) (D) in both sexes for tracheal, bronchial, and lung cancer from 1990 to 2021. E–H Correlations of the sociodemographic index with age-standardised global cancer incidence €, mortality (F), prevalence (G), and DALYs (H) for tracheal, bronchial, and lung cancer in 204 countries and territories.

**Colon and rectum cancer**

In 2021, the global incidence of colon and rectum cancer reached 2,194,143 cases (95% UI, 2,001,272–2,359,390), leading to approximately 1,044,072 deaths (95% UI, 950,188–1,120,169). The ASMR for colon and rectum cancer was 12.4 (11.2–13.3), second only to that for TBL cancer. The ASIR was 25.6 (23.3–27.5). Between 1990 and 2008, the ASIR had a significant upwards trend, with an AAPC of 0.052 (0.049–0.056), which stabilized after 2008. The ASMR (-0.103, -0.106–-0.099) and ASDR (-2.356, -2.451–-2.260) showed a consistent downwards trend in the AAPC from 1990 to 2021 (Fig. 2A-D). From 1990 to 2019, the AAPC of the ASPR (-0.136, -0.145–-0.127) exhibited a stable upwards trend, potentially indicating an increase in the overall survival rates for colon and rectum cancer patients. In terms of sex, the ASMR, ASIR, ASPR, and ASDR for colon and rectum cancer were significantly greater in men than in women, exceeding the rates in women by more than 1.5 times. In terms of age distribution, individuals aged 50 years and above accounted for 90.3% of the total incidence and 92.4% of the total mortality of colon and rectum cancer. Regionally, Central Europe presented the highest ASMR and ASDR, while High-income Asia Pacific presented the highest ASIR and ASPR. In contrast, South Asia had the lowest values for these four cancer burden indicators. The countries with the highest colon and rectum cancer incidence and mortality were China [incidence: 658,321 (95% UI, 53,995–798,063); deaths: 275,129 (95% UI, 223,379–330,960)], the United States of America [incidence: 214,115 (95% UI, 197,871–225,187); deaths: 75,087 (95% UI, 68,062–79,711)], and Japan [incidence: 171,043 (95% UI, 148,913–184,889); deaths: 67,924 (95% UI, 56,451–74,360)]. These countries accounted for 30.0%, 9.8%, and 7.8% of the global colon and rectum cancer incidence, respectively, and 26.4%, 7.2%, and 6.5% of the global colon and rectum cancer mortality, respectively. Additionally, Uruguay (27.5), Hungary (26.0), and Bulgaria (25.7) recorded the highest ASMRs for colon cancer, whereas the Netherlands (69.8), Monaco (68.3), and Bermuda (61.8) had the highest ASIRs. Gambia reported the lowest ASMR (3.0) and ASIR (3.3). From the SDI perspective, high-SDI countries had the highest ASIRs, ASPRs, and ASDRs for colon and rectum cancer, whereas high-middle-SDI countries had the highest ASMRs. Furthermore, the ASMR (r=0.6301, p<0.001), ASIR (r=0.8228, p<0.001), ASPR (r=0.8825, p<0.001), and ASDR (r=0.5637, p<0.001) for colon and rectum cancer showed significant positive correlations with the SDI (Fig. 2E-H).


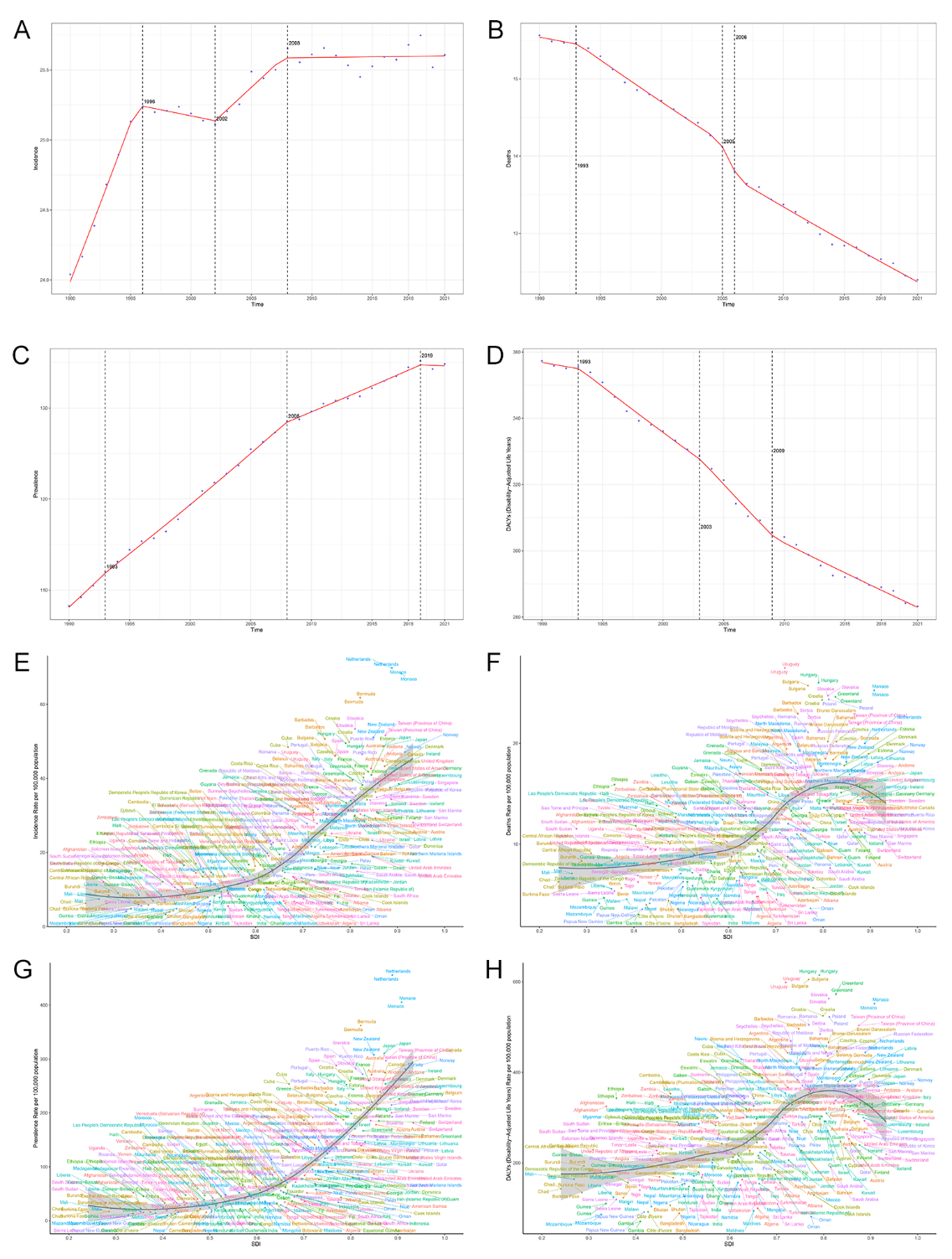


**Fig 2.** A–D Average annual percent changes in age-standardised global cancer incidence (A), mortality (B), prevalence (C), and disability-adjusted life years (DALYs) (D) in both sexes for colon and rectum cancer from 1990 to 2021. E–H Correlations of the sociodemographic index with age-standardised global cancer incidence €, mortality (F), prevalence (G), and DALYs (H) for colon and rectum cancer in 204 countries and territories.

**Breast cancer**

As shown in Table 1, there were approximately 2,121,564 new breast cancer cases (95% UI, 1,982,143–2,268,723) and 674,199 deaths (95% UI, 623,372–720,823) in 2021. The ASMR was 7.9 (7.3–8.4), and the ASIR was 24.6 (22.9–26.3). From 1990 to 2021, the AAPC for breast cancer showed a decreasing trend in the ASMR (-0.039, -0.041–-0.037) and ASDR (-0.837, -0.897–-0.776), whereas the ASIR (0.104, 0.099–0.110) and ASPR (0.664, 0.607–0.722) increased (Fig. 3A-D). The breast cancer burden among males is extremely low. The proportions of breast cancer incidence and mortality relative to the total number of cases across different age groups were as follows: 48.4% and 44.2% for individuals aged 50–69 years, 26.8% and 19.4% for those aged 15–49 years, and 24.8% and 36.4% for those aged 70 years and above. Among the 29 types of cancer, female breast cancer ranks highest in terms of the ASMR, ASPR, and ASDR, while its ASIR is second only to that of nonmelanoma skin cancer, highlighting the sex-specific impact of this disease. Regionally, the ASIRs for breast cancer are highest in High-income North America, Western Europe, and Australasia. The ASMRs are highest in Southern Sub-Saharan Africa, Western Sub-Saharan Africa, and Eastern Sub-Saharan Africa. South Asia and East Asia presented the lowest ASIRs and ASMRs, respectively. At the country level, China had the highest number of breast cancer cases [402,794 (95% UI, 312,117–505,644)], followed by the United States of America [272,387 (95% UI, 251,345–285,258)] and India [159,271 (95% UI, 135,112–187,901)], accounting for 19.0%, 12.8%, and 7.5% of the global incidence, respectively. In terms of mortality, China again ranked first, with 91,484 deaths (95% UI, 71,739–113,710), followed by India [80,817 (95% UI, 68,416–95,687)] and the United States of America [53,473 (95% UI, 47,933–56,791)], accounting for 13.6%, 12.0%, and 7.9% of global deaths, respectively. Palau (21.9), Fiji (21.1), and Nauru (21.0) reported the highest ASMRs, whereas the ASIRs were highest in Monaco (86.0), Barbados (54.7), and the Bahamas (52.8). The highest ASPRs are found in Monaco (886.3), France (582.3), and the United States of America (556.0), whereas the ASDR peaks in Nauru (640.8). Distinct differences in breast cancer burden exist across countries with varying SDI levels: the ASIRs and ASPRs are highest in high-SDI countries, whereas low-SDI countries present the highest ASMRs and ASDRs. The ASIR (r = 0.7615, p < 0.001) and ASPR (r = 0.8557, p < 0.001) for breast cancer showed a significant positive correlation with the SDI, while the ASMR (r = 0.0101, p = 0.8856) and ASDR (r = -0.0623, p = 0.3757) were not correlated with the SDI (Fig. 3E-H).


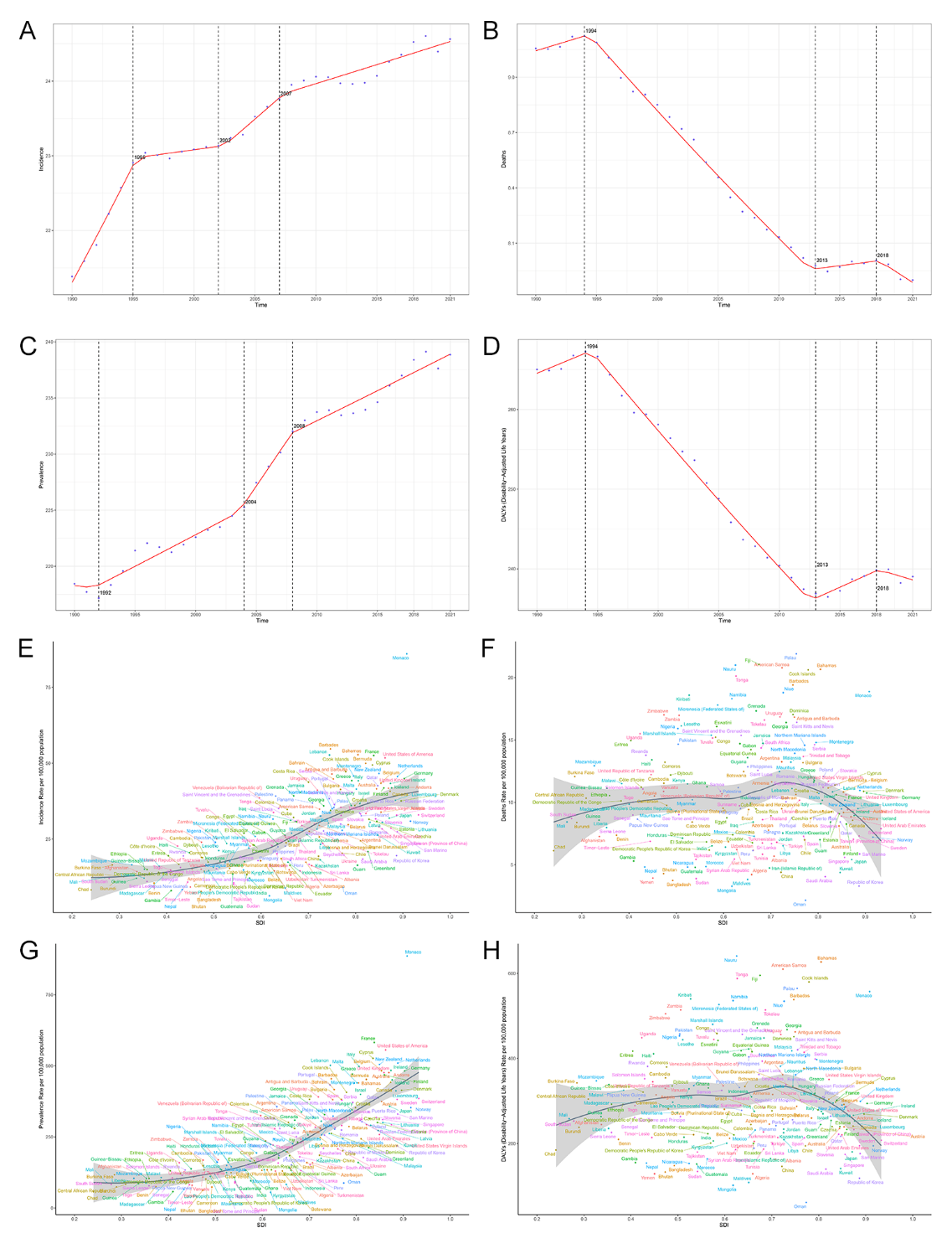


**Fig 3.** **A–D** Average annual percent changes in age-standardised global cancer incidence (**A**), mortality (**B**), prevalence (**C**), and disability-adjusted life years (DALYs) (**D**) in both sexes for breast cancer from 1990 to 2021. **E–H** Correlations of the sociodemographic index with age-standardised global cancer incidence €, mortality (**F**), prevalence (**G**), and DALYs (**H**) for breast cancer in 204 countries and territories.

**Stomach cancer**

In 2021, the global incidence of stomach cancer was 1,230,233 cases (95% UI, 1,052,350–1,409,970), with a total of 954,374 deaths (95% UI, 821,751–1,089,577). The global ASMR of stomach cancer was 11.2 (9.6–12.7), ranking only behind TBL cancer and colorectal cancer. The ASIR was 14.3 (12.2–16.4). From 1990 to 2021, there was a continuous decline in the ASIR (AAPC=-0.34, -0.347–-0.333), ASMR (AAPC=-0.353, -0.361–-0.345), ASPR (AAPC=-0.426, -0.435–-0.418), and ASDR (AAPC=-9.705, -9.886–-9.524) of stomach cancer (Fig. 4A-D). In terms of sex, the ASMR (16.0 vs. 7.1), ASIR (20.9 vs. 8.6), ASPR (41.4 vs. 15.2), and ASDR (371.2 vs. 165.6) of stomach cancer were significantly greater in males than in females, all exceeding 2.2 times those of females. In terms of age distribution, the incidence and mortality were highest among individuals aged 70 years and older, accounting for 46.5% and 51.8% of the total cases and deaths, respectively. Regionally, the three regions with the highest ASIRs were East Asia, High-income Asia Pacific, and Andean Latin America. The highest ASMR was observed in Andean Latin America (21.3). The region with the highest ASPR was High-income Asia Pacific (78). East Asia had the highest ASDR (497), followed by Andean Latin America (485) and Oceania (342). Globally, the highest stomach cancer incidence was observed in China, with 611,799 cases (95% UI, 471,966–765,562), representing 49.7% of the total. Japan ranks second, with 99,035 cases (95% UI, 85,266–106,694), accounting for 8.1% of the total, and India ranks third, with 70,163 cases (95% UI, 61,019–86,919), contributing 5.7% to the global burden. In terms of mortality, China again led with 445,013 deaths (95% UI, 344,736–555,834), equivalent to 46.6% of the global total. India recorded 68,517 deaths (95% UI, 59,455–84,302), accounting for 7.2%, while Japan reported 58,012 deaths (95% UI, 48,988–63,019), accounting for 6.1%. The country with the highest ASMR (37.4), ASIR (36.8), and ASDR (930.4) is Mongolia. The countries with the highest ASPRs are the Republic of Korea (79.4), Japan (78.9), and China (57.2). From the perspective of the SDI, countries with a high SDI had the highest ASIRs, ASMRs, ASPRs, and ASDRs for stomach cancer. Correlation analysis indicated that the SDI was negatively associated with the ASMR (r=-0.3486, p<0.001) and ASDR (r=-0.3877, p<0.001) for stomach cancer, while no significant correlation was observed for the ASIR and ASPR (Fig. 4E-H).


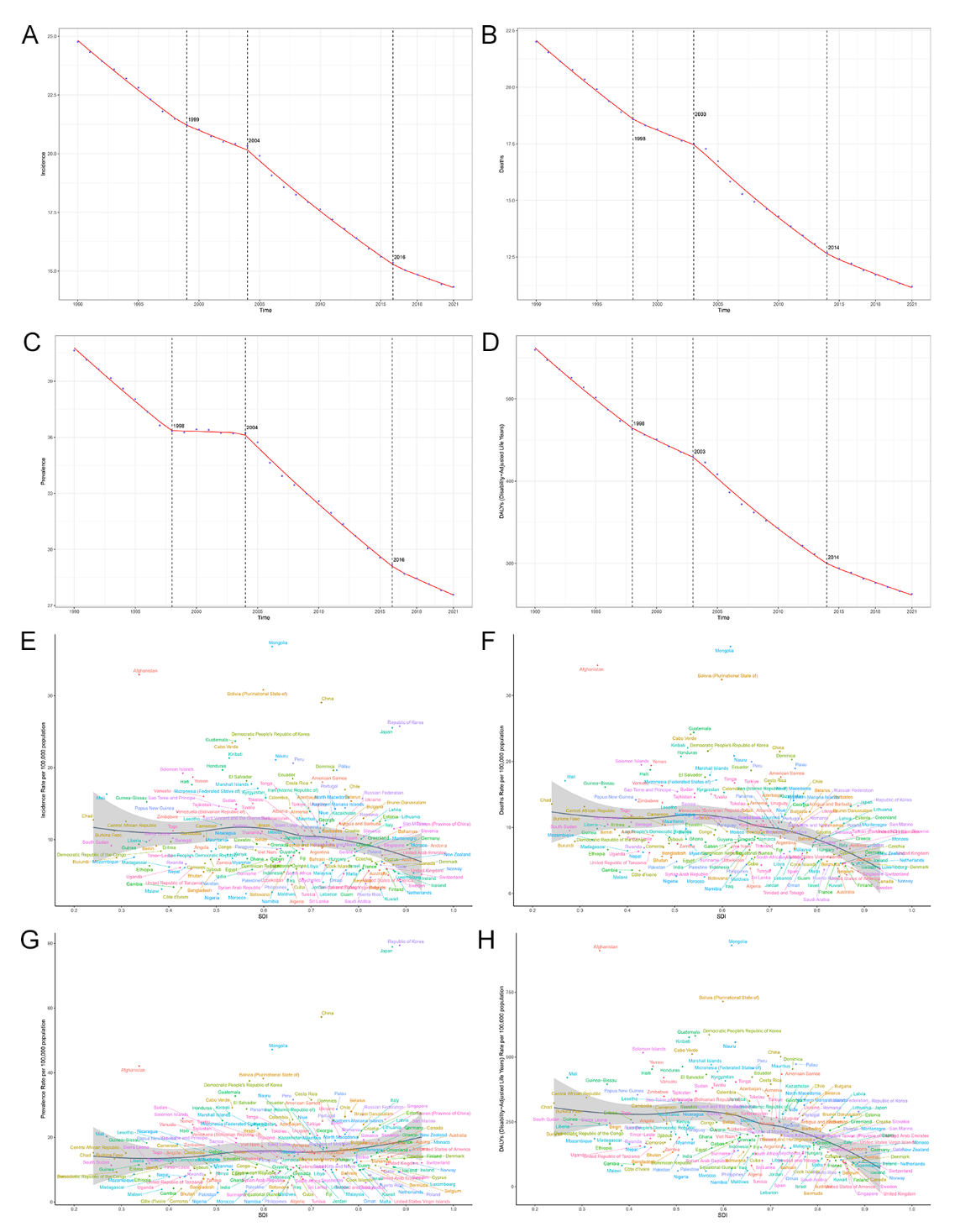


**Fig 4.** **A–D** Average annual percent changes in age-standardised global cancer incidence (**A**), mortality (**B**), prevalence (**C**), and disability-adjusted life years (DALYs) (**D**) in both sexes for stomach cancer from 1990 to 2021. **E–H** Correlations of the sociodemographic index with age-standardised global cancer incidence €, mortality (**F**), prevalence (**G**), and DALYs (**H**) for stomach cancer in 204 countries and territories.

**Liver cancer**

In 2021, there were approximately 529,202 new cases of liver cancer (95% UI, 480,339–593,849) and 483,875 deaths (95% UI, 440,400–540,177) globally. The global ASMR for liver cancer was 5.6 (5.1–6.3), and the ASIR was 6.2 (5.6–6.9). From 1990 to 2021, notable temporal trends in the ASIR, ASMR, ASPR, and ASDR for liver cancer were detected. The ASIR peaked around 2000 and subsequently declined, with a marked decrease after 2005 (AAPC = 0.006, 0.005–0.007). The ASMR followed a similar trend, declining after 2000, with a steeper decrease after 2005 (AAPC = -0.006, -0.008–-0.004). The ASPR peaked around 2000 and slightly increased from 2000 to 2014, with an AAPC of 0.028 (0.027–0.029). The ASDR increased sharply between 2000 and 2005 and then significantly declined after 2016 (AAPC = -0.745, -0.807–-0.683) (Fig. 5A-D). The ASMR (8.1 vs. 3.5), ASIR (9.0 vs. 3.6), ASPR (12.8 vs. 5.0), and ASDR (217.7 vs. 85.1) were significantly greater in males than in females, each exceeding 2.3 times that in females. In terms of age distribution, the highest incidence and mortality were observed in the 50–69 year age group, accounting for 47.6% and 45.2% of the total, respectively. Regionally, High-income Asia Pacific had the highest ASIR and ASPR for liver cancer, while Western Sub-Saharan Africa had the highest ASMR and ASDR. China reported the highest liver cancer incidence, with 196,637 cases (95% UI, 158,273–243,557), accounting for 37.2% of the global total. Japan and India followed, with 39,163 cases (95% UI, 33,437–42,580) and 33,339 cases (95% UI, 29,524–37,513), representing 7.4% and 6.3% of the global incidence, respectively. With respect to liver cancer mortality, China had the most deaths, with 172,068 deaths (95% UI, 139,621–212,496), accounting for 35.6% of global deaths. India ranked second with 34,534 deaths (95% UI, 30,718–38,880), accounting for 7.1%, and Japan had 31,123 deaths (95% UI, 26,459–33,773), accounting for 6.4%. The countries with the highest liver cancer burden were Mongolia (ASMR = 80.9, ASIR = 74.6, ASDR = 1993.2), Gambia (ASMR = 33.9, ASIR = 32.3, ASDR = 977.0), and Mali (ASMR = 29.3, ASIR = 27.4, ASDR = 795.8). Mongolia (76.6), the Republic of Korea (38.2), and Gambia (37.1) had the highest ASPRs. At the SDI level, middle-SDI countries presented the highest ASDRs and ASMRs, whereas high-SDI countries presented the highest ASIRs and ASPRs. Importantly, the ASIR (r = −0.1566, p < 0.01), ASMR (r = −0.2360, p < 0.001), and ASDR (r = -0.2996, p < 0.001) for liver cancer were negatively correlated with the SDI (Fig. 5E-H).


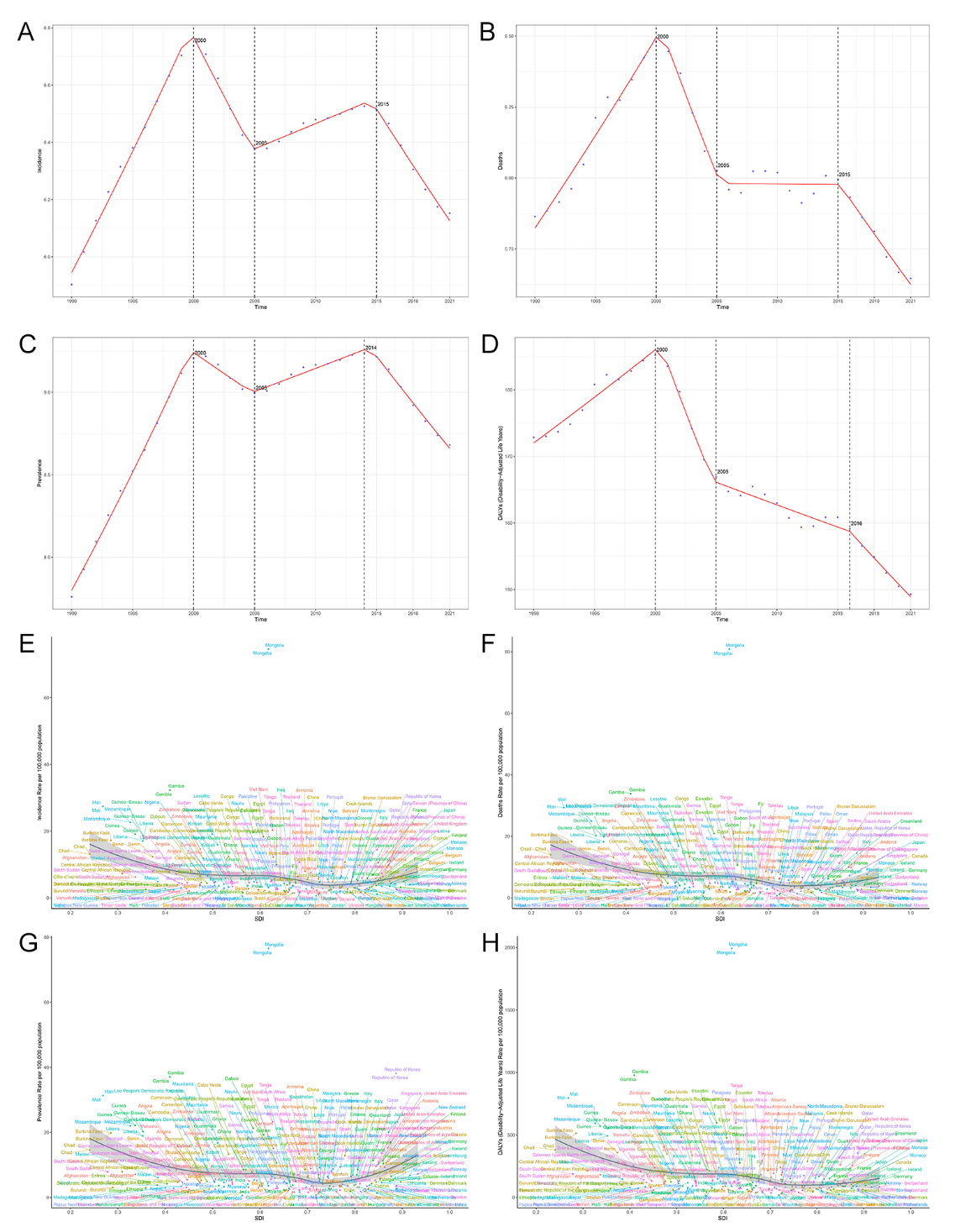


**Fig 5.** **A–D** Average annual percent changes in age-standardised global cancer incidence (**A**), mortality (**B**), prevalence (**C**), and disability-adjusted life years (DALYs) (**D**) in both sexes for liver cancer from 1990 to 2021. **E–H** Correlations of the sociodemographic index with age-standardised global cancer incidence €, mortality (**F**), prevalence (**G**), and DALYs (**H**) for liver cancer in 204 countries and territories.

**Pancreatic cancer**

In 2021, 508,533 (95% UI, 462,091–547,208) new cases of pancreatic cancer were reported worldwide, with 505,752 (95% UI, 461,224–543,899) deaths. The global ASMR for pancreatic cancer was 5.9 (5.4–6.4), and the ASIR was 6.0 (5.4–6.4). From 1990 to 2019, the ASIR (AAPC = 0.015, 0.013–0.016) and ASPR (AAPC = 0.023, 0.021–0.024) of pancreatic cancer gradually increased, reaching a peak around 2019 before starting to decline. The ASMR followed a similar trend, with a significant decline beginning in 2019 (AAPC = 0.009, 0.008–0.011). The ASDR increased sharply from 1990 to 1994 and then significantly declined after 1994, peaking between 1998 and 2019 before rapidly decreasing after 2019 (AAPC = 0.064, 0.033–0.094) (Fig. 6A-D). In terms of sex, there were differences in pancreatic cancer burden between males and females, with males having a higher ASMR (6.9 vs. 5.1), ASIR (7.0 vs. 5.0), ASPR (5.9 vs. 4.3), and ASDR (157.1 vs. 105.2). In terms of age distribution, the majority of cases and deaths occurred in individuals aged 50 years and older, accounting for 93.8% and 94.7% of the total, respectively. Among the 21 regions, High-income Asia Pacific had the highest ASIR and ASPR for pancreatic cancer, while Western Sub-Saharan Africa had the highest ASMR and ASDR. There are significant disparities in the burden of pancreatic cancer across different countries. The three countries with the highest pancreatic cancer incidence are China [118,665 (95% UI, 94,623–144,663)], the United States of America [61,340 (95% UI, 56,243–64,259)], and Japan [46,502 (95% UI, 39,057–50,709)], accounting for 23.3%, 12.1%, and 9.1% of the global incidence, respectively. In terms of mortality, China again has the highest number of deaths, totalling 119,602 (95% UI, 95,654–145,218), which represents 23.6% of global deaths. The United States of America follows with 57,098 deaths (95% UI, 52,182–59,928), accounting for 11.3%, and Japan ranks third with 42,065 deaths (95% UI, 35,527–45,867), accounting for 8.3% of global deaths. Greenland had the highest ASMR (15.9), ASIR (15.2), and ASDR (374.9), while Germany had the highest ASPR (14.1). In terms of the SDI, the ASIR, ASMR, ASPR, and ASDR for pancreatic cancer were highest in high-SDI countries and lowest in low-SDI countries, indicating a positive correlation with the SDI. Correlation analysis further confirmed that the ASIR (r = 0.7972, p < 0.001), ASMR (r = 0.7718, p < 0.001), ASPR (r = 0.8138, p < 0.001), and ASDR (r = 0.7467, p < 0.001) were significantly positively correlated with the SDI (Fig. 6E-H).


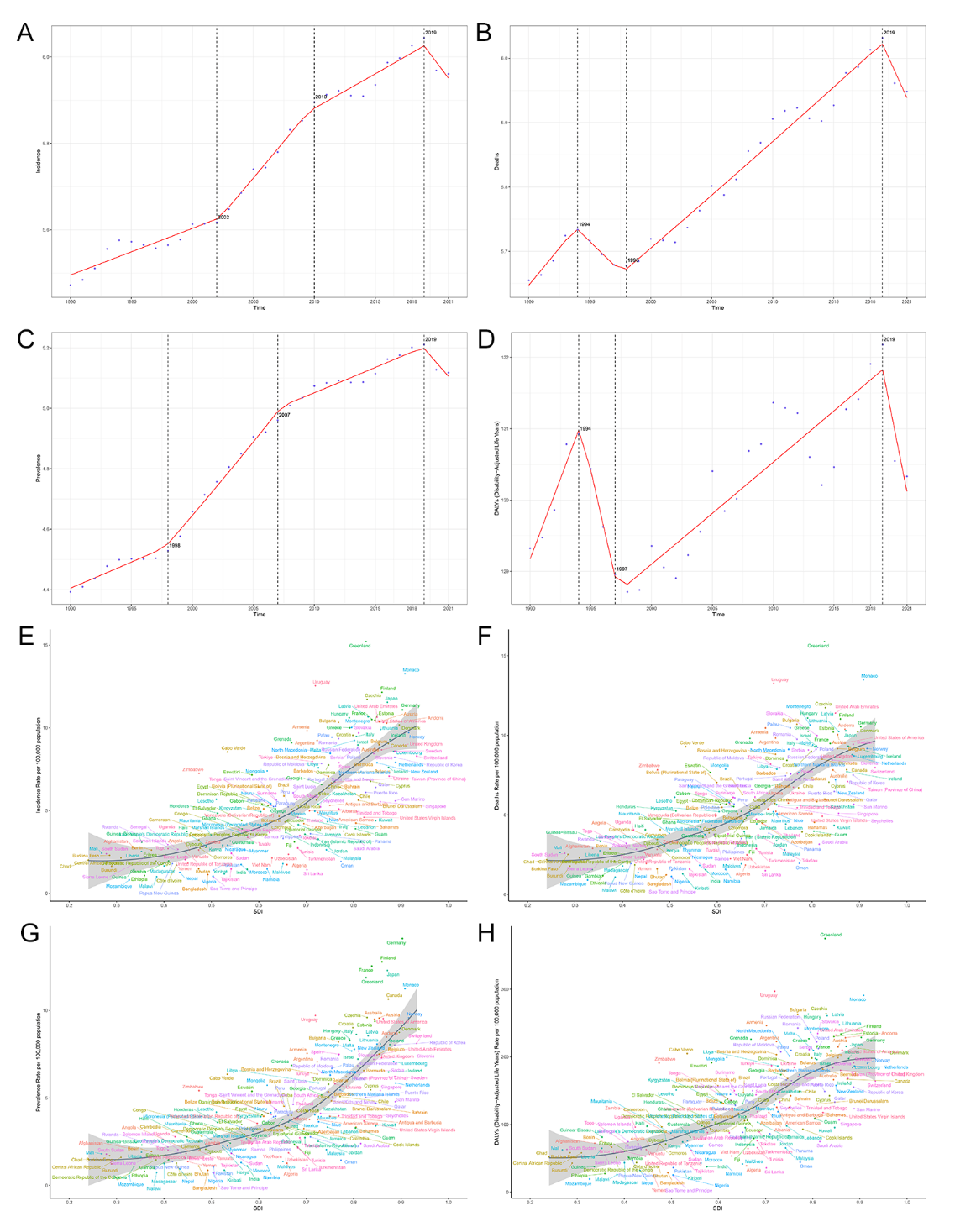


**Fig 6.** **A–D** Average annual percent changes in age-standardised global cancer incidence (**A**), mortality (**B**), prevalence (**C**), and disability-adjusted life years (DALYs) (**D**) in both sexes for pancreatic cancer from 1990 to 2021. **E–H** Correlations of the socio-demographic index with age-standardised global cancer incidence €, mortality (**F**), prevalence (**G**), and DALYs (**H**) for pancreatic cancer in 204 countries and territories.

**Prostate cancer**

In 2021, there were 1,324,383 new cases of prostate cancer worldwide (95% UI, 1,217,321–1,400,222) with 432,463 deaths (95% UI, 381,873–463,645). The global ASMR for prostate cancer was 5.3 (4.6–5.6), and the ASIR was 15.4 (14.1–16.3). From 1990 to 2008, the ASIR for prostate cancer gradually increased (AAPC = 0.057, 0.053–0.061), then rapidly declined after 2008 and continued to decline slowly from 2013 onwards. The ASPR (AAPC = 0.831, 0.789–0.872) showed a similar trend to that of the ASIR. The ASMR (AAPC = -0.033, -0.034–-0.031) and ASDR (AAPC = -0.558, -0.575–-0.540) exhibited similar trends, with notable declines starting in 1995 (Fig. 7A-D). As a male-specific cancer, an ASMR of 12.6, ASIR of 34.1, ASPR of 260.1, and ASDR of 217.8 were observed. In terms of age distribution, the number of prostate cancer cases [734,673 (95% UI, 669,126–779,148)] and deaths [338,640 (95% UI, 298,294–362,941)] was highest among individuals aged 70 years and above, accounting for more than half of the total, with proportions of 55.5% and 78.3%, respectively. Among the 21 regions, High-income North America, Australasia, and the Caribbean had the highest ASIRs for prostate cancer. The highest ASPR was observed in Australasia, followed by High-income North America and the Caribbean. Western Sub-Saharan Africa had the highest ASMR, and the Caribbean had the highest ASDR. Among the 204 countries, the three countries with the highest prostate cancer incidence are the United States of America [297,836 (95% UI, 280,073–311,400)], China [88,601 (95% UI, 63,194–120,965)], and Germany [75,107 (95% UI, 63,900–87,456)], accounting for 22.5%, 6.7%, and 5.7% of the global incidence, respectively. In terms of mortality, the United States of America again has the highest number of deaths, totalling 44,032 (95% UI, 39,406–46,728), representing 10.2% of global deaths, followed by China, with 37,363 deaths (95% UI, 27,851–50,366), accounting for 8.6%, and India, with 24,995 deaths (95% UI, 20,000–33,898), accounting for 5.8%. The highest ASMRs for prostate cancer were found in Saint Vincent and the Grenadines (37.1), Saint Kitts and Nevis (34.7), and Antigua and Barbuda (31.9). Bermuda had the highest ASIR (85.8) and ASPR (680.3), while Saint Vincent and the Grenadines (634.3) had the highest ASDR, followed by Saint Kitts and Nevis (610.7) and Grenada (558.1). According to the SDI levels, countries with a high SDI presented the highest ASIRs and ASPR, whereas countries with a low SDI presented the highest ASMRs and ASDRs. Furthermore, a significant positive correlation was observed between the SDI and the ASIR (r = 0.5576, p < 0.001) and ASPR (r = 0.7130, p < 0.001) for prostate cancer, whereas no significant correlation was detected for the ASMR (r = -0.1181, p = 0.09) and ASDR (r = -0.1084, p = 0.12) with the SDI (Fig. 7E-H).


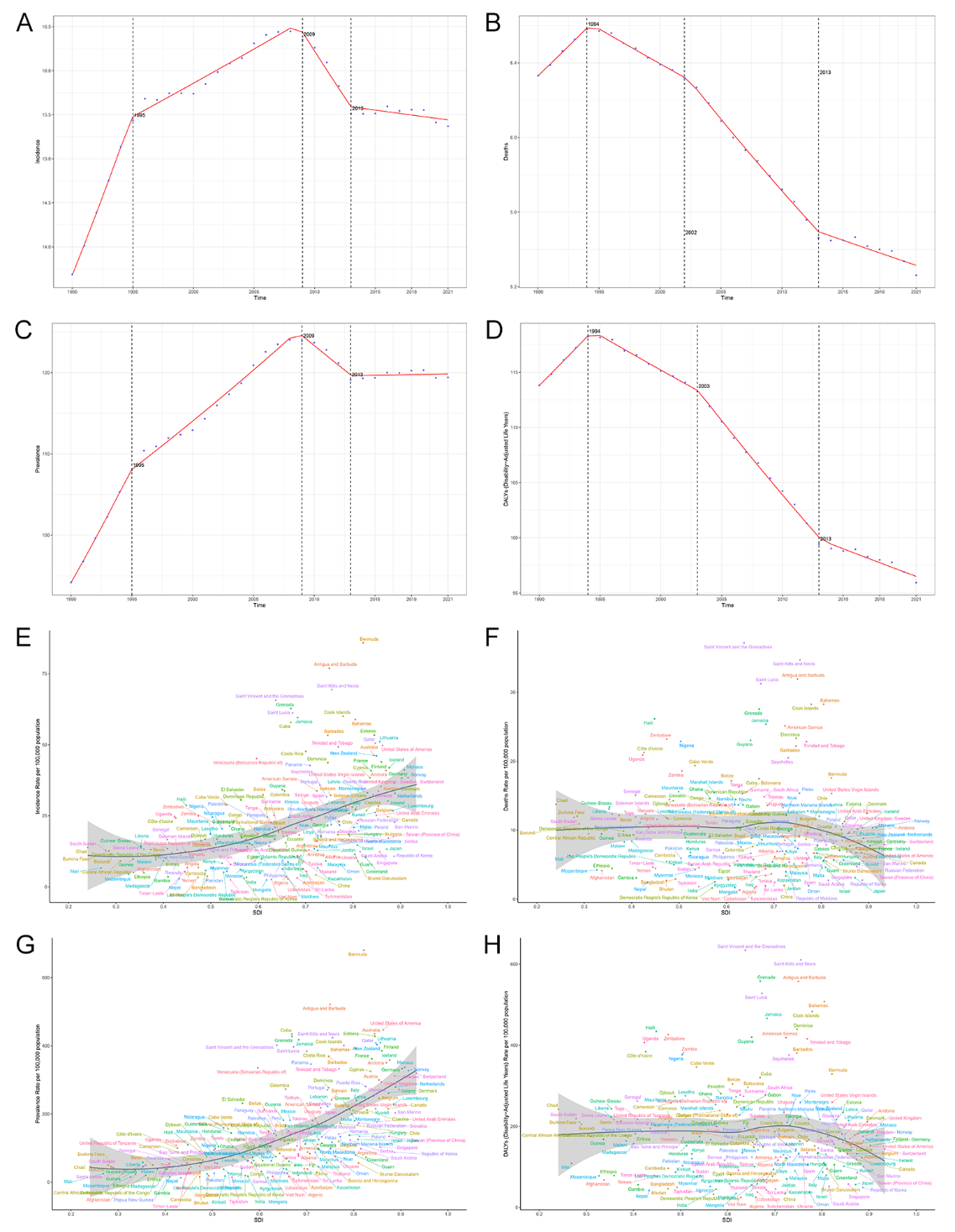


**Fig 7.** **A–D** Average annual percent changes in age-standardised global cancer incidence (**A**), mortality (**B**), prevalence (**C**), and disability-adjusted life years (DALYs) (**D**) in both sexes for prostate cancer from 1990 to 2021. **E–H** Correlations of the sociodemographic index with age-standardised global cancer incidence €, mortality (**F**), prevalence (**G**), and DALYs (**H**) for prostate cancer in 204 countries and territories.

**Thyroid cancer**

The global number of thyroid cancer cases in 2021 was 249,538 (95% UI, 223,290–274,638), with 44,799 deaths (95% UI, 39,925–48,541). The global ASMR for thyroid cancer was 0.53 (0.47–0.58), and the ASIR was 2.9 (2.6–3.2). From 1990 to 2021, the ASIR (AAPC=0.027, 95% CI 0.026–0.028) showed a consistent upwards trend. Similarly, the ASPR (AAPC=0.265, 95% CI 0.260–0.269) mirrored the trend in the ASIR. In contrast, the ASMR (AAPC=-0.001, 95% CI -0.001–-0.001) declined significantly starting in 1995, rose slightly after 2004, and decreased rapidly after 2013. The ASDR (AAPC=-0.020, 95% CI -0.023–-0.017) also decreased significantly from 1995, increased rapidly after 2004, and declined sharply after 2010 (Fig. 8A-D). With respect to sex differences, females had higher rates than males across all the metrics: ASMR (0.58 vs. 0.47), ASIR (3.8 vs. 2.0), ASPR (31.3 vs. 14.8), and ASDR (16.7 vs. 12.3). In terms of age distribution, individuals aged 50–69 years had the highest incidence of thyroid cancer (107,986 cases, 95% UI, 97,139–117,295), accounting for 43.2% of the total cases. Meanwhile, those aged 70 years and older had the highest number of deaths (22,575 deaths, 95% UI, 19,675–24,449), representing 50.3% of the total deaths. Among the 21 global regions, High-income North America reported the highest ASIR and ASPR for thyroid cancer. Andean Latin America had the highest ASMR, whereas Eastern Sub-Saharan Africa and Andean Latin America presented the highest ASDRs. At the country level, the three countries with the highest incidence and mortality of thyroid cancer are China [incidence: 48,104 (95% UI, 38,695–60,068); death: 7,692 (95% UI, 6,123–9,429)], India [incidence: 28,334 (95% UI, 22,851–34,107); death: 7,156 (95% UI, 5,976–8,237)], and the United States of America [incidence: 26,396 (95% UI, 25,052–27,472); death: 2,495 (95% UI, 2,237–2,641)]. These countries account for 19.3%, 11.4%, and 10.6% of the global thyroid cancer incidence and 17.2%, 16.0%, and 5.6% of the global thyroid cancer mortality, respectively. Ethiopia had the highest ASMR (1.6) and ASDR (44.2), whereas Saudi Arabia recorded the highest ASIR (7.1) and ASPR (60.1). The ASIRs and ASPRs for thyroid cancer were highest in countries with high SDI levels, whereas countries with low SDI levels had the lowest rates for both indicators. Conversely, the ASMRs and ASDRs were highest in low-SDI countries and lowest in high-SDI countries. Correlation analyses confirmed significant positive associations of the ASIR (r=0.5929, p<0.001) and ASPR (r=0.6592, p<0.001) with the SDI, whereas no significant correlations were detected for the ASMR (r=-0.0704, p=0.31) and ASDR (r=-0.1023, p=0.15) (Fig. 8E-H).


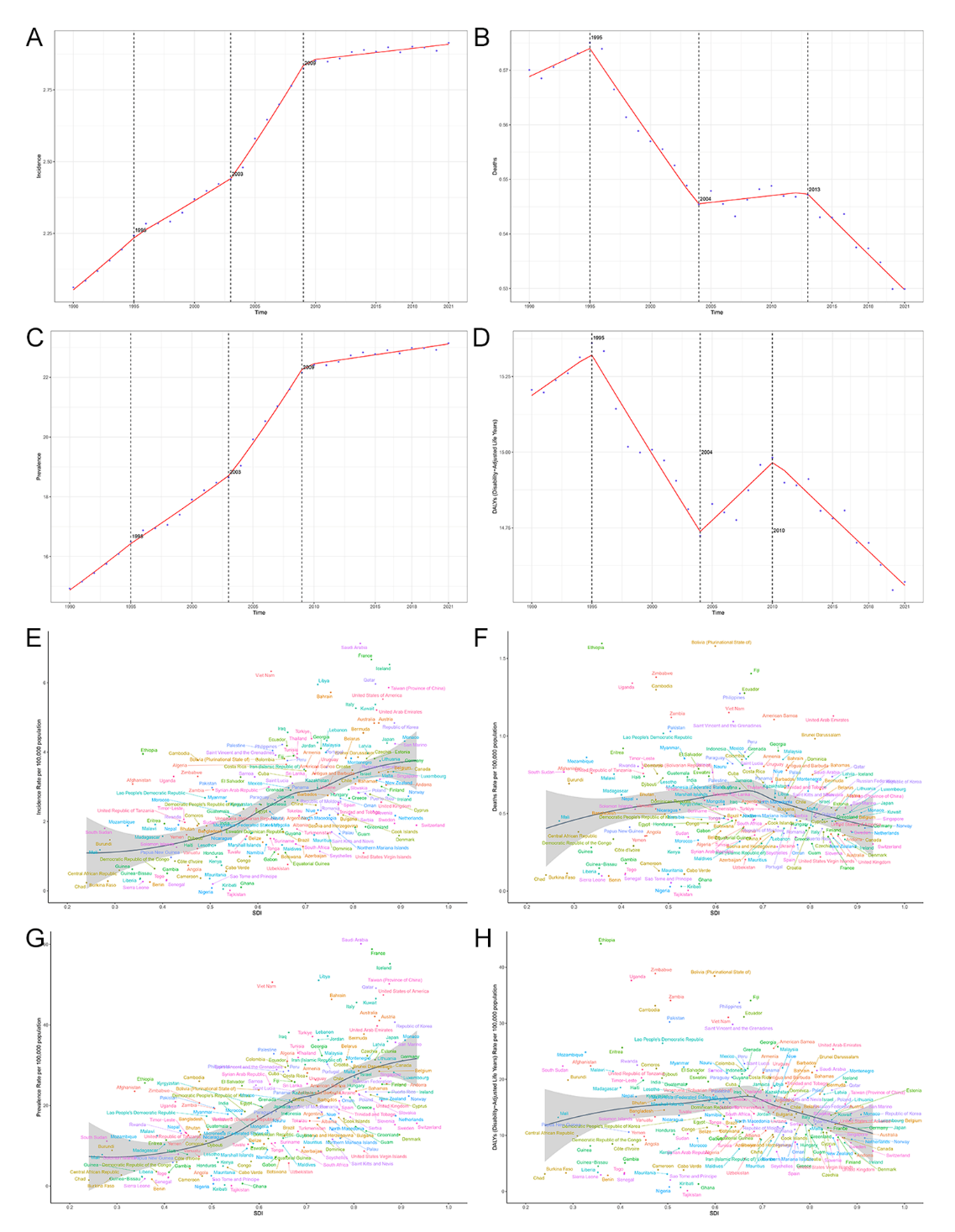


**Fig 8.** **A–D** Average annual percent changes in age-standardised global cancer incidence (**A**), mortality (**B**), prevalence (**C**), and disability-adjusted life years (DALYs) (**D**) in both sexes for thyroid cancer from 1990 to 2021. **E–H** Correlations of the sociodemographic index with age-standardised global cancer incidence €, mortality (**F**), prevalence (**G**), and DALYs (**H**) for thyroid cancer in 204 countries and territories.

**Table S1** Global incidence and deaths in 2021 for total cancers and 34 cancer groups by gender

| **Cancer type** | **2021** | | | | | | | | | | | | | | | | | | | | | | | |
| --- | --- | --- | --- | --- | --- | --- | --- | --- | --- | --- | --- | --- | --- | --- | --- | --- | --- | --- | --- | --- | --- | --- | --- | --- |
|  | **Death** | | | | | | | | | | | | **Incidence** | | | | | | | | | | | |
|  | **Number (95% UI)** | | | | | | **Age-standardized per 100,000 (95% UI)** | | | | | | **Number (95% UI)** | | | | | | **Age-standardized per 100,000 (95% UI)** | | | | | |
|  | **Female** | | | **Male** | | | **Female** | | | **Male** | | | **Female** | | | **Male** | | | **Female** | | | **Male** | | |
|  | **Value** | **Upper** | **Lower** | **Value** | **Upper** | **Lower** | **Value** | **Upper** | **Lower** | **Value** | **Upper** | **Lower** | **Value** | **Upper** | **Lower** | **Value** | **Upper** | **Lower** | **Value** | **Upper** | **Lower** | **Value** | **Upper** | **Lower** |
| **Total cancer** | 4262974.8 | 4598444.1 | 3866365.7 | 5570259.6 | 6096397.6 | 5140018.7 | 93.1 | 100.4 | 84.6 | 144.8 | 157.8 | 133.7 | 10768662.8 | 11439753.4 | 10044116.5 | 12797582.6 | 13551812.9 | 12019330.8 | 237.4 | 252.2 | 221.8 | 325.3 | 343.9 | 306.1 |
| **Total cancer without NMSC** | 4238249.5 | 4570437.2 | 3844404.2 | 5538071.7 | 6062221.0 | 5109237.0 | 92.6 | 99.8 | 84.1 | 143.8 | 156.9 | 132.9 | 8128134.3 | 8746214.8 | 7473431.0 | 9101265.0 | 9883388.3 | 8460283.2 | 180.1 | 193.7 | 165.9 | 229.5 | 248.6 | 213.6 |
| **Bladder cancer** | 57264.4 | 62932.9 | 49416.7 | 164623.9 | 182251.9 | 149887.2 | 1.2 | 1.3 | 1.1 | 4.7 | 5.2 | 4.2 | 122603.8 | 133928.4 | 108167.2 | 417706.0 | 456194.5 | 384651.5 | 2.6 | 2.9 | 2.3 | 10.9 | 11.9 | 10.0 |
| **Brain and central nervous system cancer** | 115722.6 | 128972.0 | 102256.3 | 142904.2 | 174945.2 | 110661.8 | 2.6 | 2.9 | 2.3 | 3.5 | 4.3 | 2.7 | 167352.7 | 187695.4 | 147253.1 | 190129.6 | 232469.1 | 148117.8 | 3.9 | 4.3 | 3.4 | 4.7 | 5.8 | 3.7 |
| **Breast cancer** | 660925.3 | 707181.9 | 609171.3 | 13274.1 | 16240.1 | 9074.3 | 14.5 | 15.6 | 13.5 | 0.3 | 0.4 | 0.2 | 2082737.0 | 2225082.6 | 1940351.2 | 38827.3 | 47845.9 | 24650.5 | 46.4 | 49.6 | 43.3 | 0.9 | 1.2 | 0.6 |
| **Cervical cancer** | 296667.2 | 321905.7 | 272058.6 | 0.0 | 0.0 | 0.0 | 6.6 | 7.2 | 6.1 | 0.0 | 0.0 | 0.0 | 667426.4 | 726422.1 | 613030.1 | 0.0 | 0.0 | 0.0 | 15.3 | 16.7 | 14.1 | 0.0 | 0.0 | 0.0 |
| **Colon and rectum cancer** | 462514.9 | 503539.2 | 407296.3 | 581557.3 | 641420.1 | 528252.8 | 10.0 | 10.8 | 8.8 | 15.3 | 16.9 | 13.9 | 930680.9 | 1017652.4 | 824673.7 | 1263462.4 | 1400377.2 | 1146499.5 | 20.2 | 22.0 | 17.9 | 31.9 | 35.3 | 29.0 |
| **Esophageal cancer** | 138805.5 | 161288.1 | 107414.0 | 399796.4 | 459870.9 | 343473.3 | 3.0 | 3.5 | 2.3 | 10.1 | 11.6 | 8.7 | 148142.2 | 172538.1 | 113641.4 | 428387.1 | 495196.3 | 367887.9 | 3.2 | 3.7 | 2.5 | 10.6 | 12.2 | 9.2 |
| **Eye cancer** | 5159.7 | 6533.4 | 3903.7 | 5273.1 | 7162.6 | 3584.2 | 0.1 | 0.2 | 0.1 | 0.1 | 0.2 | 0.1 | 16720.8 | 20519.8 | 13040.6 | 17230.0 | 22466.0 | 12411.3 | 0.4 | 0.5 | 0.3 | 0.4 | 0.6 | 0.3 |
| **Gallbladder and biliary tract cancer** | 96014.1 | 111372.4 | 77016.0 | 75947.1 | 88998.8 | 57121.9 | 2.1 | 2.4 | 1.7 | 2.0 | 2.4 | 1.5 | 115984.8 | 132850.2 | 94592.8 | 100783.6 | 116395.6 | 77371.7 | 2.5 | 2.9 | 2.0 | 2.6 | 3.0 | 2.1 |
| **Hodgkin lymphoma** | 11071.2 | 13489.2 | 7725.3 | 17108.8 | 23535.1 | 11820.4 | 0.3 | 0.3 | 0.2 | 0.4 | 0.6 | 0.3 | 26563.1 | 30708.6 | 20832.6 | 38619.0 | 48873.8 | 29778.2 | 0.6 | 0.7 | 0.5 | 1.0 | 1.2 | 0.7 |
| **Kidney cancer** | 54650.9 | 58643.2 | 48631.5 | 106543.7 | 112786.7 | 99856.6 | 1.2 | 1.3 | 1.1 | 2.8 | 3.0 | 2.6 | 135239.5 | 144234.8 | 124078.9 | 252589.2 | 268143.5 | 237746.5 | 3.0 | 3.2 | 2.8 | 6.3 | 6.6 | 5.9 |
| **Larynx cancer** | 16858.7 | 19875.5 | 14208.6 | 100392.9 | 108829.6 | 93350.8 | 0.4 | 0.4 | 0.3 | 2.5 | 2.7 | 2.3 | 29094.4 | 33679.9 | 24974.6 | 171788.6 | 186042.2 | 159470.2 | 0.6 | 0.7 | 0.5 | 4.2 | 4.5 | 3.9 |
| **Leukemia** | 138701.2 | 152783.1 | 115854.3 | 181582.4 | 212912.5 | 146802.3 | 3.2 | 3.5 | 2.6 | 4.8 | 5.6 | 3.9 | 197710.3 | 217864.8 | 164767.4 | 263712.4 | 302340.4 | 211845.1 | 4.6 | 5.1 | 3.8 | 6.8 | 7.8 | 5.5 |
| **Lip and oral cavity cancer** | 71489.0 | 78442.6 | 64699.2 | 136890.5 | 149372.0 | 120655.8 | 1.6 | 1.7 | 1.4 | 3.4 | 3.7 | 3.0 | 148660.1 | 160404.3 | 135703.8 | 272917.0 | 296015.5 | 245321.4 | 3.3 | 3.5 | 3.0 | 6.7 | 7.2 | 6.0 |
| **Liver cancer** | 159178.7 | 175020.5 | 142936.4 | 324696.4 | 376834.1 | 288483.4 | 3.5 | 3.8 | 3.1 | 8.1 | 9.4 | 7.2 | 164847.9 | 181387.6 | 147932.3 | 364354.5 | 422726.9 | 321977.3 | 3.6 | 4.0 | 3.2 | 9.0 | 10.4 | 8.0 |
| **Malignant neoplasm of bone and articular cartilage** | 27008.0 | 30879.9 | 22514.8 | 39106.3 | 45671.7 | 29362.7 | 0.6 | 0.7 | 0.5 | 1.0 | 1.1 | 0.7 | 36681.8 | 42202.0 | 30677.1 | 54693.3 | 63656.8 | 40900.0 | 0.9 | 1.0 | 0.7 | 1.4 | 1.6 | 1.0 |
| **Malignant skin melanoma** | 27227.7 | 30950.4 | 23212.2 | 34322.0 | 37624.8 | 30494.4 | 0.6 | 0.7 | 0.5 | 0.9 | 1.0 | 0.8 | 141788.7 | 153060.0 | 130334.8 | 161315.9 | 171042.7 | 150889.9 | 3.2 | 3.4 | 2.9 | 4.1 | 4.4 | 3.8 |
| **Mesothelioma** | 8040.2 | 8864.6 | 7130.4 | 21578.7 | 23242.7 | 20028.9 | 0.2 | 0.2 | 0.2 | 0.6 | 0.6 | 0.5 | 8723.7 | 9580.0 | 7784.4 | 23184.2 | 24941.2 | 21553.4 | 0.2 | 0.2 | 0.2 | 0.6 | 0.6 | 0.6 |
| **Multiple myeloma** | 53237.9 | 60880.1 | 44829.7 | 63121.7 | 70180.1 | 54440.3 | 1.1 | 1.3 | 1.0 | 1.7 | 1.9 | 1.4 | 66300.6 | 75286.9 | 56020.5 | 82454.0 | 90736.4 | 71457.3 | 1.4 | 1.6 | 1.2 | 2.1 | 2.3 | 1.8 |
| **Nasopharynx cancer** | 21421.8 | 24202.8 | 19065.5 | 53936.8 | 61333.3 | 47076.1 | 0.5 | 0.5 | 0.4 | 1.3 | 1.5 | 1.1 | 32394.9 | 38077.0 | 27948.0 | 86483.0 | 101788.5 | 73982.7 | 0.7 | 0.9 | 0.6 | 2.1 | 2.4 | 1.8 |
| **Neuroblastoma and other peripheral nervous cell tumors** | 2213.1 | 2628.4 | 1740.9 | 2980.7 | 3469.5 | 2476.7 | 0.1 | 0.1 | 0.0 | 0.1 | 0.1 | 0.1 | 4574.4 | 5972.0 | 3344.4 | 6293.0 | 8207.8 | 4741.6 | 0.1 | 0.2 | 0.1 | 0.2 | 0.2 | 0.1 |
| **Non-Hodgkin lymphoma** | 113314.3 | 122998.5 | 100810.6 | 153746.9 | 170903.0 | 141088.8 | 2.5 | 2.7 | 2.2 | 4.0 | 4.4 | 3.7 | 245412.5 | 264809.6 | 221528.9 | 359141.7 | 389787.9 | 327659.8 | 5.5 | 5.9 | 4.9 | 9.0 | 9.8 | 8.2 |
| **Non-melanoma skin cancer** | 24725.3 | 27617.3 | 20539.1 | 32187.9 | 36792.1 | 26973.4 | 0.5 | 0.6 | 0.4 | 0.9 | 1.0 | 0.8 | 2640528.5 | 2873576.2 | 2382229.2 | 3696317.6 | 4031870.8 | 3357251.2 | 57.3 | 62.4 | 51.7 | 95.8 | 103.9 | 87.2 |
| **Other malignant neoplasms** | 110133.2 | 123142.2 | 97575.3 | 112075.2 | 125906.6 | 93709.4 | 2.4 | 2.7 | 2.2 | 2.9 | 3.3 | 2.4 | 211225.8 | 233643.0 | 186889.1 | 211069.2 | 237470.5 | 180120.4 | 4.7 | 5.3 | 4.2 | 5.3 | 6.0 | 4.6 |
| **Other neoplasms** | 24018.2 | 26682.5 | 20082.9 | 31160.9 | 40124.6 | 26225.7 | 0.5 | 0.6 | 0.4 | 0.9 | 1.2 | 0.8 | 28647875.8 | 34751251.7 | 23209272.4 | 14265486.1 | 16910442.7 | 11786842.1 | 686.0 | 830.5 | 556.9 | 347.8 | 410.5 | 288.1 |
| **Other pharynx cancer** | 17998.2 | 22431.0 | 15451.8 | 80436.9 | 87130.8 | 73958.5 | 0.4 | 0.5 | 0.3 | 1.9 | 2.1 | 1.8 | 32753.4 | 39596.6 | 28866.6 | 137066.1 | 146457.8 | 128147.9 | 0.7 | 0.9 | 0.6 | 3.3 | 3.5 | 3.0 |
| **Ovarian cancer** | 185608.7 | 201012.7 | 167962.0 | 0.0 | 0.0 | 0.0 | 4.1 | 4.4 | 3.7 | 0.0 | 0.0 | 0.0 | 298876.0 | 324501.0 | 270729.8 | 0.0 | 0.0 | 0.0 | 6.7 | 7.3 | 6.1 | 0.0 | 0.0 | 0.0 |
| **Pancreatic cancer** | 235714.7 | 256636.8 | 206198.6 | 270037.4 | 295172.9 | 247469.9 | 5.1 | 5.5 | 4.4 | 6.9 | 7.5 | 6.4 | 234915.6 | 255434.6 | 205148.7 | 273617.1 | 299347.6 | 250808.5 | 5.0 | 5.5 | 4.4 | 7.0 | 7.6 | 6.4 |
| **Prostate cancer** | 0.0 | 0.0 | 0.0 | 432463.3 | 463645.3 | 381872.8 | 0.0 | 0.0 | 0.0 | 12.6 | 13.5 | 11.2 | 0.0 | 0.0 | 0.0 | 1324382.9 | 1400222.2 | 1217320.9 | 0.0 | 0.0 | 0.0 | 34.1 | 36.0 | 31.3 |
| **Soft tissue and other extraosseous sarcomas** | 23040.2 | 27497.8 | 19936.9 | 27162.9 | 36530.3 | 22113.5 | 0.5 | 0.6 | 0.5 | 0.7 | 0.9 | 0.6 | 43853.0 | 51362.0 | 38483.2 | 52347.9 | 68477.9 | 43223.1 | 1.0 | 1.2 | 0.9 | 1.3 | 1.7 | 1.1 |
| **Stomach cancer** | 329822.2 | 368392.8 | 287083.4 | 624551.4 | 750101.3 | 514019.5 | 7.1 | 8.0 | 6.2 | 16.0 | 19.1 | 13.2 | 397311.7 | 446652.9 | 344271.7 | 832920.9 | 1006469.3 | 687723.1 | 8.6 | 9.7 | 7.5 | 20.9 | 25.2 | 17.2 |
| **Testicular cancer** | 0.0 | 0.0 | 0.0 | 11388.3 | 12055.6 | 10770.7 | 0.0 | 0.0 | 0.0 | 0.3 | 0.3 | 0.3 | 0.0 | 0.0 | 0.0 | 91507.4 | 95709.7 | 87965.9 | 0.0 | 0.0 | 0.0 | 2.2 | 2.3 | 2.2 |
| **Thyroid cancer** | 26767.6 | 30904.5 | 23015.1 | 18030.9 | 19895.3 | 15238.3 | 0.6 | 0.7 | 0.5 | 0.5 | 0.5 | 0.4 | 167236.6 | 195646.6 | 147083.1 | 82301.4 | 91093.0 | 71574.6 | 3.8 | 4.5 | 3.4 | 2.0 | 2.2 | 1.7 |
| **Tracheal, bronchus, and lung cancer** | 674006.0 | 747508.3 | 595385.7 | 1342541.4 | 1513563.1 | 1180488.7 | 14.5 | 16.1 | 12.8 | 34.3 | 38.6 | 30.3 | 778707.7 | 862190.0 | 689762.0 | 1501980.5 | 1697520.6 | 1317171.5 | 16.8 | 18.6 | 14.9 | 37.8 | 42.7 | 33.3 |
| **Uterine cancer** | 97672.1 | 108061.5 | 86515.8 | 0.0 | 0.0 | 0.0 | 2.1 | 2.3 | 1.9 | 0.0 | 0.0 | 0.0 | 473613.9 | 513666.9 | 429915.6 | 0.0 | 0.0 | 0.0 | 10.4 | 11.2 | 9.4 | 0.0 | 0.0 | 0.0 |


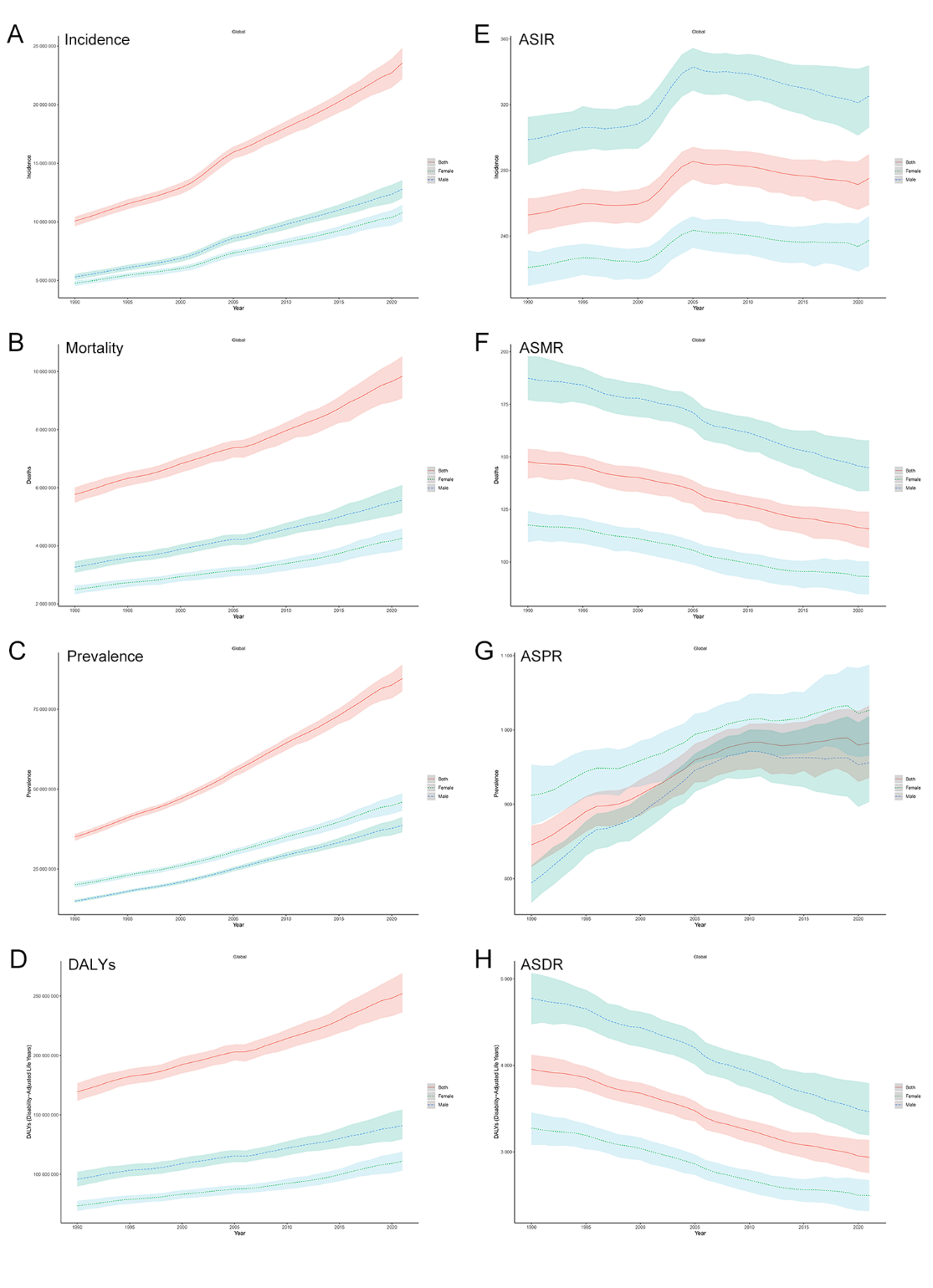


**Figure S1.** **A-D** Trends in global cancer incidence, mortality, prevalence, and disability-adjusted life years (DALYs) from 1990 to 2021, by sex. **E-H** Trends in global cancer ASIR, ASMR, ASPR, and ASDR from 1990 to 2021, by sex. Abbreviation: ASIR age-standardized incidence rate, ASMR age-standardized mortality rate, ASPR, age-standardized prevalence rate, ASDR, age-standardized DALYs rate.

**Table S2** Age-standardised global cancer incidence (ASIR), mortality (ASMR), prevalence (ASPR), and disability-adjusted life years (ASDR) for 34 specific cancer types in 2021, by sex

| **Cancer type** | **ASMR** | | **ASDR** | | **ASIR** | | **ASPR** | |
| --- | --- | --- | --- | --- | --- | --- | --- | --- |
|  | **Male** | **Female** | **Male** | **Female** | **Male** | **Female** | **Male** | **Female** |
| **Bladder cancer** | 4.7 | 1.2 | 86.3 | 23.3 | 10.9 | 2.6 | 58.9 | 14.3 |
| **Brain and central nervous system cancer** | 3.5 | 2.6 | 123.0 | 93.5 | 4.7 | 3.9 | 12.3 | 11.8 |
| **Breast cancer** | 0.3 | 14.5 | 9.2 | 455.6 | 0.9 | 46.4 | 7.8 | 450.6 |
| **Cervical cancer** | 0.0 | 6.6 | 0.0 | 226.3 | 0.0 | 15.3 | 0.0 | 79.3 |
| **Colon and rectum cancer** | 15.3 | 10.0 | 349.7 | 224.3 | 31.9 | 20.2 | 166.1 | 107.1 |
| **Esophageal cancer** | 10.1 | 3.0 | 237.8 | 67.8 | 10.6 | 3.2 | 17.7 | 5.8 |
| **Eye cancer** | 0.1 | 0.1 | 6.4 | 6.2 | 0.4 | 0.4 | 3.6 | 3.4 |
| **Gallbladder and biliary tract cancer** | 2.0 | 2.1 | 42.0 | 44.6 | 2.6 | 2.5 | 4.0 | 3.4 |
| **Hodgkin lymphoma** | 0.4 | 0.3 | 18.3 | 11.4 | 1.0 | 0.6 | 5.1 | 3.7 |
| **Kidney cancer** | 2.8 | 1.2 | 67.8 | 28.9 | 6.3 | 3.0 | 30.3 | 15.7 |
| **Larynx cancer** | 2.5 | 0.4 | 64.1 | 9.9 | 4.2 | 0.6 | 22.5 | 3.6 |
| **Leukemia** | 4.8 | 3.2 | 160.4 | 115.3 | 6.8 | 4.6 | 25.2 | 17.3 |
| **Lip and oral cavity cancer** | 3.4 | 1.6 | 94.5 | 42.6 | 6.7 | 3.3 | 23.1 | 12.7 |
| **Liver cancer** | 8.1 | 3.5 | 217.7 | 85.1 | 9.0 | 3.6 | 12.8 | 5.0 |
| **Malignant neoplasm of bone and articular cartilage** | 1.0 | 0.6 | 38.0 | 24.3 | 1.4 | 0.9 | 8.9 | 5.7 |
| **Malignant skin melanoma** | 0.9 | 0.6 | 23.2 | 16.6 | 4.1 | 3.2 | 27.8 | 23.6 |
| **Mesothelioma** | 0.6 | 0.2 | 12.2 | 4.4 | 0.6 | 0.2 | 1.0 | 0.3 |
| **Multiple myeloma** | 1.7 | 1.1 | 35.8 | 25.0 | 2.1 | 1.4 | 5.5 | 3.7 |
| **Nasopharynx cancer** | 1.3 | 0.5 | 42.3 | 16.1 | 2.1 | 0.7 | 9.1 | 3.2 |
| **Neuroblastoma and other peripheral nervous cell tumors** | 0.1 | 0.1 | 4.5 | 3.4 | 0.2 | 0.1 | 0.9 | 0.7 |
| **Non-Hodgkin lymphoma** | 4.0 | 2.5 | 116.3 | 71.4 | 9.0 | 5.5 | 43.5 | 25.7 |
| **Non-melanoma skin cancer** | 0.9 | 0.5 | 18.6 | 10.8 | 95.8 | 57.3 | 45.0 | 22.9 |
| **Other malignant neoplasms** | 2.9 | 2.4 | 84.1 | 72.4 | 5.3 | 4.7 | 22.0 | 20.6 |
| **Other neoplasms** | 0.9 | 0.5 | 18.8 | 11.3 | 347.8 | 686.0 | 610.7 | 1197.0 |
| **Other pharynx cancer** | 1.9 | 0.4 | 54.8 | 11.4 | 3.3 | 0.7 | 6.0 | 1.5 |
| **Ovarian cancer** | 0.0 | 4.1 | 0.0 | 115.1 | 0.0 | 6.7 | 0.0 | 28.1 |
| **Pancreatic cancer** | 6.9 | 5.1 | 157.1 | 105.2 | 7.0 | 5.0 | 5.9 | 4.3 |
| **Prostate cancer** | 12.6 | 0.0 | 217.8 | 0.0 | 34.1 | 0.0 | 260.1 | 0.0 |
| **Soft tissue and other extraosseous sarcomas** | 0.7 | 0.5 | 23.0 | 18.3 | 1.3 | 1.0 | 6.5 | 5.2 |
| **Stomach cancer** | 16.0 | 7.1 | 371.2 | 165.6 | 20.9 | 8.6 | 41.4 | 15.2 |
| **Testicular cancer** | 0.3 | 0.0 | 13.8 | 0.0 | 2.2 | 0.0 | 16.6 | 0.0 |
| **Thyroid cancer** | 0.5 | 0.6 | 12.3 | 16.7 | 2.0 | 3.8 | 14.8 | 31.3 |
| **Tracheal, bronchus, and lung cancer** | 34.3 | 14.5 | 763.7 | 328.8 | 37.8 | 16.8 | 51.5 | 24.9 |
| **Uterine cancer** | 0.0 | 2.1 | 0.0 | 56.1 | 0.0 | 10.4 | 0.0 | 75.7 |


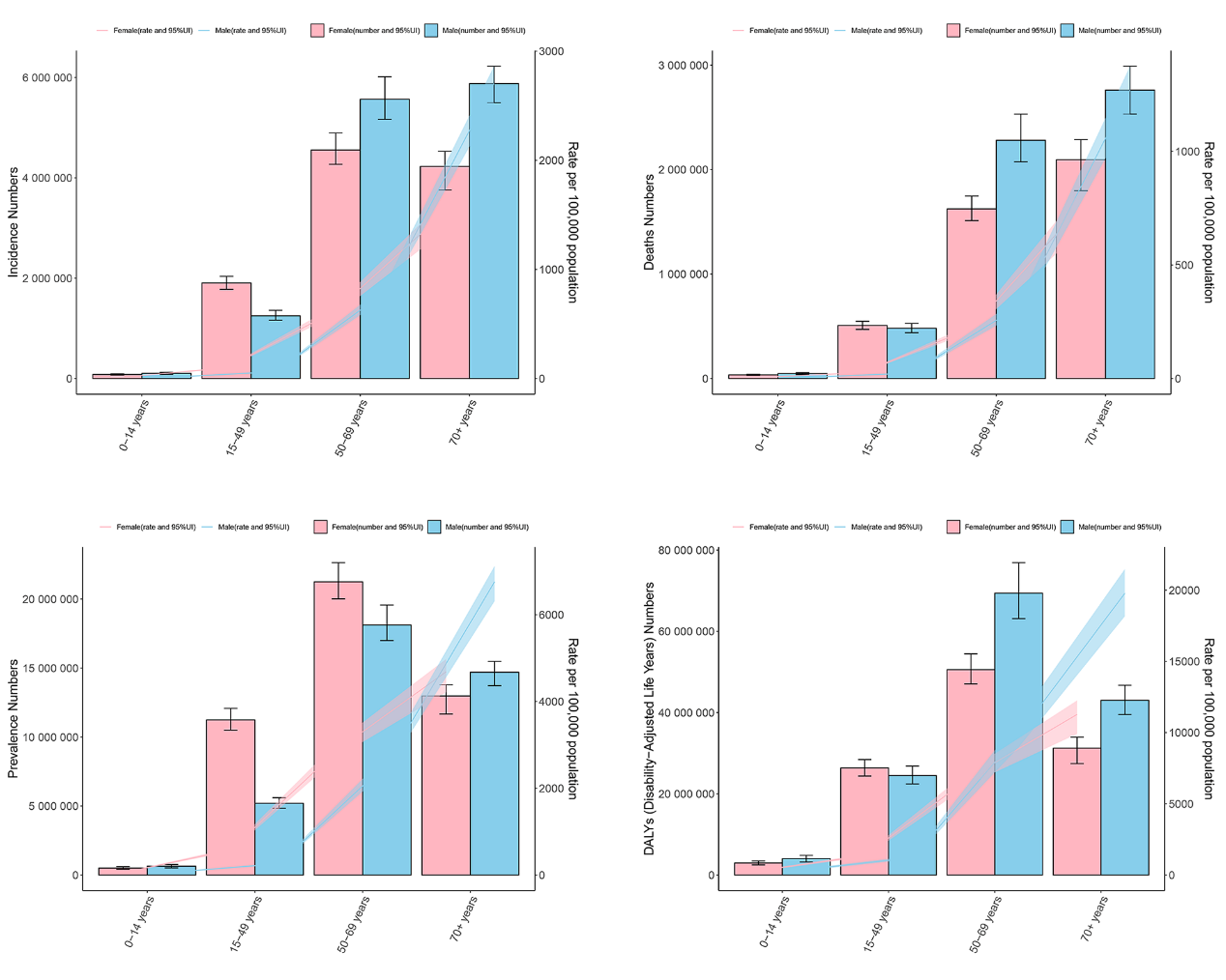


**Figure S2.** Global cancer incidence, mortality, prevalence, and disability-adjusted life years (DALYs), along with age-standardized incidence, mortality, prevalence, and DALYs by age group and sex in 2021


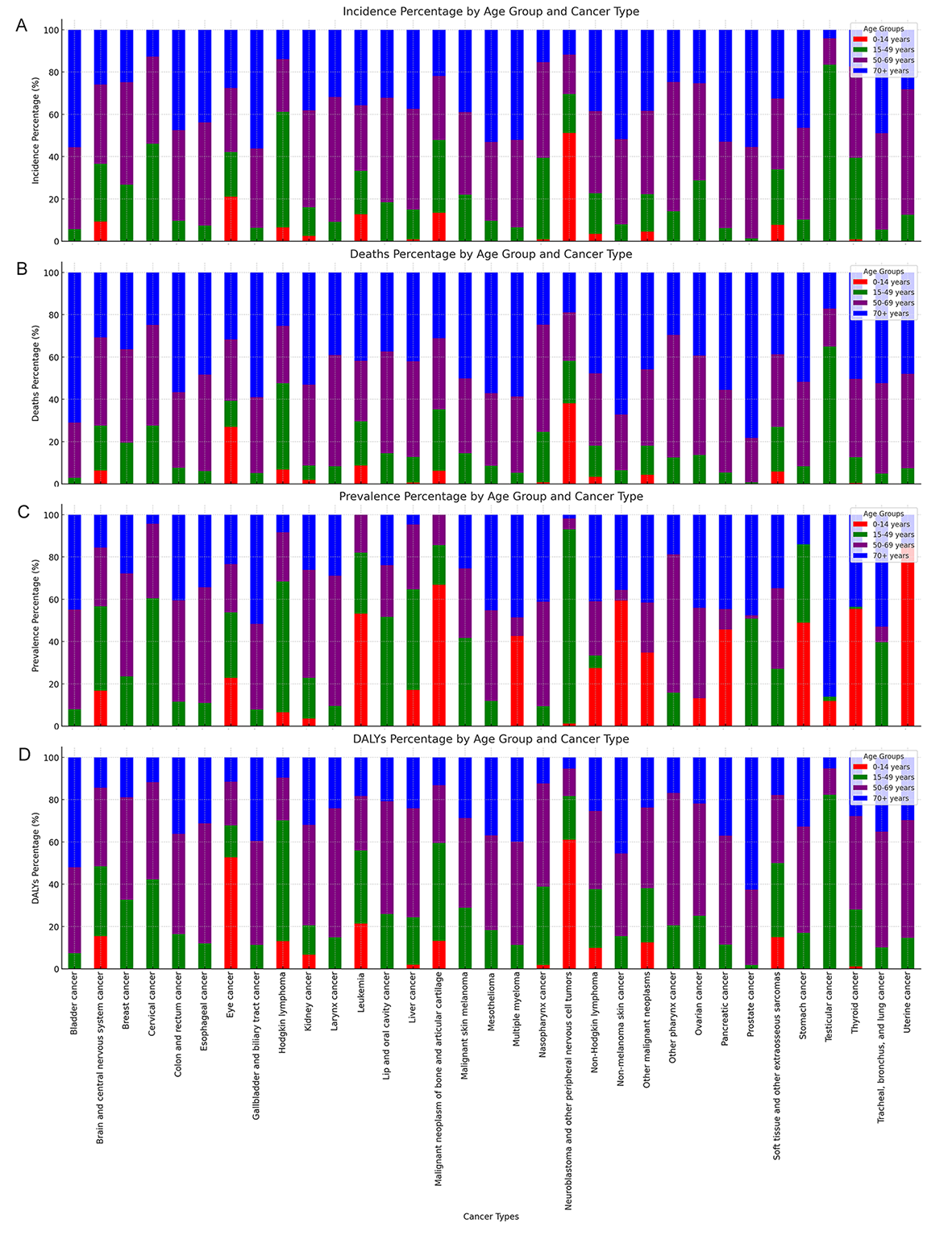


**Figure S3** Age-specific global contributions of 34 cancer types to total cancer incidence (A), mortality (B), prevalence (C), and disability-adjusted life years (DALYs) (D) in 2021.

**Table S3** Global incidence and deaths in 2021 for total cancers and 34 cancer types by age group

| **Cancer type** | **Deaths number** | | | | | | | | | | | | **Incidence number** | | | | | | | | | | | |
| --- | --- | --- | --- | --- | --- | --- | --- | --- | --- | --- | --- | --- | --- | --- | --- | --- | --- | --- | --- | --- | --- | --- | --- | --- |
|  | **0-14 years** | | | **15-49 years** | | | **50-69 years** | | | **70+ years** | | | **0-14 years** | | | **15-49 years** | | | **50-69 years** | | | **70+ years** | | |
|  | **Value** | **Upper** | **Lower** | **Value** | **Upper** | **Lower** | **Value** | **Upper** | **Lower** | **Value** | **Upper** | **Lower** | **Val** | **Upper** | **Lower** | **Val** | **Upper** | **Lower** | **Value** | **Upper** | **Lower** | **Value** | **Upper** | **Lower** |
| **Total cancer** | 83017.0 | 96869.3 | 69002.5 | 989646.0 | 1050744.6 | 927780.1 | 3904735.1 | 4201882.1 | 3644745.1 | 4855836.3 | 5207218.4 | 4339882.7 | 185528.1 | 216207.8 | 154645.8 | 3155265.2 | 3340461.5 | 2982068.6 | 10120326.1 | 10741401.0 | 9518336.1 | 10105125.9 | 10654323.6 | 9249089.3 |
| **Total cancer without NMSC** | 83017.0 | 96869.3 | 69002.5 | 985987.2 | 1046799.6 | 924234.5 | 3889717.0 | 4185616.5 | 3631067.2 | 4817600.0 | 5167416.9 | 4305738.7 | 185528.1 | 216207.8 | 154645.8 | 2647456.8 | 2807099.2 | 2501825.2 | 7576226.4 | 8080025.4 | 7132036.7 | 6820187.9 | 7290282.5 | 6125592.3 |
| **Bladder cancer** | 0.0 | 0.0 | 0.0 | 6327.8 | 6995.7 | 5732.8 | 57952.0 | 64027.6 | 53668.2 | 157608.5 | 171481.8 | 140182.0 | 0.0 | 0.0 | 0.0 | 31053.8 | 34322.0 | 28341.9 | 208853.9 | 226552.4 | 195053.9 | 300402.1 | 325843.4 | 269944.2 |
| **Brain and central nervous system cancer** | 16355.9 | 20197.0 | 13021.4 | 54848.6 | 64937.2 | 46387.7 | 107672.8 | 125934.2 | 93491.8 | 79749.6 | 88497.9 | 68592.5 | 33091.1 | 41046.8 | 27051.9 | 97455.2 | 113385.6 | 83231.1 | 134494.7 | 156582.5 | 117145.9 | 92441.3 | 102632.6 | 79569.1 |
| **Breast cancer** | 0.0 | 0.0 | 0.0 | 131024.0 | 140903.0 | 121841.4 | 297805.5 | 318945.7 | 277608.3 | 245369.9 | 266166.8 | 209950.0 | 0.0 | 0.0 | 0.0 | 567903.1 | 610272.3 | 530273.9 | 1025792.2 | 1101231.4 | 961613.5 | 527869.1 | 568777.6 | 451785.6 |
| **Cervical cancer** | 0.0 | 0.0 | 0.0 | 81639.9 | 90476.0 | 73783.0 | 141359.8 | 154507.2 | 129390.3 | 73667.6 | 80184.6 | 64384.6 | 0.0 | 0.0 | 0.0 | 307428.0 | 335692.3 | 280667.5 | 275134.3 | 300745.1 | 251861.4 | 84864.1 | 92662.0 | 73684.3 |
| **Colon and rectum cancer** | 0.0 | 0.0 | 0.0 | 79504.3 | 86539.5 | 72699.2 | 373101.3 | 403882.6 | 346700.4 | 591466.6 | 637242.6 | 524949.4 | 0.0 | 0.0 | 0.0 | 211890.4 | 231271.9 | 193832.2 | 940521.2 | 1019500.9 | 868586.7 | 1041731.7 | 1122489.4 | 929110.6 |
| **Esophageal cancer** | 0.0 | 0.0 | 0.0 | 32921.6 | 36952.6 | 29481.1 | 245400.0 | 279746.7 | 215828.5 | 260280.3 | 290326.6 | 227597.0 | 0.0 | 0.0 | 0.0 | 42698.1 | 47972.1 | 38138.3 | 280772.2 | 319304.0 | 246788.7 | 253058.9 | 282618.7 | 221430.1 |
| **Eye cancer** | 2806.3 | 3792.0 | 1706.4 | 1293.3 | 1721.5 | 925.0 | 3018.0 | 3765.6 | 2327.8 | 3315.2 | 3815.7 | 2646.9 | 7116.8 | 9315.2 | 4516.3 | 7187.4 | 9781.4 | 5104.4 | 10279.2 | 12492.4 | 8063.1 | 9367.3 | 10834.7 | 7498.2 |
| **Gallbladder and biliary tract cancer** | 0.0 | 0.0 | 0.0 | 8777.9 | 10235.1 | 6942.2 | 61673.2 | 71632.7 | 50378.4 | 101510.0 | 114027.6 | 84025.7 | 0.0 | 0.0 | 0.0 | 13611.9 | 15790.8 | 10670.0 | 81370.5 | 94432.7 | 66756.9 | 121786.0 | 136392.9 | 101987.3 |
| **Hodgkin lymphoma** | 1918.7 | 2587.7 | 1051.8 | 11492.9 | 15309.2 | 8145.0 | 7618.5 | 9703.5 | 5877.7 | 7149.8 | 8484.4 | 5778.1 | 4276.4 | 5567.5 | 2622.8 | 35594.7 | 42838.0 | 28925.5 | 16241.1 | 19129.7 | 13713.9 | 9069.8 | 10304.8 | 7607.0 |
| **Kidney cancer** | 3062.7 | 3898.0 | 2252.2 | 10977.6 | 11701.9 | 10264.4 | 61659.3 | 64762.8 | 58766.0 | 85495.0 | 90613.3 | 76676.8 | 9576.0 | 11648.7 | 7560.8 | 52631.4 | 55816.7 | 49672.9 | 177536.7 | 185927.6 | 169618.8 | 148084.6 | 156522.7 | 133539.2 |
| **Larynx cancer** | 0.0 | 0.0 | 0.0 | 9775.1 | 10868.7 | 8897.9 | 61589.6 | 66376.6 | 57369.0 | 45886.9 | 49266.5 | 42245.2 | 0.0 | 0.0 | 0.0 | 18427.5 | 20207.6 | 16895.6 | 118550.8 | 127297.2 | 110939.0 | 63904.7 | 68786.5 | 58639.3 |
| **Leukemia** | 27861.2 | 33393.5 | 20781.3 | 66686.1 | 74795.2 | 54524.9 | 91842.8 | 102171.2 | 78194.9 | 133893.5 | 145289.5 | 116714.6 | 58784.9 | 73139.0 | 43200.2 | 94727.6 | 105834.7 | 77223.9 | 142716.7 | 157698.4 | 123445.8 | 165193.6 | 177876.9 | 145497.2 |
| **Lip and oral cavity cancer** | 0.0 | 0.0 | 0.0 | 30270.0 | 33416.2 | 26153.5 | 100004.7 | 107421.5 | 91682.5 | 78104.7 | 83709.5 | 70723.6 | 0.0 | 0.0 | 0.0 | 77650.6 | 84643.3 | 68364.5 | 207944.8 | 221834.3 | 193646.8 | 135981.8 | 145777.3 | 122744.2 |
| **Liver cancer** | 2787.8 | 3461.3 | 2257.7 | 58825.1 | 68517.5 | 51340.4 | 218728.0 | 246991.2 | 197376.4 | 203534.2 | 222110.4 | 180704.2 | 4369.7 | 5380.0 | 3557.0 | 74947.9 | 87629.6 | 65248.4 | 251778.9 | 283481.0 | 227002.2 | 198106.0 | 216289.2 | 175788.7 |
| **Malignant neoplasm of bone and articular cartilage** | 4064.1 | 4871.6 | 3418.9 | 19262.0 | 22423.1 | 16198.9 | 22195.2 | 25539.3 | 17155.9 | 20593.0 | 23722.2 | 16346.1 | 12294.5 | 14565.8 | 10174.6 | 31390.7 | 35620.1 | 26019.5 | 27726.6 | 32283.9 | 21305.8 | 19963.3 | 23024.7 | 15691.3 |
| **Malignant skin melanoma** | 0.0 | 0.0 | 0.0 | 8928.1 | 10217.7 | 7241.9 | 21742.2 | 23590.1 | 19107.6 | 30879.5 | 33406.0 | 27275.4 | 0.0 | 0.0 | 0.0 | 66623.7 | 70407.8 | 60299.9 | 118330.2 | 123356.1 | 110818.6 | 118150.7 | 126379.5 | 104299.3 |
| **Mesothelioma** | 0.0 | 0.0 | 0.0 | 2533.3 | 2809.0 | 2264.5 | 10151.2 | 10938.7 | 9410.4 | 16934.4 | 18046.3 | 15273.9 | 0.0 | 0.0 | 0.0 | 3082.1 | 3400.5 | 2765.1 | 11870.0 | 12727.5 | 11042.6 | 16955.8 | 18050.7 | 15268.7 |
| **Multiple myeloma** | 0.0 | 0.0 | 0.0 | 6030.2 | 7141.1 | 4888.9 | 41991.5 | 48060.5 | 36617.4 | 68337.9 | 74602.2 | 59789.0 | 0.0 | 0.0 | 0.0 | 9766.9 | 11255.5 | 7977.6 | 61403.0 | 68828.5 | 53900.8 | 77584.7 | 84448.9 | 68296.0 |
| **Nasopharynx cancer** | 547.6 | 668.8 | 441.9 | 17967.3 | 20063.9 | 15840.4 | 38188.6 | 43019.0 | 33992.4 | 18655.1 | 20699.9 | 16581.7 | 966.1 | 1151.7 | 795.9 | 45877.6 | 52767.8 | 39882.6 | 53795.8 | 61829.8 | 46831.8 | 18238.5 | 20411.5 | 16061.3 |
| **Neuroblastoma and other peripheral nervous cell tumors** | 1976.9 | 2528.5 | 1445.0 | 1046.3 | 1149.2 | 922.7 | 1187.5 | 1310.4 | 1016.6 | 983.1 | 1088.9 | 824.1 | 5560.4 | 7560.0 | 3734.2 | 2000.8 | 2384.4 | 1661.0 | 2029.8 | 2456.8 | 1627.9 | 1276.5 | 1527.0 | 998.8 |
| **Non-Hodgkin lymphoma** | 9036.5 | 11252.0 | 7197.5 | 39135.0 | 43704.8 | 35266.1 | 91324.2 | 100819.4 | 84130.3 | 127565.6 | 136498.4 | 113101.6 | 20788.8 | 25305.8 | 17199.5 | 116613.1 | 126119.0 | 107011.3 | 234322.3 | 252586.2 | 215450.7 | 232829.9 | 251781.7 | 206058.1 |
| **Non-melanoma skin cancer** | 0.0 | 0.0 | 0.0 | 3658.8 | 4100.6 | 3053.3 | 15018.1 | 16865.7 | 12964.8 | 38236.3 | 42113.3 | 32406.6 | 0.0 | 0.0 | 0.0 | 507808.4 | 601464.1 | 419535.4 | 2544099.6 | 2905232.6 | 2196741.2 | 3284938.1 | 3657140.1 | 2933618.8 |
| **Other malignant neoplasms** | 9507.5 | 11644.2 | 7221.9 | 30699.8 | 33736.3 | 26948.6 | 80032.0 | 87425.1 | 72027.7 | 101969.1 | 110818.5 | 90855.1 | 19177.2 | 23703.0 | 14909.3 | 74619.8 | 81336.4 | 65898.2 | 166398.4 | 182170.9 | 150934.8 | 162099.7 | 177722.5 | 144381.5 |
| **Other neoplasms** | 322.8 | 486.9 | 233.6 | 1933.2 | 2840.9 | 1514.7 | 9499.7 | 12554.9 | 8069.2 | 43423.5 | 48861.4 | 36311.3 | 3179977.3 | 4341646.6 | 2243371.1 | 20455405.4 | 26113404.9 | 15506514.5 | 14223240.0 | 19241891.5 | 10245051.6 | 5054739.2 | 6929863.5 | 3546182.8 |
| **Other pharynx cancer** | 0.0 | 0.0 | 0.0 | 12267.3 | 13580.2 | 10932.6 | 57096.1 | 61406.4 | 52953.9 | 29071.7 | 31205.7 | 26832.8 | 0.0 | 0.0 | 0.0 | 23931.1 | 25969.1 | 21929.1 | 103927.4 | 110155.3 | 97754.7 | 41961.1 | 44508.7 | 38982.7 |
| **Ovarian cancer** | 0.0 | 0.0 | 0.0 | 25257.8 | 27861.2 | 22277.5 | 87360.3 | 95316.6 | 80072.0 | 72990.6 | 79705.6 | 63113.8 | 0.0 | 0.0 | 0.0 | 85748.8 | 95089.8 | 75168.9 | 137310.0 | 149686.1 | 125575.5 | 75817.1 | 82584.5 | 65626.3 |
| **Pancreatic cancer** | 0.0 | 0.0 | 0.0 | 26995.6 | 29597.7 | 24492.8 | 197411.9 | 213739.7 | 182275.9 | 281344.7 | 303317.5 | 247576.8 | 0.0 | 0.0 | 0.0 | 31530.7 | 34517.4 | 28670.6 | 207675.4 | 224653.1 | 191775.2 | 269326.6 | 291006.9 | 235599.9 |
| **Prostate cancer** | 0.0 | 0.0 | 0.0 | 2860.9 | 3241.7 | 2262.8 | 90962.7 | 99553.9 | 77030.1 | 338639.7 | 362940.9 | 298294.3 | 0.0 | 0.0 | 0.0 | 17865.1 | 19483.1 | 15617.1 | 571845.3 | 603899.8 | 527379.4 | 734672.6 | 779147.8 | 669125.6 |
| **Soft tissue and other extraosseous sarcomas** | 2940.7 | 4301.2 | 2211.6 | 10580.4 | 13497.3 | 9014.7 | 17242.1 | 20876.7 | 14747.9 | 19440.0 | 22873.5 | 17014.1 | 7468.1 | 10675.6 | 5679.2 | 25158.1 | 31356.4 | 21503.0 | 32199.4 | 38008.0 | 27764.2 | 31375.4 | 35861.7 | 27233.0 |
| **Stomach cancer** | 0.0 | 0.0 | 0.0 | 78870.9 | 90835.6 | 68703.7 | 380833.5 | 441894.1 | 328380.1 | 494669.2 | 556323.2 | 423912.3 | 0.0 | 0.0 | 0.0 | 125120.5 | 144782.6 | 107273.8 | 533607.4 | 621822.4 | 457419.4 | 571504.7 | 645384.7 | 491012.1 |
| **Testicular cancer** | 0.0 | 0.0 | 0.0 | 7391.3 | 7843.5 | 6957.1 | 2051.3 | 2198.1 | 1922.5 | 1945.7 | 2106.4 | 1762.5 | 0.0 | 0.0 | 0.0 | 76363.7 | 79920.9 | 73293.7 | 11403.7 | 12108.8 | 10791.2 | 3740.0 | 4067.1 | 3401.5 |
| **Thyroid cancer** | 151.3 | 180.1 | 117.7 | 5499.4 | 6408.2 | 4692.5 | 16573.4 | 17895.0 | 14493.0 | 22574.5 | 24449.2 | 19674.6 | 2058.2 | 2448.9 | 1670.2 | 96287.8 | 110261.1 | 83996.6 | 107985.6 | 117295.3 | 97138.6 | 43206.4 | 46522.0 | 38476.2 |
| **Tracheal, bronchus, and lung cancer** | 0.0 | 0.0 | 0.0 | 99134.1 | 109945.7 | 88234.6 | 860321.8 | 948270.9 | 775590.5 | 1057091.5 | 1156023.0 | 941474.2 | 0.0 | 0.0 | 0.0 | 123408.6 | 137016.8 | 109786.3 | 1041008.6 | 1149875.3 | 938151.8 | 1116271.1 | 1221802.6 | 992421.5 |
| **Uterine cancer** | 0.0 | 0.0 | 0.0 | 7163.4 | 8042.2 | 5983.6 | 43625.9 | 48710.5 | 39209.4 | 46882.8 | 51531.6 | 40691.8 | 0.0 | 0.0 | 0.0 | 58860.1 | 65452.4 | 50765.4 | 281400.6 | 305176.6 | 259327.7 | 133353.1 | 144317.2 | 115899.9 |

**Table S4** Global incidence, prevalence, deaths, and DALYs in 2021 for 34 cancer types by age group

| **Age** | **Cause** | **Deaths** | **DALYs** | **Incidence** | **Prevalence** |
| --- | --- | --- | --- | --- | --- |
| 70+ years | Bladder cancer | 157608.5 | 2286683.8 | 300402.1 | 1360407.1 |
| 50-69 years | Bladder cancer | 57952.0 | 1786345.2 | 208853.9 | 1425962.7 |
| 15-49 years | Bladder cancer | 6327.8 | 324037.7 | 31053.8 | 239183.7 |
| 0-14 years | Bladder cancer | 0.0 | 0.0 | 0.0 | 0.0 |
| 70+ years | Brain and central nervous system cancer | 79749.6 | 1279809.9 | 92441.3 | 152487.4 |
| 50-69 years | Brain and central nervous system cancer | 107672.8 | 3318300.3 | 134494.7 | 270864.0 |
| 15-49 years | Brain and central nervous system cancer | 54848.6 | 2943175.8 | 97455.2 | 389048.7 |
| 0-14 years | Brain and central nervous system cancer | 16355.9 | 1371309.3 | 33091.1 | 162879.2 |
| 70+ years | Breast cancer | 245369.9 | 3901538.5 | 527869.1 | 5733999.4 |
| 50-69 years | Breast cancer | 297805.5 | 9991109.4 | 1025792.2 | 10051241.2 |
| 15-49 years | Breast cancer | 131024.0 | 6743070.3 | 567903.1 | 4858397.3 |
| 0-14 years | Breast cancer | 0.0 | 0.0 | 0.0 | 0.0 |
| 70+ years | Cervical cancer | 73667.6 | 1169093.1 | 84864.1 | 145721.9 |
| 50-69 years | Cervical cancer | 141359.8 | 4558245.8 | 275134.3 | 1192049.0 |
| 15-49 years | Cervical cancer | 81639.9 | 4184314.2 | 307428.0 | 2046773.0 |
| 0-14 years | Cervical cancer | 0.0 | 0.0 | 0.0 | 0.0 |
| 70+ years | Colon and rectum cancer | 591466.6 | 8849861.7 | 1041731.7 | 4736961.9 |
| 50-69 years | Colon and rectum cancer | 373101.3 | 11548482.7 | 940521.2 | 5599689.8 |
| 15-49 years | Colon and rectum cancer | 79504.3 | 4002755.8 | 211890.4 | 1342467.8 |
| 0-14 years | Colon and rectum cancer | 0.0 | 0.0 | 0.0 | 0.0 |
| 70+ years | Esophageal cancer | 260280.3 | 4057305.0 | 253058.9 | 344328.9 |
| 50-69 years | Esophageal cancer | 245400.0 | 7390044.7 | 280772.2 | 550544.4 |
| 15-49 years | Esophageal cancer | 32921.6 | 1551915.1 | 42698.1 | 109328.7 |
| 0-14 years | Esophageal cancer | 0.0 | 0.0 | 0.0 | 0.0 |
| 70+ years | Eye cancer | 3315.2 | 54309.0 | 9367.3 | 63371.3 |
| 50-69 years | Eye cancer | 3018.0 | 96610.1 | 10279.2 | 85999.7 |
| 15-49 years | Eye cancer | 1293.3 | 70756.7 | 7187.4 | 63160.0 |
| 0-14 years | Eye cancer | 2806.3 | 247072.7 | 7116.8 | 64988.8 |
| 70+ years | Gallbladder and biliary tract cancer | 101510.0 | 1478247.4 | 121786.0 | 162452.5 |
| 50-69 years | Gallbladder and biliary tract cancer | 61673.2 | 1833347.3 | 81370.5 | 127457.1 |
| 15-49 years | Gallbladder and biliary tract cancer | 8777.9 | 420526.6 | 13611.9 | 24555.6 |
| 0-14 years | Gallbladder and biliary tract cancer | 0.0 | 0.0 | 0.0 | 0.0 |
| 70+ years | Hodgkin lymphoma | 7149.8 | 114669.3 | 9069.8 | 30346.1 |
| 50-69 years | Hodgkin lymphoma | 7618.5 | 241880.2 | 16241.1 | 83784.9 |
| 15-49 years | Hodgkin lymphoma | 11492.9 | 683027.6 | 35594.7 | 224024.3 |
| 0-14 years | Hodgkin lymphoma | 1918.7 | 156611.1 | 4276.4 | 23423.6 |
| 70+ years | Kidney cancer | 85495.0 | 1283283.7 | 148084.6 | 513188.2 |
| 50-69 years | Kidney cancer | 61659.3 | 1905983.7 | 177536.7 | 1000684.9 |
| 15-49 years | Kidney cancer | 10977.6 | 559045.8 | 52631.4 | 377955.1 |
| 0-14 years | Kidney cancer | 3062.7 | 268048.9 | 9576.0 | 69382.7 |
| 70+ years | Larynx cancer | 45886.9 | 757942.2 | 63904.7 | 318415.2 |
| 50-69 years | Larynx cancer | 61589.6 | 1917586.1 | 118550.8 | 680053.4 |
| 15-49 years | Larynx cancer | 9775.1 | 467780.4 | 18427.5 | 105215.0 |
| 0-14 years | Larynx cancer | 0.0 | 0.0 | 0.0 | 0.0 |
| 70+ years | Leukemia | 133893.5 | 2013619.7 | 165193.6 | 495677.1 |
| 50-69 years | Leukemia | 91842.8 | 2823298.5 | 142716.7 | 570497.5 |
| 15-49 years | Leukemia | 66686.1 | 3804308.0 | 94727.6 | 341406.1 |
| 0-14 years | Leukemia | 27861.2 | 2341610.0 | 58784.9 | 308329.8 |
| 70+ years | Lip and oral cavity cancer | 78104.7 | 1222870.7 | 135981.8 | 377130.7 |
| 50-69 years | Lip and oral cavity cancer | 100004.7 | 3131217.6 | 207944.8 | 793761.1 |
| 15-49 years | Lip and oral cavity cancer | 30270.0 | 1519981.4 | 77650.6 | 367115.9 |
| 0-14 years | Lip and oral cavity cancer | 0.0 | 0.0 | 0.0 | 0.0 |
| 70+ years | Liver cancer | 203534.2 | 3105783.7 | 198106.0 | 226295.5 |
| 50-69 years | Liver cancer | 218728.0 | 6650019.7 | 251778.9 | 351826.4 |
| 15-49 years | Liver cancer | 58825.1 | 2889491.6 | 74947.9 | 126714.5 |
| 0-14 years | Liver cancer | 2787.8 | 242357.4 | 4369.7 | 34463.1 |
| 70+ years | Malignant neoplasm of bone and articular cartilage | 20593.0 | 332532.8 | 19963.3 | 112090.6 |
| 50-69 years | Malignant neoplasm of bone and articular cartilage | 22195.2 | 690460.3 | 27726.6 | 181238.6 |
| 15-49 years | Malignant neoplasm of bone and articular cartilage | 19262.0 | 1170301.2 | 31390.7 | 219149.0 |
| 0-14 years | Malignant neoplasm of bone and articular cartilage | 4064.1 | 332533.5 | 12294.5 | 86159.5 |
| 70+ years | Malignant skin melanoma | 30879.5 | 483532.1 | 118150.7 | 715760.8 |
| 50-69 years | Malignant skin melanoma | 21742.2 | 710617.0 | 118330.2 | 908300.6 |
| 15-49 years | Malignant skin melanoma | 8928.1 | 484687.2 | 66623.7 | 553504.8 |
| 0-14 years | Malignant skin melanoma | 0.0 | 0.0 | 0.0 | 0.0 |
| 70+ years | Mesothelioma | 16934.4 | 255030.8 | 16955.8 | 23372.8 |
| 50-69 years | Mesothelioma | 10151.2 | 308167.5 | 11870.0 | 22122.6 |
| 15-49 years | Mesothelioma | 2533.3 | 126160.6 | 3082.1 | 6101.2 |
| 0-14 years | Mesothelioma | 0.0 | 0.0 | 0.0 | 0.0 |
| 70+ years | Multiple myeloma | 68337.9 | 1037150.9 | 77584.7 | 168342.6 |
| 50-69 years | Multiple myeloma | 41991.5 | 1264419.1 | 61403.0 | 191612.3 |
| 15-49 years | Multiple myeloma | 6030.2 | 294025.0 | 9766.9 | 34526.9 |
| 0-14 years | Multiple myeloma | 0.0 | 0.0 | 0.0 | 0.0 |
| 70+ years | Nasopharynx cancer | 18655.1 | 307236.7 | 18238.5 | 44564.4 |
| 50-69 years | Nasopharynx cancer | 38188.6 | 1217021.4 | 53795.8 | 216311.7 |
| 15-49 years | Nasopharynx cancer | 17967.3 | 922320.7 | 45877.6 | 259727.3 |
| 0-14 years | Nasopharynx cancer | 547.6 | 43612.7 | 966.1 | 4615.7 |
| 70+ years | Neuroblastoma and other peripheral nervous cell tumors | 983.1 | 15279.2 | 1276.5 | 638.2 |
| 50-69 years | Neuroblastoma and other peripheral nervous cell tumors | 1187.5 | 37002.8 | 2029.8 | 1014.9 |
| 15-49 years | Neuroblastoma and other peripheral nervous cell tumors | 1046.3 | 59010.4 | 2000.8 | 2910.3 |
| 0-14 years | Neuroblastoma and other peripheral nervous cell tumors | 1976.9 | 174186.3 | 5560.4 | 51762.4 |
| 70+ years | Non-Hodgkin lymphoma | 127565.6 | 1971280.5 | 232829.9 | 799432.9 |
| 50-69 years | Non-Hodgkin lymphoma | 91324.2 | 2870341.7 | 234322.3 | 1194792.3 |
| 15-49 years | Non-Hodgkin lymphoma | 39135.0 | 2163355.6 | 116613.1 | 751722.5 |
| 0-14 years | Non-Hodgkin lymphoma | 9036.5 | 761085.4 | 20788.8 | 173103.9 |
| 70+ years | Non-melanoma skin cancer | 38236.3 | 551936.4 | 3284938.1 | 1637887.5 |
| 50-69 years | Non-melanoma skin cancer | 15018.1 | 473600.3 | 2544099.6 | 983837.9 |
| 15-49 years | Non-melanoma skin cancer | 3658.8 | 187335.7 | 507808.4 | 139178.0 |
| 0-14 years | Non-melanoma skin cancer |  | 0.0 | 0.0 | 0.0 |
| 70+ years | Other malignant neoplasms | 101969.1 | 1534002.2 | 162099.7 | 519501.2 |
| 50-69 years | Other malignant neoplasms | 80032.0 | 2462991.3 | 166398.4 | 746645.6 |
| 15-49 years | Other malignant neoplasms | 30699.8 | 1649409.4 | 74619.8 | 424063.2 |
| 0-14 years | Other malignant neoplasms | 9507.5 | 808343.0 | 19177.2 | 103541.1 |
| 70+ years | Other neoplasms | 43423.5 | 654806.9 | 5054739.2 | 11425274.0 |
| 50-69 years | Other neoplasms | 9499.7 | 384317.0 | 14223240.0 | 23997727.4 |
| 15-49 years | Other neoplasms | 1933.2 | 142162.1 | 20455405.4 | 35054217.3 |
| 0-14 years | Other neoplasms | 322.8 | 34162.7 | 3179977.3 | 4575974.4 |
| 70+ years | Other pharynx cancer | 29071.7 | 481847.2 | 41961.1 | 61721.5 |
| 50-69 years | Other pharynx cancer | 57096.1 | 1778300.2 | 103927.4 | 214562.4 |
| 15-49 years | Other pharynx cancer | 12267.3 | 583633.4 | 23931.1 | 51964.9 |
| 0-14 years | Other pharynx cancer | 0.0 | 0.0 | 0.0 | 0.0 |
| 70+ years | Ovarian cancer | 72990.6 | 1131948.3 | 75817.1 | 161100.6 |
| 50-69 years | Ovarian cancer | 87360.3 | 2736312.4 | 137310.0 | 539006.0 |
| 15-49 years | Ovarian cancer | 25257.8 | 1294995.6 | 85748.8 | 522318.7 |
| 0-14 years | Ovarian cancer | 0.0 | 0.0 | 0.0 | 0.0 |
| 70+ years | Pancreatic cancer | 281344.7 | 4194809.8 | 269326.6 | 200719.8 |
| 50-69 years | Pancreatic cancer | 197411.9 | 5836979.6 | 207675.4 | 196026.6 |
| 15-49 years | Pancreatic cancer | 26995.6 | 1285173.9 | 31530.7 | 42254.2 |
| 0-14 years | Pancreatic cancer | 0.0 | 0.0 | 0.0 | 0.0 |
| 70+ years | Prostate cancer | 338639.7 | 5097742.7 | 734672.6 | 5275652.1 |
| 50-69 years | Prostate cancer | 90962.7 | 2898053.9 | 571845.3 | 4949052.7 |
| 15-49 years | Prostate cancer | 2860.9 | 145764.5 | 17865.1 | 162815.7 |
| 0-14 years | Prostate cancer | 0.0 | 0.0 | 0.0 | 0.0 |
| 70+ years | Soft tissue and other extraosseous sarcomas | 19440.0 | 300155.9 | 31375.4 | 130247.6 |
| 50-69 years | Soft tissue and other extraosseous sarcomas | 17242.1 | 538365.4 | 32199.4 | 166992.7 |
| 15-49 years | Soft tissue and other extraosseous sarcomas | 10580.4 | 588809.9 | 25158.1 | 139915.0 |
| 0-14 years | Soft tissue and other extraosseous sarcomas | 2940.7 | 250560.7 | 7468.1 | 43318.2 |
| 70+ years | Stomach cancer | 494669.2 | 7473462.1 | 571504.7 | 885537.2 |
| 50-69 years | Stomach cancer | 380833.5 | 11454134.9 | 533607.4 | 1171365.5 |
| 15-49 years | Stomach cancer | 78870.9 | 3859036.1 | 125120.5 | 336310.6 |
| 0-14 years | Stomach cancer | 0.0 | 0.0 | 0.0 | 0.0 |
| 70+ years | Testicular cancer | 1945.7 | 29676.6 | 3740.0 | 14013.3 |
| 50-69 years | Testicular cancer | 2051.3 | 69878.6 | 11403.7 | 80393.1 |
| 15-49 years | Testicular cancer | 7391.3 | 461366.3 | 76363.7 | 584696.5 |
| 0-14 years | Testicular cancer | 0.0 | 0.0 | 0.0 | 0.0 |
| 70+ years | Thyroid cancer | 22574.5 | 346615.9 | 43206.4 | 206460.9 |
| 50-69 years | Thyroid cancer | 16573.4 | 550929.2 | 107985.6 | 894584.4 |
| 15-49 years | Thyroid cancer | 5499.4 | 335885.1 | 96287.8 | 867504.0 |
| 0-14 years | Thyroid cancer | 151.3 | 13054.6 | 2058.2 | 18599.2 |
| 70+ years | Tracheal, bronchus, and lung cancer | 1057091.5 | 16376343.8 | 1116271.1 | 1288561.2 |
| 50-69 years | Tracheal, bronchus, and lung cancer | 860321.8 | 25438473.7 | 1041008.6 | 1723746.9 |
| 15-49 years | Tracheal, bronchus, and lung cancer | 99134.1 | 4721454.6 | 123408.6 | 241421.1 |
| 0-14 years | Tracheal, bronchus, and lung cancer | 0.0 | 0.0 | 0.0 | 0.0 |
| 70+ years | Uterine cancer | 46882.8 | 760285.6 | 133353.1 | 764456.1 |
| 50-69 years | Uterine cancer | 43625.9 | 1428975.7 | 281400.6 | 2203426.1 |
| 15-49 years | Uterine cancer | 7163.4 | 373681.7 | 58860.1 | 483867.7 |
| 0-14 years | Uterine cancer | 0.0 | 0.0 | 0.0 | 0.0 |


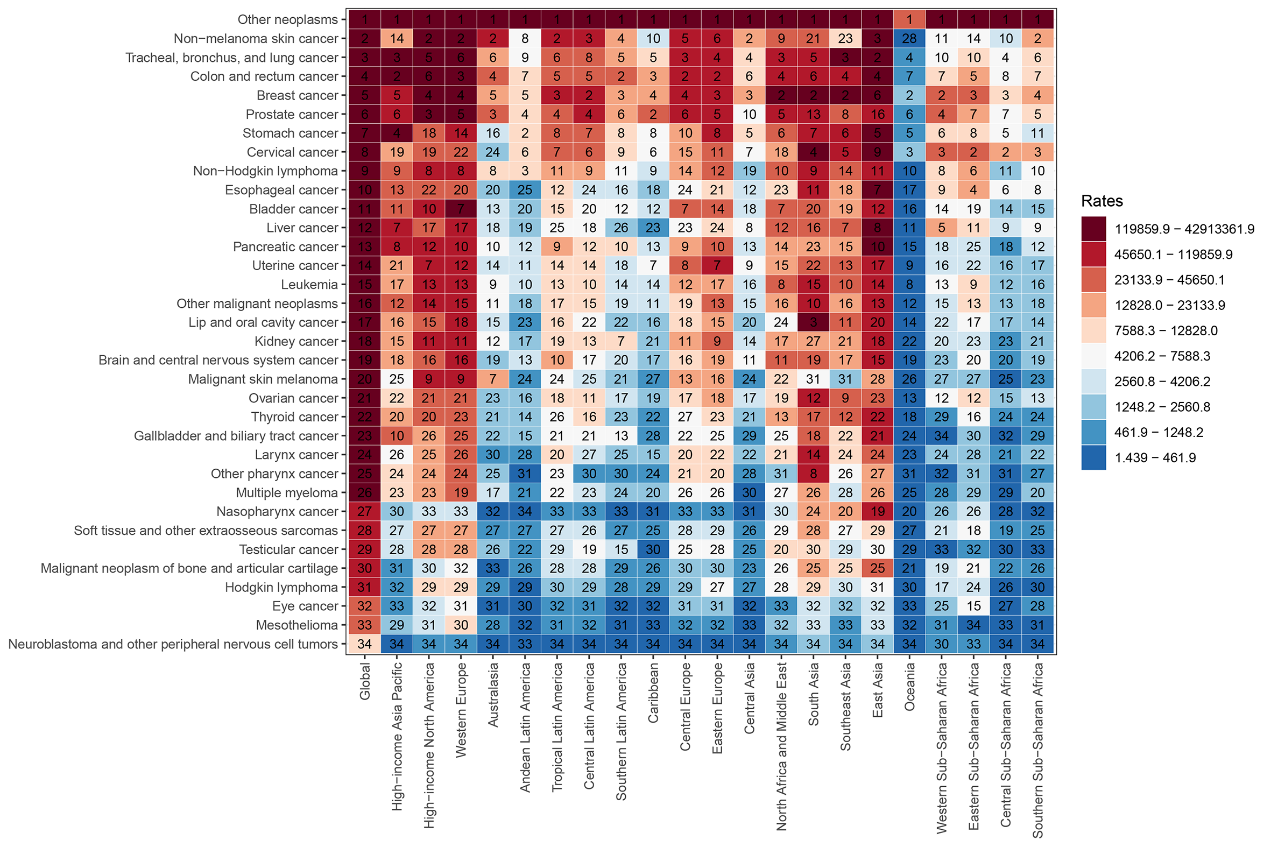


**Figure S4.** 34 specific cancer types ranked by the number of incident cases globally and across 21 global regions in 2021


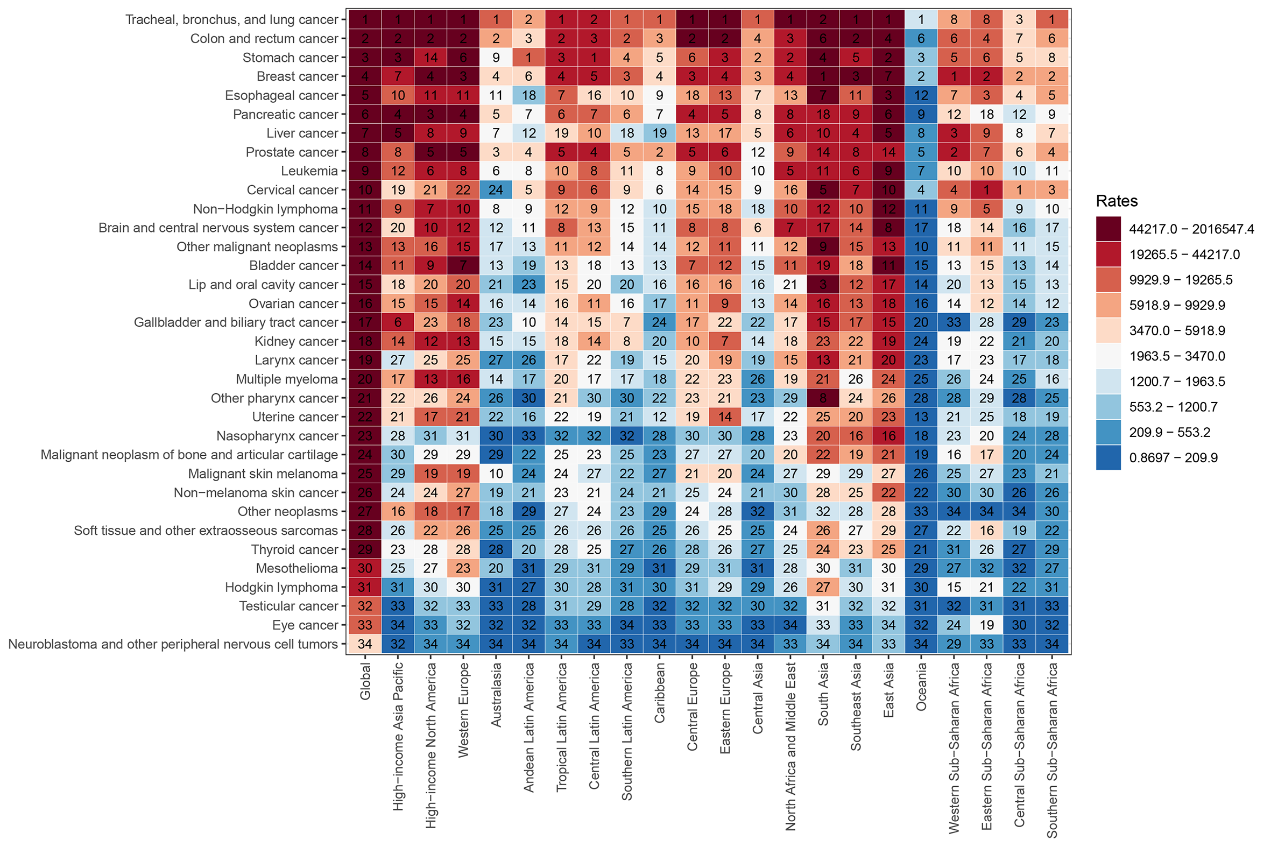


**Figure S5.** 34 specific cancer types ranked by the number of deaths globally and across 21 global regions in 2021


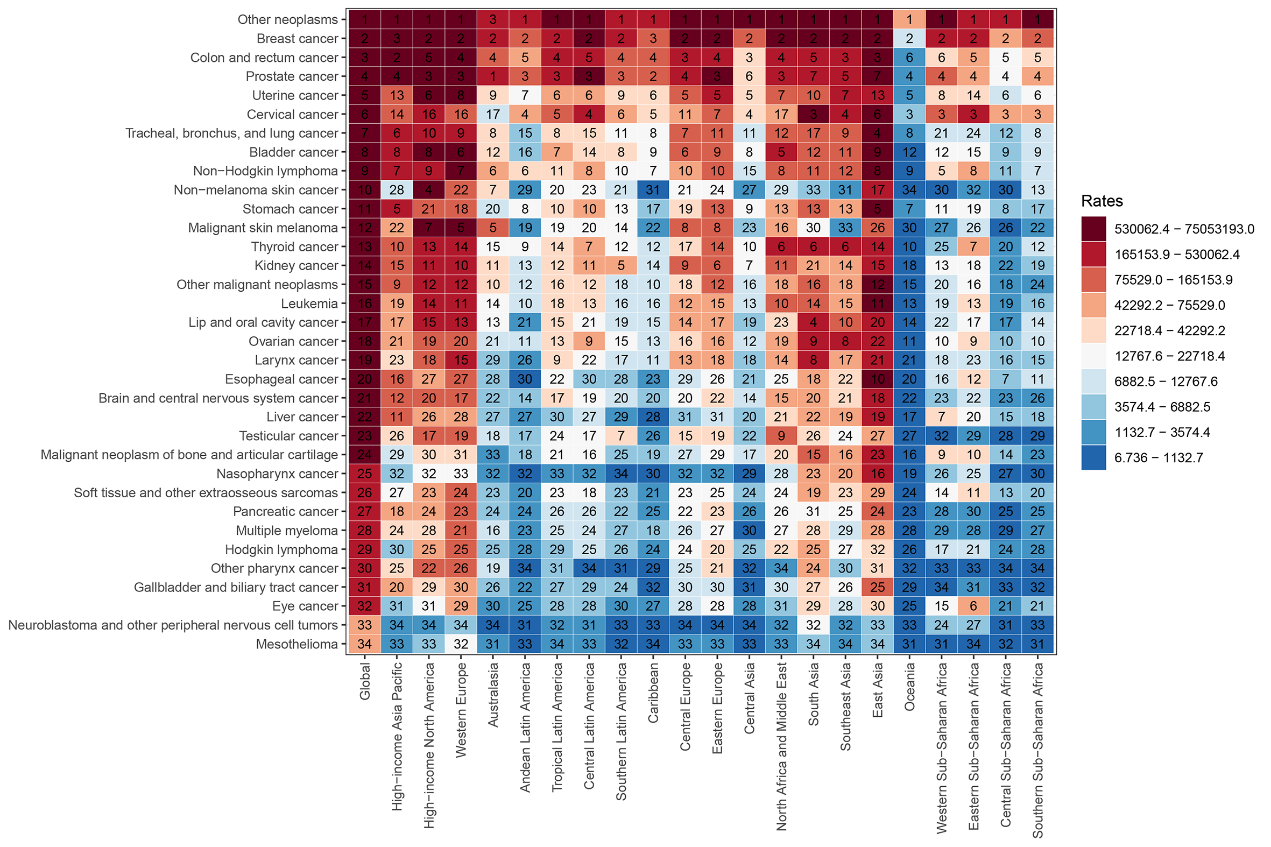


**Figure S6.** 34 specific cancer types ranked by the number of prevalent cases globally and across 21 regions in 2021


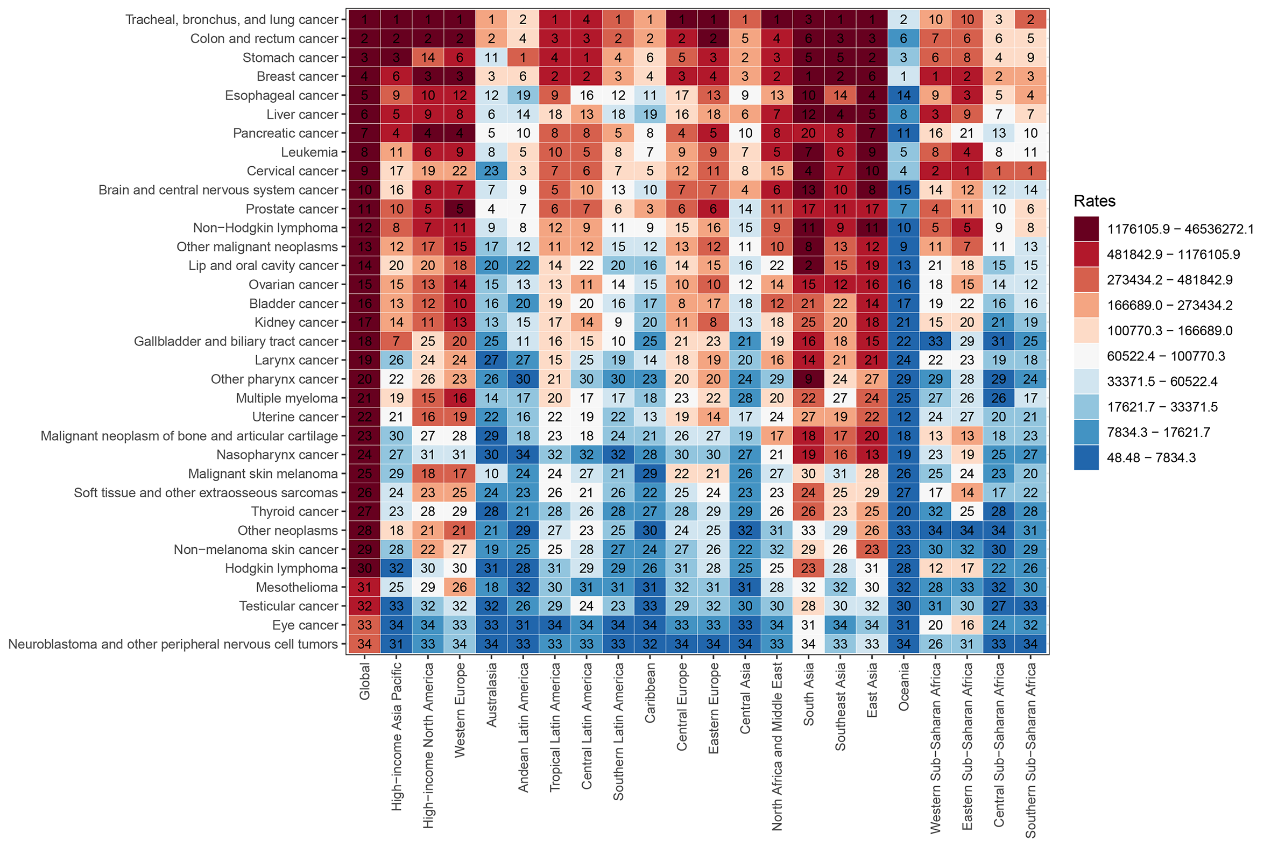


**Figure S7.** 34 specific cancer types ranked by DALYs losses globally and across 21 regions in 2021


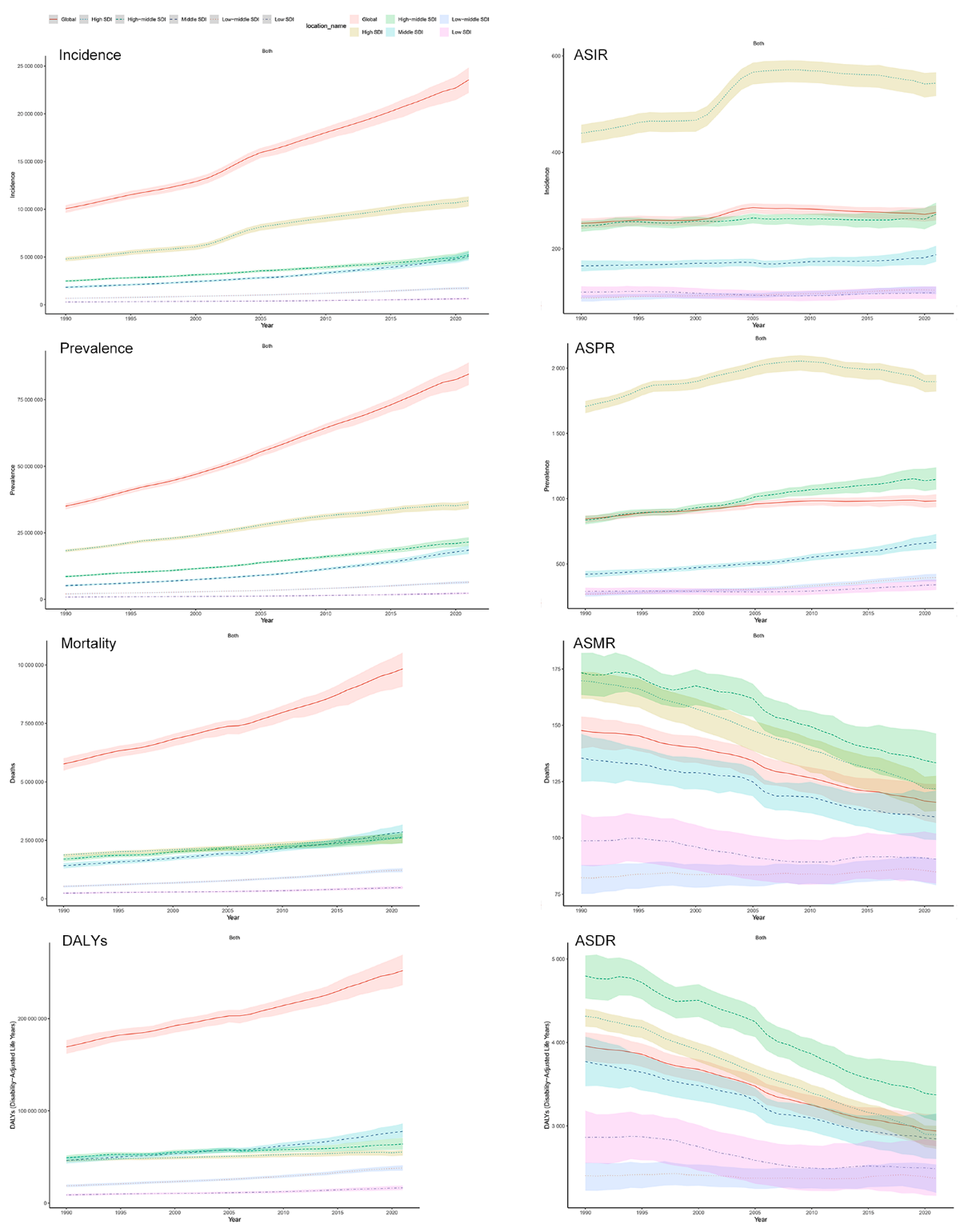


**Figure S8.** Trends in global cancer incidence, mortality, prevalence, and disability-adjusted life years (DALYs), along with age-standardized incidence, mortality, prevalence, and DALYs from 1990 to 2021, by sociodemographic index status


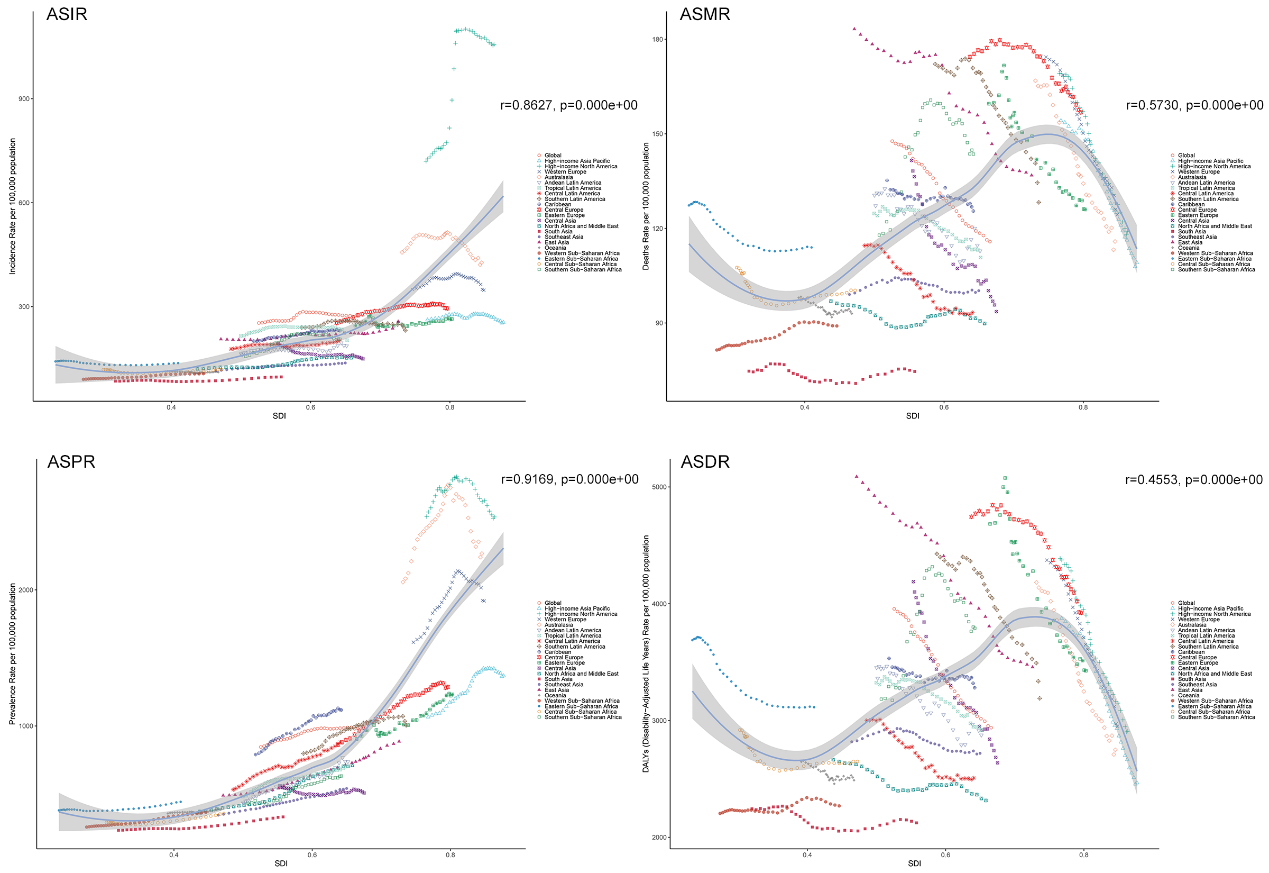


**Figure S9.** Correlations of the sociodemographic index with age-standardized global cancer incidence, mortality, prevalence, and DALYs across 21 global regions in 2021


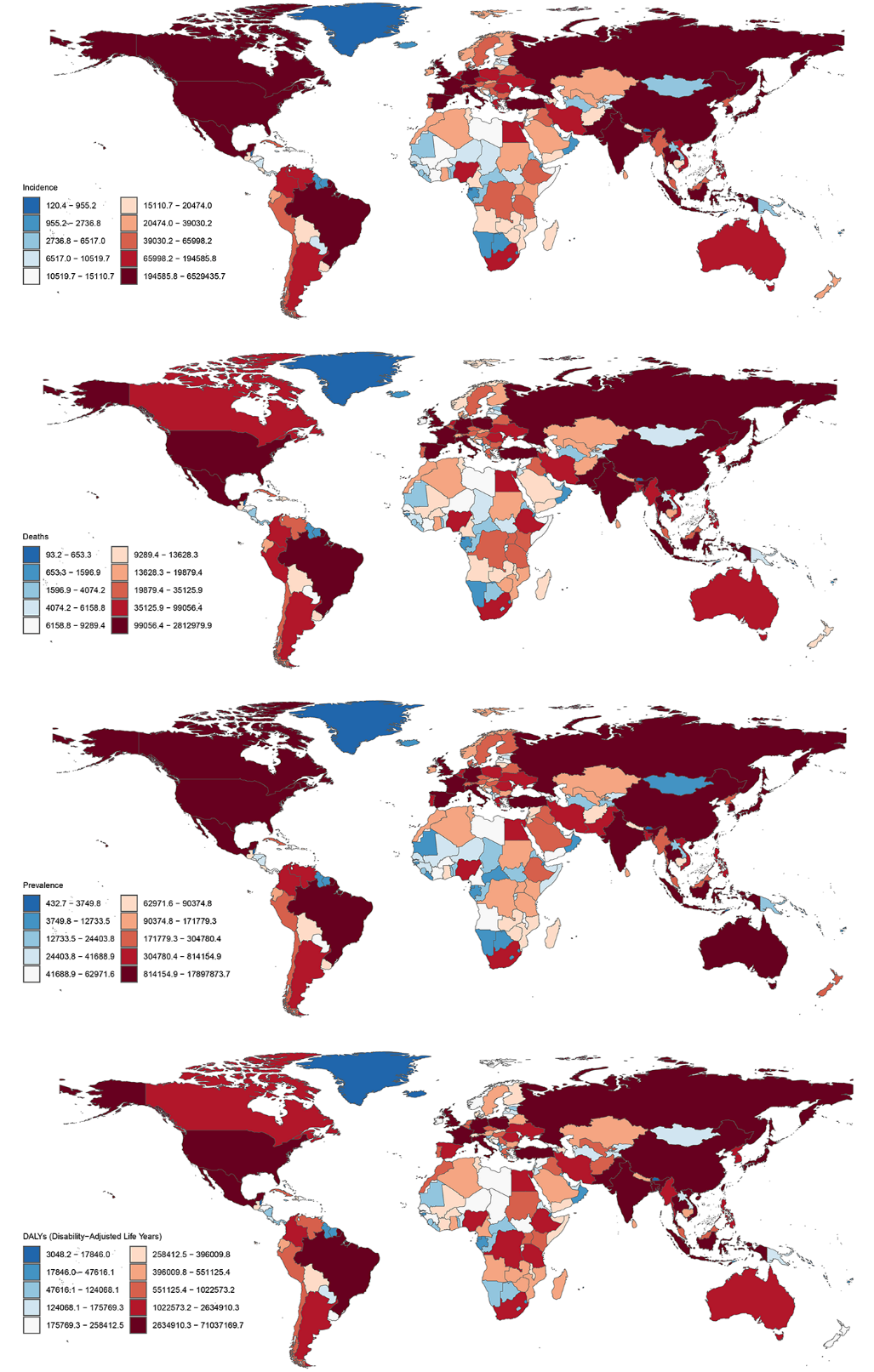


**Figure S10.** Global maps of cancer incidence, mortality, prevalence, and disability-adjusted life years (D) for total cancer, both sexes combined, in 2021


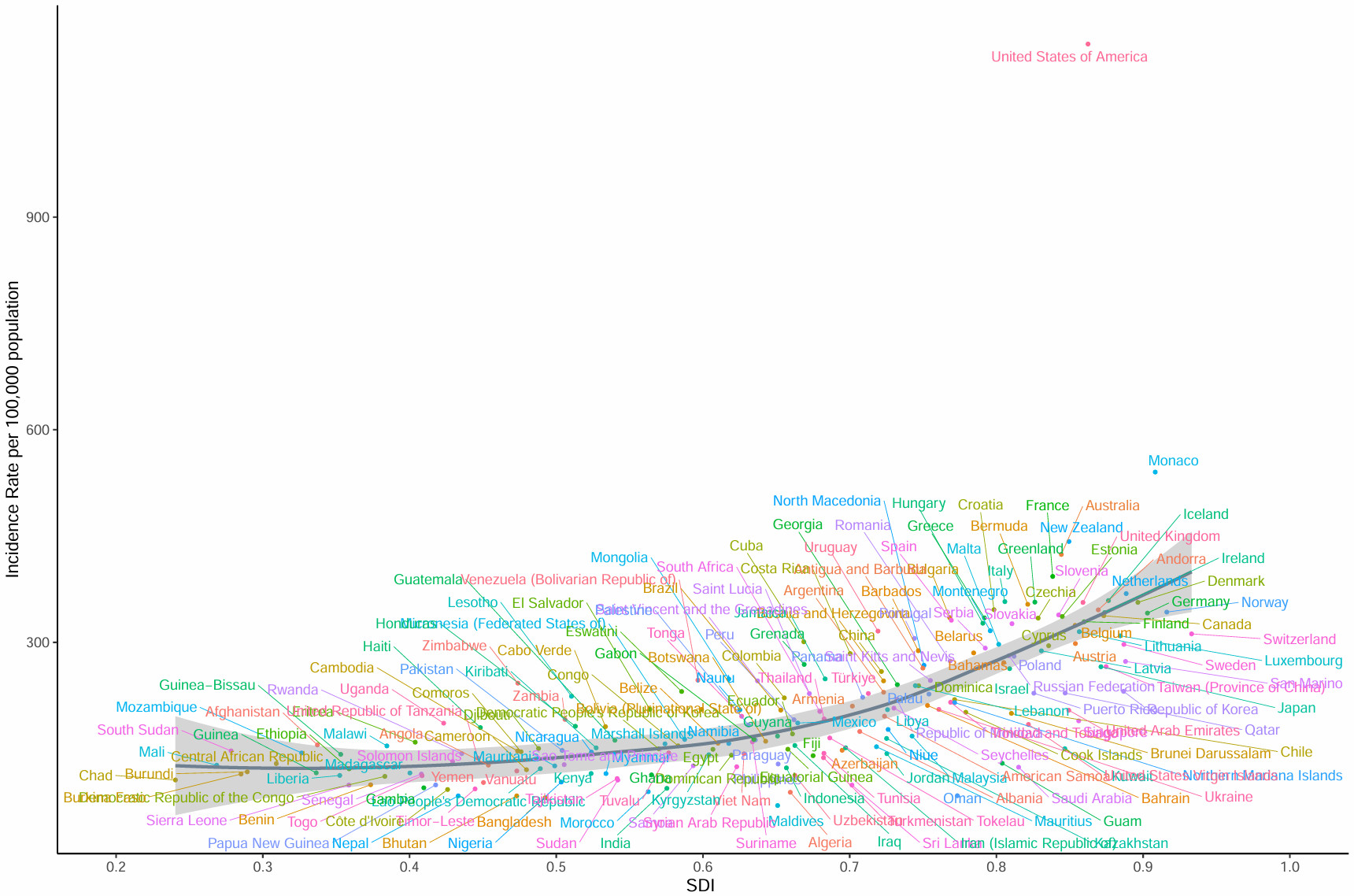


**Figure S11.** Correlations of the sociodemographic index with age-standardised global cancer incidence for total cancers in 204 countries and territories (r = 0.78, p < 0.001)


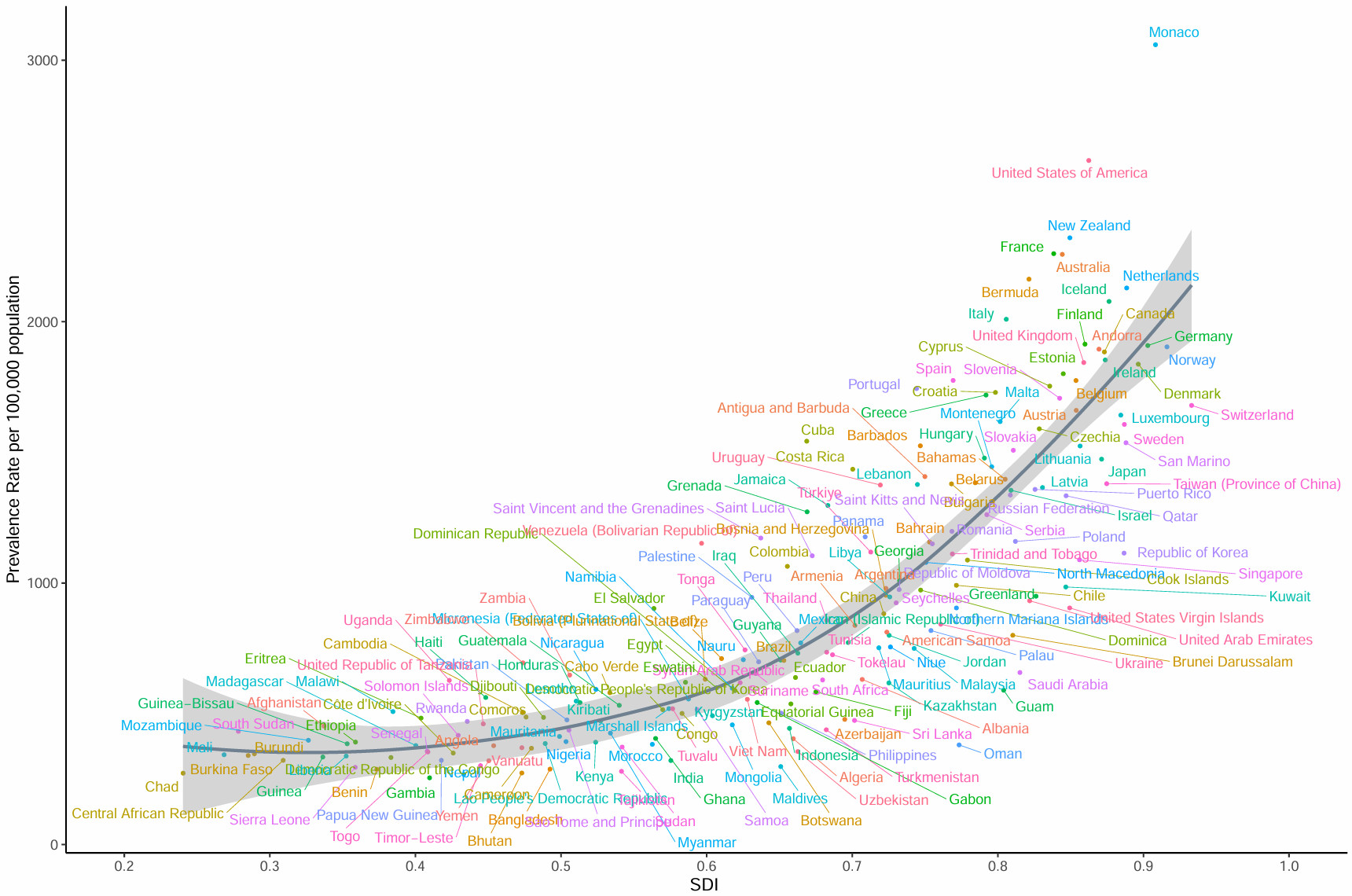


**Figure S12.** Correlations of the sociodemographic index with age-standardised global cancer prevalence for total cancers in 204 countries and territories (r = 0.88, p < 0.001)


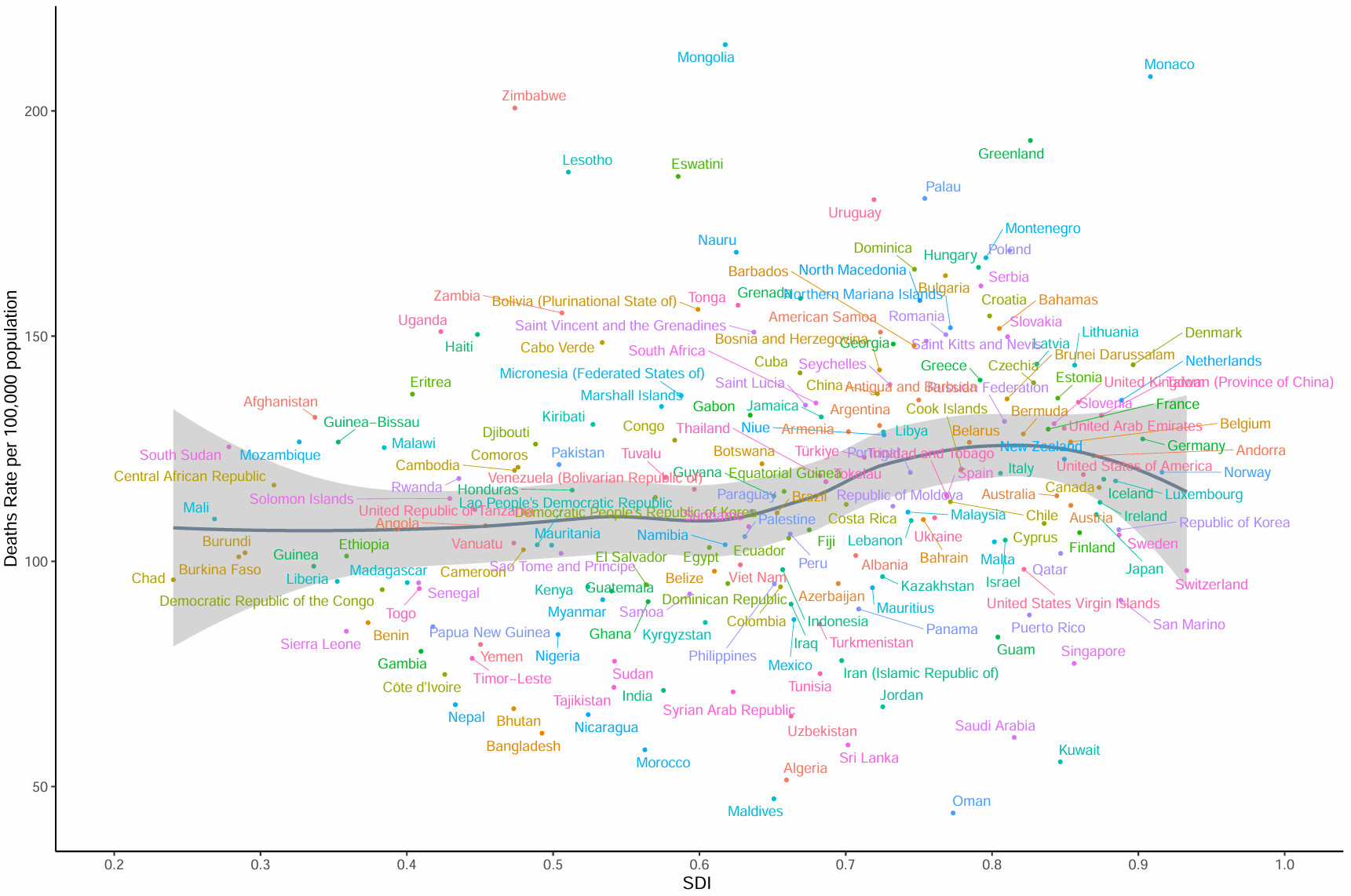


**Figure S13.** Correlations of the sociodemographic index with age-standardised global cancer mortality for total cancers in 204 countries and territories (r = 0.27, p < 0.001)


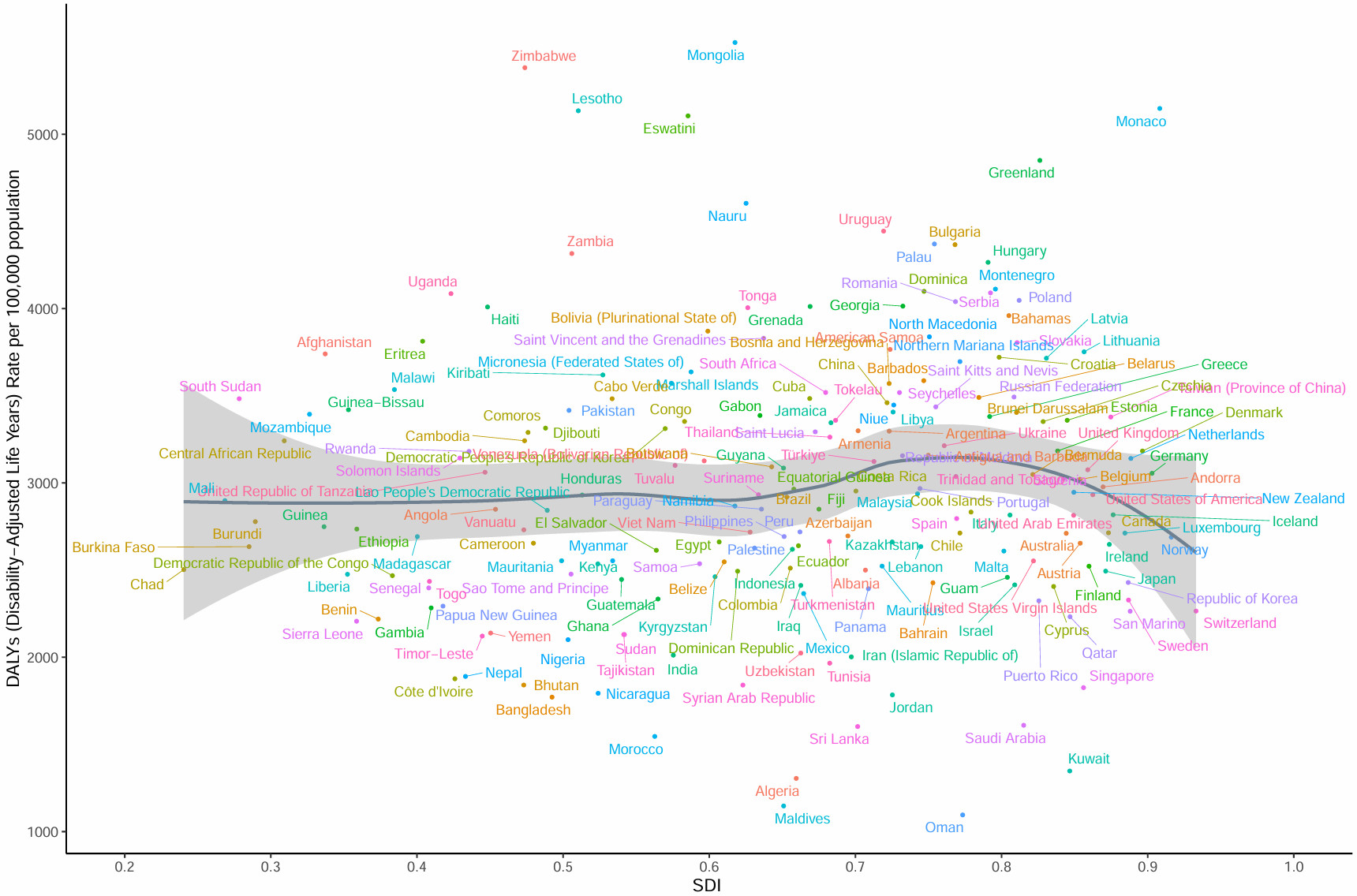


**Figure S14.** Correlations of the sociodemographic index with age-standardised global cancer DALYs for total cancers in 204 countries and territories (r = 0.09, p = 0.18)

**Table S5** Global cancer incidence projections: 1990–2050

| **Value** | **SD** | **Time** | **Group** | **Low_50** | **Up_50** | **Low_60** | **Up_60** | **Low_70** | **Up_70** | **Low_80** | **Up_80** | **Low_95** | **Up_95** |
| --- | --- | --- | --- | --- | --- | --- | --- | --- | --- | --- | --- | --- | --- |
| 13559114.5 | 5744.5 | 1990 | Number | 13555242.7 | 13562986.3 | 13554283.4 | 13563945.6 | 13553163.2 | 13565065.8 | 13551750.1 | 13566478.9 | 13547855.3 | 13570373.7 |
| 13820242.4 | 5780.1 | 1991 | Number | 13816346.6 | 13824138.2 | 13815381.4 | 13825103.4 | 13814254.3 | 13826230.5 | 13812832.4 | 13827652.4 | 13808913.5 | 13831571.3 |
| 14102371.5 | 5820.0 | 1992 | Number | 14098448.8 | 14106294.1 | 14097476.8 | 14107266.1 | 14096341.9 | 14108401.0 | 14094910.2 | 14109832.7 | 14090964.3 | 14113778.6 |
| 14409261.9 | 5864.6 | 1993 | Number | 14405309.2 | 14413214.6 | 14404329.8 | 14414194.0 | 14403186.2 | 14415337.6 | 14401743.5 | 14416780.3 | 14397767.3 | 14420756.5 |
| 14680016.4 | 5900.5 | 1994 | Number | 14676039.5 | 14683993.4 | 14675054.1 | 14684978.8 | 14673903.5 | 14686129.4 | 14672451.9 | 14687580.9 | 14668451.4 | 14691581.4 |
| 14948602.0 | 5936.3 | 1995 | Number | 14944600.9 | 14952603.1 | 14943609.6 | 14953594.4 | 14942452.0 | 14954752.0 | 14940991.6 | 14956212.4 | 14936966.8 | 14960237.2 |
| 15139347.6 | 5955.3 | 1996 | Number | 15135333.7 | 15143361.5 | 15134339.1 | 15144356.0 | 15133177.9 | 15145517.3 | 15131712.8 | 15146982.3 | 15127675.1 | 15151020.0 |
| 15287223.1 | 5964.3 | 1997 | Number | 15283203.2 | 15291243.0 | 15282207.2 | 15292239.1 | 15281044.1 | 15293402.1 | 15279576.9 | 15294869.3 | 15275533.1 | 15298913.1 |
| 15474100.0 | 5980.6 | 1998 | Number | 15470069.0 | 15478130.9 | 15469070.3 | 15479129.7 | 15467904.1 | 15480295.9 | 15466432.8 | 15481767.1 | 15462378.0 | 15485822.0 |
| 15682137.9 | 5999.4 | 1999 | Number | 15678094.3 | 15686181.5 | 15677092.4 | 15687183.4 | 15675922.5 | 15688353.3 | 15674446.6 | 15689829.2 | 15670379.0 | 15693896.8 |
| 15908992.4 | 6021.8 | 2000 | Number | 15904933.7 | 15913051.0 | 15903928.0 | 15914056.7 | 15902753.8 | 15915230.9 | 15901272.4 | 15916712.3 | 15897189.7 | 15920795.0 |
| 16268143.9 | 6069.2 | 2001 | Number | 16264053.2 | 16272234.5 | 16263039.7 | 16273248.1 | 16261856.2 | 16274431.6 | 16260363.2 | 16275924.6 | 16256248.3 | 16280039.5 |
| 16857246.4 | 6158.5 | 2002 | Number | 16853095.6 | 16861397.3 | 16852067.1 | 16862425.7 | 16850866.2 | 16863626.6 | 16849351.2 | 16865141.6 | 16845175.8 | 16869317.1 |
| 17579186.3 | 6270.3 | 2003 | Number | 17574960.2 | 17583412.5 | 17573913.0 | 17584459.6 | 17572690.3 | 17585682.3 | 17571147.8 | 17587224.8 | 17566896.6 | 17591476.0 |
| 18220937.0 | 6362.3 | 2004 | Number | 18216648.8 | 18225225.2 | 18215586.3 | 18226287.7 | 18214345.6 | 18227528.3 | 18212780.5 | 18229093.4 | 18208466.9 | 18233407.1 |
| 18675203.5 | 6417.3 | 2005 | Number | 18670878.2 | 18679528.7 | 18669806.5 | 18680600.4 | 18668555.2 | 18681851.8 | 18666976.5 | 18683430.4 | 18662625.6 | 18687781.3 |
| 18821687.6 | 6415.7 | 2006 | Number | 18817363.4 | 18826011.8 | 18816291.9 | 18827083.2 | 18815040.9 | 18828334.3 | 18813462.6 | 18829912.6 | 18809112.7 | 18834262.4 |
| 19033525.7 | 6424.3 | 2007 | Number | 19029195.7 | 19037855.6 | 19028122.9 | 19038928.5 | 19026870.1 | 19040181.2 | 19025289.8 | 19041761.5 | 19020934.1 | 19046117.2 |
| 19314964.7 | 6445.0 | 2008 | Number | 19310620.8 | 19319308.7 | 19309544.5 | 19320385.0 | 19308287.7 | 19321641.8 | 19306702.2 | 19323227.3 | 19302332.5 | 19327597.0 |
| 19532263.4 | 6453.9 | 2009 | Number | 19527913.5 | 19536613.3 | 19526835.7 | 19537691.1 | 19525577.2 | 19538949.6 | 19523989.5 | 19540537.2 | 19519613.8 | 19544912.9 |
| 19750846.3 | 6462.8 | 2010 | Number | 19746490.3 | 19755202.2 | 19745411.0 | 19756281.5 | 19744150.8 | 19757541.7 | 19742560.9 | 19759131.6 | 19738179.2 | 19763513.4 |
| 19918503.7 | 6461.7 | 2011 | Number | 19914148.5 | 19922858.9 | 19913069.4 | 19923938.0 | 19911809.4 | 19925198.0 | 19910219.8 | 19926787.6 | 19905838.8 | 19931168.6 |
| 20058712.2 | 6454.9 | 2012 | Number | 20054361.5 | 20063062.8 | 20053283.5 | 20064140.8 | 20052024.8 | 20065399.5 | 20050436.9 | 20066987.4 | 20046060.5 | 20071363.8 |
| 20206394.1 | 6450.7 | 2013 | Number | 20202046.2 | 20210741.9 | 20200969.0 | 20211819.1 | 20199711.1 | 20213077.0 | 20198124.2 | 20214663.9 | 20193750.6 | 20219037.5 |
| 20379165.0 | 6450.9 | 2014 | Number | 20374817.1 | 20383512.9 | 20373739.8 | 20384590.2 | 20372481.9 | 20385848.1 | 20370894.9 | 20387435.0 | 20366521.2 | 20391808.7 |
| 20584030.5 | 6458.1 | 2015 | Number | 20579677.8 | 20588383.3 | 20578599.3 | 20589461.8 | 20577339.9 | 20590721.2 | 20575751.2 | 20592309.9 | 20571372.6 | 20596688.5 |
| 20793294.6 | 6464.3 | 2016 | Number | 20788937.6 | 20797651.5 | 20787858.1 | 20798731.1 | 20786597.5 | 20799991.6 | 20785007.3 | 20801581.9 | 20780624.5 | 20805964.7 |
| 20936713.3 | 6459.1 | 2017 | Number | 20932359.9 | 20941066.8 | 20931281.3 | 20942145.4 | 20930021.7 | 20943405.0 | 20928432.8 | 20944993.9 | 20924053.5 | 20949373.2 |
| 21131812.4 | 6462.0 | 2018 | Number | 21127457.1 | 21136167.8 | 21126377.9 | 21137246.9 | 21125117.8 | 21138507.0 | 21123528.2 | 21140096.6 | 21119147.0 | 21144477.8 |
| 21305806.1 | 6461.0 | 2019 | Number | 21301451.4 | 21310160.8 | 21300372.4 | 21311239.8 | 21299112.5 | 21312499.7 | 21297523.0 | 21314089.1 | 21293142.5 | 21318469.7 |
| 21348605.5 | 6443.8 | 2020 | Number | 21344262.4 | 21352948.6 | 21343186.3 | 21354024.7 | 21341929.7 | 21355281.3 | 21340344.6 | 21356866.5 | 21335975.7 | 21361235.4 |
| 21837125.0 | 6495.7 | 2021 | Number | 21832746.9 | 21841503.1 | 21831662.1 | 21842587.9 | 21830395.4 | 21843854.6 | 21828797.5 | 21845452.6 | 21824393.4 | 21849856.7 |
| 22395964.7 | 272325.7 | 2022 | Number | 22212417.2 | 22579512.2 | 22166938.8 | 22624990.6 | 22113835.3 | 22678094.1 | 22046843.2 | 22745086.3 | 21862206.3 | 22929723.1 |
| 22626727.8 | 320050.1 | 2023 | Number | 22411014.0 | 22842441.6 | 22357565.7 | 22895890.0 | 22295155.9 | 22958299.7 | 22216423.6 | 23037032.0 | 21999429.6 | 23254026.0 |
| 22851783.0 | 365017.6 | 2024 | Number | 22605761.2 | 23097804.9 | 22544803.2 | 23158762.9 | 22473624.8 | 23229941.3 | 22383830.5 | 23319735.6 | 22136348.5 | 23567217.6 |
| 23070191.7 | 408255.7 | 2025 | Number | 22795027.4 | 23345356.1 | 22726848.7 | 23413534.8 | 22647238.8 | 23493144.6 | 22546807.9 | 23593575.5 | 22270010.5 | 23870372.9 |
| 23281073.6 | 450412.8 | 2026 | Number | 22977495.3 | 23584651.8 | 22902276.4 | 23659870.8 | 22814445.9 | 23747701.3 | 22703644.3 | 23858502.8 | 22398264.4 | 24163882.7 |
| 23483736.1 | 491918.8 | 2027 | Number | 23152182.8 | 23815289.4 | 23070032.4 | 23897439.9 | 22974108.2 | 23993364.0 | 22853096.2 | 24114376.1 | 22519575.2 | 24447897.0 |
| 23679556.7 | 532784.1 | 2028 | Number | 23320460.2 | 24038653.2 | 23231485.3 | 24127628.2 | 23127592.4 | 24231521.1 | 22996527.5 | 24362586.0 | 22635299.8 | 24723813.6 |
| 23868964.6 | 573126.7 | 2029 | Number | 23482677.3 | 24255252.0 | 23386965.1 | 24350964.2 | 23275205.4 | 24462723.9 | 23134216.2 | 24603713.0 | 22745636.4 | 24992292.9 |
| 24050993.7 | 613085.6 | 2030 | Number | 23637774.1 | 24464213.4 | 23535388.8 | 24566598.7 | 23415837.1 | 24686150.4 | 23265018.1 | 24836969.4 | 22849346.1 | 25252641.4 |
| 24224561.6 | 652786.4 | 2031 | Number | 23784583.5 | 24664539.6 | 23675568.2 | 24773555.0 | 23548274.9 | 24900848.3 | 23387689.4 | 25061433.8 | 22945100.2 | 25504022.9 |
| 24388640.7 | 692359.9 | 2032 | Number | 23921990.1 | 24855291.2 | 23806366.0 | 24970915.3 | 23671355.9 | 25105925.5 | 23501035.3 | 25276246.0 | 23031615.3 | 25745666.0 |
| 24545655.5 | 731713.9 | 2033 | Number | 24052480.4 | 25038830.7 | 23930284.1 | 25161026.9 | 23787599.9 | 25303711.2 | 23607598.3 | 25483712.8 | 23111496.3 | 25979814.8 |
| 24696917.2 | 770870.9 | 2034 | Number | 24177350.3 | 25216484.2 | 24048614.8 | 25345219.6 | 23898295.0 | 25495539.4 | 23708660.8 | 25685173.7 | 23186010.3 | 26207824.1 |
| 24841716.0 | 809868.1 | 2035 | Number | 24295864.9 | 25387567.1 | 24160616.9 | 25522815.1 | 24002692.6 | 25680739.4 | 23803465.1 | 25879966.9 | 23254374.5 | 26429057.5 |
| 24978311.2 | 848718.2 | 2036 | Number | 24406275.2 | 25550347.3 | 24264539.2 | 25692083.2 | 24099039.2 | 25857583.3 | 23890254.5 | 26066368.0 | 23314823.6 | 26641798.9 |
| 25105702.3 | 887497.3 | 2037 | Number | 24507529.1 | 25703875.4 | 24359317.1 | 25852087.5 | 24186255.1 | 26025149.4 | 23967930.8 | 26243473.8 | 23366207.6 | 26845196.9 |
| 25227224.0 | 926150.2 | 2038 | Number | 24602998.7 | 25851449.3 | 24448331.7 | 26006116.3 | 24267732.4 | 26186715.6 | 24039899.4 | 26414548.6 | 23411969.6 | 27042478.4 |
| 25344444.0 | 964695.0 | 2039 | Number | 24694239.6 | 25994648.4 | 24533135.5 | 26155752.5 | 24345020.0 | 26343868.0 | 24107705.0 | 26581183.0 | 23453641.8 | 27235246.2 |
| 25455950.2 | 1003112.9 | 2040 | Number | 24779852.1 | 26132048.3 | 24612332.2 | 26299568.2 | 24416725.2 | 26495175.2 | 24169959.4 | 26741941.0 | 23489848.9 | 27422051.5 |
| 25559289.3 | 1041349.2 | 2041 | Number | 24857420.0 | 26261158.7 | 24683514.7 | 26435064.0 | 24480451.6 | 26638127.1 | 24224279.7 | 26894299.0 | 23518245.0 | 27600333.7 |
| 25653582.2 | 1079461.5 | 2042 | Number | 24926025.1 | 26381139.3 | 24745755.1 | 26561409.4 | 24535260.1 | 26771904.4 | 24269712.5 | 27037451.9 | 23537837.6 | 27769326.9 |
| 25743123.3 | 1117459.3 | 2043 | Number | 24989955.7 | 26496290.9 | 24803340.0 | 26682906.6 | 24585435.5 | 26900811.2 | 24310540.5 | 27175706.1 | 23552903.0 | 27933343.6 |
| 25829177.7 | 1155352.0 | 2044 | Number | 25050470.5 | 26607884.9 | 24857526.7 | 26800828.7 | 24632233.0 | 27026122.3 | 24348016.5 | 27310338.9 | 23564687.8 | 28093667.5 |
| 25909175.8 | 1193051.8 | 2045 | Number | 25105058.9 | 26713292.7 | 24905819.3 | 26912532.4 | 24673174.2 | 27145177.4 | 24379683.4 | 27438668.2 | 23570794.3 | 28247557.3 |
| 25979275.7 | 1230408.9 | 2046 | Number | 25149980.1 | 26808571.3 | 24944501.8 | 27014049.6 | 24704572.1 | 27253979.4 | 24401891.5 | 27556659.9 | 23567674.3 | 28390877.2 |
| 26038800.7 | 1267468.6 | 2047 | Number | 25184526.9 | 26893074.5 | 24972859.6 | 27104741.8 | 24725703.3 | 27351898.2 | 24413906.0 | 27663695.4 | 23554562.3 | 28523039.1 |
| 26093297.0 | 1304329.5 | 2048 | Number | 25214178.9 | 26972415.1 | 24996355.9 | 27190238.1 | 24742011.6 | 27444582.4 | 24421146.6 | 27765447.5 | 23536811.2 | 28649782.9 |
| 26144436.2 | 1341028.0 | 2049 | Number | 25240583.3 | 27048289.1 | 25016631.7 | 27272240.7 | 24755131.2 | 27533741.2 | 24425238.3 | 27863634.1 | 23516021.4 | 28772851.0 |
| 26188672.7 | 1377409.6 | 2050 | Number | 25260298.7 | 27117046.8 | 25030271.3 | 27347074.1 | 24761676.4 | 27615669.0 | 24422833.7 | 27954511.8 | 23488950.0 | 28888395.4 |

**Table S6** Global cancer prevalence projections: 1990–2050

| **Value** | **SD** | **Time** | **Group** | **Low_50** | **Up_50** | **Low_60** | **Up_60** | **Low_70** | **Up_70** | **Low_80** | **Up_80** | **Low_95** | **Up_95** |
| --- | --- | --- | --- | --- | --- | --- | --- | --- | --- | --- | --- | --- | --- |
| 45269082.0 | 10329.3 | 1990 | Number | 45262120.0 | 45276044.0 | 45260395.0 | 45277769.0 | 45258380.8 | 45279783.2 | 45255839.8 | 45282324.2 | 45248836.5 | 45289327.5 |
| 46305702.2 | 10414.6 | 1991 | Number | 46298682.7 | 46312721.6 | 46296943.5 | 46314460.8 | 46294912.7 | 46316491.7 | 46292350.7 | 46319053.6 | 46285289.6 | 46326114.7 |
| 47430436.5 | 10508.5 | 1992 | Number | 47423353.7 | 47437519.2 | 47421598.8 | 47439274.1 | 47419549.6 | 47441323.3 | 47416964.5 | 47443908.4 | 47409839.7 | 47451033.2 |
| 48670276.0 | 10614.2 | 1993 | Number | 48663122.0 | 48677429.9 | 48661349.4 | 48679202.5 | 48659279.7 | 48681272.3 | 48656668.6 | 48683883.4 | 48649472.2 | 48691079.8 |
| 49885857.5 | 10715.0 | 1994 | Number | 49878635.6 | 49893079.4 | 49876846.2 | 49894868.8 | 49874756.8 | 49896958.2 | 49872120.9 | 49899594.1 | 49864856.2 | 49906858.8 |
| 51157307.2 | 10821.3 | 1995 | Number | 51150013.7 | 51164600.8 | 51148206.5 | 51166408.0 | 51146096.4 | 51168518.1 | 51143434.3 | 51171180.2 | 51136097.5 | 51178517.0 |
| 52229553.6 | 10903.8 | 1996 | Number | 52222204.5 | 52236902.8 | 52220383.5 | 52238723.7 | 52218257.3 | 52240850.0 | 52215574.9 | 52243532.3 | 52208182.2 | 52250925.1 |
| 52950180.5 | 10946.7 | 1997 | Number | 52942802.4 | 52957558.6 | 52940974.3 | 52959386.7 | 52938839.7 | 52961521.3 | 52936146.8 | 52964214.2 | 52928724.9 | 52971636.1 |
| 53758211.1 | 10998.4 | 1998 | Number | 53750798.1 | 53765624.0 | 53748961.4 | 53767460.7 | 53746816.7 | 53769605.4 | 53744111.1 | 53772311.0 | 53736654.1 | 53779768.0 |
| 54756541.3 | 11066.2 | 1999 | Number | 54749082.6 | 54763999.9 | 54747234.6 | 54765848.0 | 54745076.7 | 54768005.9 | 54742354.4 | 54770728.2 | 54734851.5 | 54778231.1 |
| 55908102.4 | 11149.6 | 2000 | Number | 55900587.6 | 55915617.2 | 55898725.6 | 55917479.2 | 55896551.4 | 55919653.4 | 55893808.7 | 55922396.2 | 55886249.2 | 55929955.6 |
| 57134648.7 | 11237.5 | 2001 | Number | 57127074.6 | 57142222.8 | 57125198.0 | 57144099.4 | 57123006.7 | 57146290.7 | 57120242.3 | 57149055.1 | 57112623.3 | 57156674.1 |
| 58325042.0 | 11319.0 | 2002 | Number | 58317413.0 | 58332671.0 | 58315522.7 | 58334561.3 | 58313315.5 | 58336768.5 | 58310531.1 | 58339553.0 | 58302856.8 | 58347227.3 |
| 59669178.9 | 11414.5 | 2003 | Number | 59661485.6 | 59676872.3 | 59659579.4 | 59678778.5 | 59657353.6 | 59681004.3 | 59654545.6 | 59683812.3 | 59646806.6 | 59691551.3 |
| 61019498.9 | 11506.1 | 2004 | Number | 61011743.8 | 61027254.0 | 61009822.3 | 61029175.5 | 61007578.6 | 61031419.2 | 61004748.1 | 61034249.7 | 60996947.0 | 61042050.9 |
| 62633062.9 | 11619.3 | 2005 | Number | 62625231.5 | 62640894.3 | 62623291.1 | 62642834.7 | 62621025.3 | 62645100.5 | 62618167.0 | 62647958.8 | 62610289.1 | 62655836.7 |
| 63802661.2 | 11685.3 | 2006 | Number | 63794785.3 | 63810537.0 | 63792833.8 | 63812488.5 | 63790555.2 | 63814767.1 | 63787680.6 | 63817641.7 | 63779758.0 | 63825564.3 |
| 64978850.0 | 11748.4 | 2007 | Number | 64970931.6 | 64986768.5 | 64968969.6 | 64988730.4 | 64966678.7 | 64991021.4 | 64963788.6 | 64993911.5 | 64955823.2 | 65001876.9 |
| 66339458.5 | 11828.1 | 2008 | Number | 66331486.4 | 66347430.6 | 66329511.1 | 66349405.9 | 66327204.6 | 66351712.4 | 66324294.9 | 66354622.1 | 66316275.4 | 66362641.6 |
| 67493880.8 | 11886.9 | 2009 | Number | 67485869.0 | 67501892.6 | 67483883.8 | 67503877.7 | 67481565.9 | 67506195.6 | 67478641.7 | 67509119.8 | 67470582.4 | 67517179.2 |
| 68603384.9 | 11942.2 | 2010 | Number | 68595335.8 | 68611433.9 | 68593341.5 | 68613428.3 | 68591012.8 | 68615757.0 | 68588075.0 | 68618694.8 | 68579978.2 | 68626791.6 |
| 69488868.2 | 11974.0 | 2011 | Number | 69480797.7 | 69496938.6 | 69478798.1 | 69498938.3 | 69476463.2 | 69501273.2 | 69473517.6 | 69504218.8 | 69465399.2 | 69512337.1 |
| 70158477.7 | 11984.9 | 2012 | Number | 70150399.9 | 70166555.6 | 70148398.4 | 70168557.0 | 70146061.3 | 70170894.1 | 70143113.1 | 70173842.4 | 70134987.3 | 70181968.2 |
| 70897610.8 | 12003.0 | 2013 | Number | 70889520.8 | 70905700.8 | 70887516.3 | 70907705.3 | 70885175.7 | 70910045.9 | 70882223.0 | 70912998.6 | 70874085.0 | 70921136.6 |
| 71866896.0 | 12040.4 | 2014 | Number | 71858780.8 | 71875011.2 | 71856770.0 | 71877022.0 | 71854422.1 | 71879369.9 | 71851460.2 | 71882331.8 | 71843296.8 | 71890495.2 |
| 72847906.9 | 12082.1 | 2015 | Number | 72839763.6 | 72856050.2 | 72837745.9 | 72858068.0 | 72835389.8 | 72860424.0 | 72832417.6 | 72863396.2 | 72824226.0 | 72871587.8 |
| 73934341.1 | 12129.5 | 2016 | Number | 73926165.8 | 73942516.4 | 73924140.2 | 73944542.0 | 73921774.9 | 73946907.3 | 73918791.1 | 73949891.2 | 73910567.3 | 73958115.0 |
| 74917292.2 | 12166.1 | 2017 | Number | 74909092.3 | 74925492.2 | 74907060.5 | 74927523.9 | 74904688.2 | 74929896.3 | 74901695.3 | 74932889.1 | 74893446.7 | 74941137.7 |
| 76024105.4 | 12212.3 | 2018 | Number | 76015874.4 | 76032336.5 | 76013834.9 | 76034376.0 | 76011453.5 | 76036757.4 | 76008449.3 | 76039761.6 | 76000169.4 | 76048041.5 |
| 76919699.5 | 12240.4 | 2019 | Number | 76911449.4 | 76927949.5 | 76909405.3 | 76929993.7 | 76907018.4 | 76932380.6 | 76904007.2 | 76935391.7 | 76895708.2 | 76943690.7 |
| 76898476.2 | 12200.1 | 2020 | Number | 76890253.4 | 76906699.1 | 76888215.9 | 76908736.5 | 76885836.9 | 76911115.5 | 76882835.7 | 76914116.7 | 76874564.0 | 76922388.4 |
| 77840521.9 | 12239.9 | 2021 | Number | 77832272.2 | 77848771.6 | 77830228.1 | 77850815.7 | 77827841.3 | 77853202.5 | 77824830.3 | 77856213.5 | 77816531.6 | 77864512.2 |
| 80067555.2 | 794550.9 | 2022 | Number | 79532027.9 | 80603082.5 | 79399337.9 | 80735772.6 | 79244400.4 | 80890710.0 | 79048940.9 | 81086169.5 | 78510235.4 | 81624875.0 |
| 81173567.8 | 951341.8 | 2023 | Number | 80532363.5 | 81814772.2 | 80373489.4 | 81973646.3 | 80187977.7 | 82159157.9 | 79953947.7 | 82393188.0 | 79308937.9 | 83038197.7 |
| 82278300.8 | 1098250.9 | 2024 | Number | 81538079.7 | 83018521.8 | 81354671.8 | 83201929.7 | 81140512.9 | 83416088.7 | 80870343.2 | 83686258.4 | 80125729.1 | 84430872.4 |
| 83376248.1 | 1239670.2 | 2025 | Number | 82540710.4 | 84211785.8 | 82333685.5 | 84418810.7 | 82091949.8 | 84660546.4 | 81786990.9 | 84965505.3 | 80946494.6 | 85806001.7 |
| 84453064.3 | 1378437.9 | 2026 | Number | 83523997.2 | 85382131.5 | 83293798.1 | 85612330.6 | 83025002.7 | 85881126.0 | 82685906.9 | 86220221.7 | 81751326.0 | 87154802.6 |
| 85503598.2 | 1516573.8 | 2027 | Number | 84481427.5 | 86525769.0 | 84228159.7 | 86779036.8 | 83932427.8 | 87074768.7 | 83559350.6 | 87447845.9 | 82531113.6 | 88476082.9 |
| 86544742.8 | 1653291.2 | 2028 | Number | 85430424.6 | 87659061.1 | 85154324.9 | 87935160.7 | 84831933.1 | 88257552.5 | 84425223.5 | 88664262.1 | 83304292.1 | 89785193.6 |
| 87586205.1 | 1788999.9 | 2029 | Number | 86380419.1 | 88791991.0 | 86081656.2 | 89090754.0 | 85732801.2 | 89439609.0 | 85292707.2 | 89879703.0 | 84079765.3 | 91092644.9 |
| 88618992.7 | 1924387.9 | 2030 | Number | 87321955.2 | 89916030.1 | 87000582.4 | 90237402.9 | 86625326.8 | 90612658.6 | 86151927.3 | 91086058.0 | 84847192.3 | 92390793.0 |
| 89626936.4 | 2060113.4 | 2031 | Number | 88238420.0 | 91015452.8 | 87894381.0 | 91359491.7 | 87492658.9 | 91761213.8 | 86985871.0 | 92268001.7 | 85589114.2 | 93664758.6 |
| 90607699.7 | 2197247.3 | 2032 | Number | 89126755.0 | 92088644.4 | 88759814.7 | 92455584.7 | 88331351.5 | 92884047.9 | 87790828.7 | 93424570.7 | 86301095.0 | 94914304.4 |
| 91584031.1 | 2334912.2 | 2033 | Number | 90010300.3 | 93157761.9 | 89620369.9 | 93547692.2 | 89165062.1 | 94003000.1 | 88590673.7 | 94577388.5 | 87007603.2 | 96160458.9 |
| 92566237.0 | 2473132.6 | 2034 | Number | 90899345.6 | 94233128.3 | 90486332.5 | 94646141.5 | 90004071.6 | 95128402.3 | 89395681.0 | 95736792.9 | 87718897.2 | 97413576.8 |
| 93540266.1 | 2612015.3 | 2035 | Number | 91779767.8 | 95300764.4 | 91343561.2 | 95736971.0 | 90834218.2 | 96246313.9 | 90191662.5 | 96888869.7 | 88420716.1 | 98659816.1 |
| 94486934.6 | 2751704.4 | 2036 | Number | 92632285.8 | 96341583.3 | 92172751.1 | 96801118.0 | 91636168.8 | 97337700.3 | 90959249.5 | 98014619.6 | 89093593.9 | 99880275.2 |
| 95407936.8 | 2893238.6 | 2037 | Number | 93457894.0 | 97357979.6 | 92974723.1 | 97841150.4 | 92410541.6 | 98405331.9 | 91698804.9 | 99117068.6 | 89737189.2 | 101078684.4 |
| 96332482.1 | 3036064.3 | 2038 | Number | 94286174.8 | 98378789.4 | 93779152.0 | 98885812.2 | 93187119.5 | 99477844.7 | 92440247.7 | 100224716.5 | 90381796.1 | 102283168.1 |
| 97268412.2 | 3180071.2 | 2039 | Number | 95125044.3 | 99411780.2 | 94593972.4 | 99942852.1 | 93973858.5 | 100562965.9 | 93191561.0 | 101345263.4 | 91035472.8 | 103501351.7 |
| 98193336.3 | 3324930.6 | 2040 | Number | 95952333.0 | 100434339.5 | 95397069.6 | 100989602.9 | 94748708.1 | 101637964.4 | 93930775.2 | 102455897.3 | 91676472.2 | 104710200.3 |
| 99084025.1 | 3470455.0 | 2041 | Number | 96744938.5 | 101423111.8 | 96165372.5 | 102002677.8 | 95488633.8 | 102679416.5 | 94634901.8 | 103533148.4 | 92281933.4 | 105886116.9 |
| 99946983.0 | 3617826.4 | 2042 | Number | 97508568.0 | 102385398.0 | 96904391.0 | 102989575.0 | 96198914.8 | 103695051.1 | 95308929.6 | 104585036.4 | 92856043.3 | 107037922.7 |
| 100816653.3 | 3766812.0 | 2043 | Number | 98277822.0 | 103355484.6 | 97648764.4 | 103984542.2 | 96914236.0 | 104719070.5 | 95987600.3 | 105645706.3 | 93433701.7 | 108199604.8 |
| 101697006.6 | 3917121.0 | 2044 | Number | 99056867.0 | 104337146.1 | 98402707.8 | 104991305.3 | 97638869.2 | 105755143.9 | 96675257.4 | 106718755.7 | 94019449.4 | 109374563.8 |
| 102555847.0 | 4067973.7 | 2045 | Number | 99814032.7 | 105297661.3 | 99134681.1 | 105977012.9 | 98341426.3 | 106770267.8 | 97340704.7 | 107770989.3 | 94582618.6 | 110529075.4 |
| 103364363.6 | 4218884.6 | 2046 | Number | 100520835.4 | 106207891.9 | 99816281.7 | 106912445.6 | 98993599.2 | 107735128.1 | 97955753.6 | 108772973.7 | 95095349.8 | 111633377.5 |
| 104131832.8 | 4371119.4 | 2047 | Number | 101185698.3 | 107077967.3 | 100455721.4 | 107807944.3 | 99603353.1 | 108660312.5 | 98528057.7 | 109735607.9 | 95564438.8 | 112699226.9 |
| 104898986.4 | 4524785.0 | 2048 | Number | 101849281.3 | 107948691.5 | 101093642.2 | 108704330.6 | 100211309.2 | 109586663.7 | 99098212.1 | 110699760.8 | 96030407.8 | 113767565.1 |
| 105669528.9 | 4679493.6 | 2049 | Number | 102515550.2 | 108823507.6 | 101734074.8 | 109604983.0 | 100821573.5 | 110517484.2 | 99670418.1 | 111668639.7 | 96497721.4 | 114841336.3 |
| 106405874.9 | 4834163.0 | 2050 | Number | 103147649.1 | 109664100.8 | 102340343.8 | 110471406.1 | 101397682.0 | 111414067.9 | 100208477.9 | 112603272.0 | 96930915.4 | 115880834.5 |

**Table S7** Global cancer deaths projections: 1990–2050

| **Value** | **SD** | **Time** | **Group** | **Low_50** | **Up_50** | **Low_60** | **Up_60** | **Low_70** | **Up_70** | **Low_80** | **Up_80** | **Low_95** | **Up_95** |
| --- | --- | --- | --- | --- | --- | --- | --- | --- | --- | --- | --- | --- | --- |
| 7924708.7 | 4423.8 | 1990 | Number | 7921727.1 | 7927690.4 | 7920988.3 | 7928429.2 | 7920125.7 | 7929291.8 | 7919037.4 | 7930380.1 | 7916038.1 | 7933379.4 |
| 8009813.5 | 4430.4 | 1991 | Number | 8006827.4 | 8012799.6 | 8006087.5 | 8013539.4 | 8005223.6 | 8014403.4 | 8004133.7 | 8015493.2 | 8001129.9 | 8018497.1 |
| 8109748.5 | 4443.4 | 1992 | Number | 8106753.6 | 8112743.4 | 8106011.6 | 8113485.4 | 8105145.1 | 8114351.9 | 8104052.0 | 8115444.9 | 8101039.4 | 8118457.6 |
| 8219031.6 | 4459.2 | 1993 | Number | 8216026.1 | 8222037.1 | 8215281.4 | 8222781.7 | 8214411.8 | 8223651.3 | 8213314.9 | 8224748.3 | 8210291.6 | 8227771.6 |
| 8299623.2 | 4466.7 | 1994 | Number | 8296612.7 | 8302633.8 | 8295866.7 | 8303379.7 | 8294995.7 | 8304250.8 | 8293896.9 | 8305349.6 | 8290868.5 | 8308378.0 |
| 8373934.4 | 4473.3 | 1995 | Number | 8370919.4 | 8376949.4 | 8370172.3 | 8377696.5 | 8369300.0 | 8378568.8 | 8368199.6 | 8379669.2 | 8365166.6 | 8382702.2 |
| 8390396.3 | 4464.2 | 1996 | Number | 8387387.5 | 8393405.2 | 8386642.0 | 8394150.7 | 8385771.5 | 8395021.2 | 8384673.3 | 8396119.4 | 8381646.6 | 8399146.1 |
| 8402202.4 | 4452.7 | 1997 | Number | 8399201.3 | 8405203.5 | 8398457.7 | 8405947.1 | 8397589.4 | 8406815.4 | 8396494.1 | 8407910.8 | 8393475.1 | 8410929.7 |
| 8454205.1 | 4451.7 | 1998 | Number | 8451204.7 | 8457205.6 | 8450461.2 | 8457949.0 | 8449593.2 | 8458817.1 | 8448498.0 | 8459912.3 | 8445479.8 | 8462930.5 |
| 8522206.2 | 4453.8 | 1999 | Number | 8519204.3 | 8525208.0 | 8518460.5 | 8525951.8 | 8517592.0 | 8526820.3 | 8516496.4 | 8527916.0 | 8513476.7 | 8530935.7 |
| 8607812.7 | 4460.3 | 2000 | Number | 8604806.5 | 8610818.9 | 8604061.6 | 8611563.8 | 8603191.8 | 8612433.5 | 8602094.6 | 8613530.7 | 8599070.6 | 8616554.8 |
| 8649950.6 | 4455.3 | 2001 | Number | 8646947.8 | 8652953.5 | 8646203.7 | 8653697.5 | 8645334.9 | 8654566.3 | 8644238.9 | 8655662.3 | 8641218.3 | 8658682.9 |
| 8687748.0 | 4448.6 | 2002 | Number | 8684749.7 | 8690746.4 | 8684006.8 | 8691489.3 | 8683139.3 | 8692356.8 | 8682044.9 | 8693451.2 | 8679028.8 | 8696467.3 |
| 8749416.5 | 4449.1 | 2003 | Number | 8746417.8 | 8752415.2 | 8745674.8 | 8753158.2 | 8744807.3 | 8754025.8 | 8743712.8 | 8755120.2 | 8740696.3 | 8758136.7 |
| 8783561.2 | 4441.0 | 2004 | Number | 8780568.0 | 8786554.4 | 8779826.3 | 8787296.1 | 8778960.3 | 8788162.1 | 8777867.9 | 8789254.5 | 8774856.9 | 8792265.5 |
| 8788636.9 | 4424.7 | 2005 | Number | 8785654.6 | 8791619.2 | 8784915.7 | 8792358.1 | 8784052.9 | 8793221.0 | 8782964.4 | 8794309.4 | 8779964.4 | 8797309.4 |
| 8701960.6 | 4384.6 | 2006 | Number | 8699005.4 | 8704915.8 | 8698273.2 | 8705648.0 | 8697418.2 | 8706503.0 | 8696339.6 | 8707581.6 | 8693366.8 | 8710554.3 |
| 8704356.4 | 4366.4 | 2007 | Number | 8701413.4 | 8707299.3 | 8700684.2 | 8708028.5 | 8699832.8 | 8708879.9 | 8698758.7 | 8709954.1 | 8695798.3 | 8712914.5 |
| 8775871.8 | 4366.0 | 2008 | Number | 8772929.2 | 8778814.5 | 8772200.0 | 8779543.6 | 8771348.7 | 8780395.0 | 8770274.6 | 8781469.1 | 8767314.5 | 8784429.2 |
| 8821840.9 | 4358.5 | 2009 | Number | 8818903.3 | 8824778.5 | 8818175.4 | 8825506.4 | 8817325.5 | 8826356.3 | 8816253.3 | 8827428.5 | 8813298.3 | 8830383.5 |
| 8870763.0 | 4351.3 | 2010 | Number | 8867830.2 | 8873695.8 | 8867103.6 | 8874422.5 | 8866255.1 | 8875271.0 | 8865184.6 | 8876341.4 | 8862234.4 | 8879291.6 |
| 8894487.4 | 4337.2 | 2011 | Number | 8891564.1 | 8897410.6 | 8890839.8 | 8898134.9 | 8889994.0 | 8898980.7 | 8888927.1 | 8900047.6 | 8885986.4 | 8902988.3 |
| 8901477.7 | 4318.1 | 2012 | Number | 8898567.2 | 8904388.1 | 8897846.1 | 8905109.2 | 8897004.1 | 8905951.2 | 8895941.8 | 8907013.5 | 8893014.1 | 8909941.2 |
| 8909593.9 | 4300.9 | 2013 | Number | 8906695.1 | 8912492.7 | 8905976.9 | 8913210.9 | 8905138.2 | 8914049.6 | 8904080.2 | 8915107.6 | 8901164.2 | 8918023.6 |
| 8937037.7 | 4288.9 | 2014 | Number | 8934146.9 | 8939928.4 | 8933430.7 | 8940644.6 | 8932594.4 | 8941481.0 | 8931539.3 | 8942536.0 | 8928631.4 | 8945443.9 |
| 8997165.8 | 4285.6 | 2015 | Number | 8994277.3 | 9000054.3 | 8993561.6 | 9000769.9 | 8992725.9 | 9001605.6 | 8991671.6 | 9002659.9 | 8988766.0 | 9005565.5 |
| 9078399.4 | 4286.5 | 2016 | Number | 9075510.3 | 9081288.5 | 9074794.5 | 9082004.3 | 9073958.6 | 9082840.2 | 9072904.1 | 9083894.7 | 9069997.9 | 9086800.9 |
| 9096477.4 | 4271.5 | 2017 | Number | 9093598.4 | 9099356.4 | 9092885.0 | 9100069.7 | 9092052.1 | 9100902.7 | 9091001.3 | 9101953.5 | 9088105.2 | 9104849.6 |
| 9147181.2 | 4264.3 | 2018 | Number | 9144307.0 | 9150055.3 | 9143594.9 | 9150767.4 | 9142763.3 | 9151599.0 | 9141714.3 | 9152648.0 | 9138823.1 | 9155539.2 |
| 9188539.3 | 4254.6 | 2019 | Number | 9185671.7 | 9191406.9 | 9184961.2 | 9192117.4 | 9184131.5 | 9192947.1 | 9183084.9 | 9193993.7 | 9180200.3 | 9196878.3 |
| 9172580.5 | 4234.4 | 2020 | Number | 9169726.5 | 9175434.4 | 9169019.4 | 9176141.6 | 9168193.7 | 9176967.3 | 9167152.0 | 9178008.9 | 9164281.1 | 9180879.8 |
| 9207230.4 | 4228.6 | 2021 | Number | 9204380.3 | 9210080.5 | 9203674.1 | 9210786.7 | 9202849.5 | 9211611.3 | 9201809.3 | 9212651.5 | 9198942.3 | 9215518.5 |
| 9284231.6 | 89747.5 | 2022 | Number | 9223741.8 | 9344721.5 | 9208753.9 | 9359709.3 | 9191253.2 | 9377210.1 | 9169175.3 | 9399288.0 | 9108326.4 | 9460136.8 |
| 9263838.0 | 105568.9 | 2023 | Number | 9192684.6 | 9334991.5 | 9175054.5 | 9352621.5 | 9154468.6 | 9373207.5 | 9128498.6 | 9399177.4 | 9056922.9 | 9470753.2 |
| 9239821.0 | 119832.0 | 2024 | Number | 9159054.2 | 9320587.8 | 9139042.3 | 9340599.7 | 9115675.0 | 9363967.0 | 9086196.3 | 9393445.6 | 9004950.2 | 9474691.8 |
| 9211939.7 | 133043.6 | 2025 | Number | 9122268.4 | 9301611.1 | 9100050.1 | 9323829.4 | 9074106.6 | 9349772.9 | 9041377.9 | 9382501.6 | 8951174.3 | 9472705.2 |
| 9181216.3 | 145529.1 | 2026 | Number | 9083129.7 | 9279302.9 | 9058826.3 | 9303606.2 | 9030448.2 | 9331984.4 | 8994648.0 | 9367784.6 | 8895979.3 | 9466453.3 |
| 9148026.0 | 157480.2 | 2027 | Number | 9041884.3 | 9254167.7 | 9015585.2 | 9280466.9 | 8984876.5 | 9311175.5 | 8946136.4 | 9349915.7 | 8839364.8 | 9456687.2 |
| 9112480.3 | 168868.3 | 2028 | Number | 8998663.1 | 9226297.5 | 8970462.1 | 9254498.5 | 8937532.8 | 9287427.8 | 8895991.2 | 9328969.4 | 8781498.5 | 9443462.0 |
| 9074013.9 | 179743.2 | 2029 | Number | 8952867.0 | 9195160.8 | 8922849.9 | 9225177.9 | 8887799.9 | 9260227.8 | 8843583.1 | 9304444.6 | 8721717.2 | 9426310.5 |
| 9032439.8 | 190201.2 | 2030 | Number | 8904244.2 | 9160635.4 | 8872480.6 | 9192399.0 | 8835391.4 | 9229488.2 | 8788601.9 | 9276277.7 | 8659645.5 | 9405234.1 |
| 8989005.8 | 200345.2 | 2031 | Number | 8853973.1 | 9124038.4 | 8820515.5 | 9157496.1 | 8781448.2 | 9196563.4 | 8732163.3 | 9245848.3 | 8596329.3 | 9381682.3 |
| 8943927.6 | 210237.1 | 2032 | Number | 8802227.8 | 9085627.4 | 8767118.2 | 9120737.0 | 8726121.9 | 9161733.2 | 8674403.6 | 9213451.6 | 8531862.9 | 9355992.3 |
| 8897123.0 | 219793.7 | 2033 | Number | 8748982.0 | 9045263.9 | 8712276.4 | 9081969.5 | 8669416.7 | 9124829.3 | 8615347.4 | 9178898.5 | 8466327.3 | 9327918.7 |
| 8847965.1 | 229009.5 | 2034 | Number | 8693612.8 | 9002317.5 | 8655368.2 | 9040562.1 | 8610711.3 | 9085219.0 | 8554375.0 | 9141555.3 | 8399106.6 | 9296823.7 |
| 8796271.9 | 237931.2 | 2035 | Number | 8635906.2 | 8956637.5 | 8596171.7 | 8996372.0 | 8549775.1 | 9042768.6 | 8491244.0 | 9101299.7 | 8329926.6 | 9262617.1 |
| 8743113.4 | 246623.5 | 2036 | Number | 8576889.1 | 8909337.6 | 8535703.0 | 8950523.8 | 8487611.4 | 8998615.4 | 8426942.0 | 9059284.7 | 8259731.2 | 9226495.5 |
| 8688738.5 | 255127.9 | 2037 | Number | 8516782.3 | 8860694.7 | 8474176.0 | 8903301.0 | 8424426.0 | 8953051.0 | 8361664.6 | 9015812.4 | 8188687.9 | 9188789.1 |
| 8632970.6 | 263360.2 | 2038 | Number | 8455465.8 | 8810475.3 | 8411484.6 | 8854456.5 | 8360129.4 | 8905811.7 | 8295342.8 | 8970598.3 | 8116784.6 | 9149156.5 |
| 8575173.7 | 271301.6 | 2039 | Number | 8392316.5 | 8758031.0 | 8347009.1 | 8803338.3 | 8294105.3 | 8856242.1 | 8227365.1 | 8922982.3 | 8043422.7 | 9106924.8 |
| 8514990.8 | 278976.6 | 2040 | Number | 8326960.6 | 8703021.0 | 8280371.5 | 8749610.1 | 8225971.1 | 8804010.6 | 8157342.8 | 8872638.8 | 7968196.7 | 9061784.9 |
| 8453426.7 | 286438.6 | 2041 | Number | 8260367.1 | 8646486.3 | 8212531.9 | 8694321.6 | 8156676.3 | 8750177.1 | 8086212.5 | 8820641.0 | 7892007.1 | 9014846.3 |
| 8391005.7 | 293734.3 | 2042 | Number | 8193028.8 | 8588982.6 | 8143975.2 | 8638036.2 | 8086697.0 | 8695314.4 | 8014438.3 | 8767573.1 | 7815286.5 | 8966724.9 |
| 8327851.4 | 300799.2 | 2043 | Number | 8125112.7 | 8530590.1 | 8074879.2 | 8580823.6 | 8016223.4 | 8639479.4 | 7942226.8 | 8713476.0 | 7738284.9 | 8917417.9 |
| 8263263.4 | 307605.9 | 2044 | Number | 8055937.0 | 8470589.8 | 8004566.9 | 8521959.9 | 7944583.7 | 8581943.1 | 7868912.7 | 8657614.1 | 7660355.9 | 8866170.9 |
| 8196477.1 | 314150.8 | 2045 | Number | 7984739.4 | 8408214.7 | 7932276.2 | 8460677.9 | 7871016.8 | 8521937.3 | 7793735.7 | 8599218.4 | 7580741.4 | 8812212.7 |
| 8127865.1 | 320459.3 | 2046 | Number | 7911875.5 | 8343854.7 | 7858358.8 | 8397371.4 | 7795869.2 | 8459861.0 | 7717036.3 | 8538693.9 | 7499764.8 | 8755965.4 |
| 8058075.9 | 326583.0 | 2047 | Number | 7837958.9 | 8278192.8 | 7783419.6 | 8332732.2 | 7719735.9 | 8396415.9 | 7639396.5 | 8476755.3 | 7417973.2 | 8698178.6 |
| 7987595.3 | 332481.9 | 2048 | Number | 7763502.5 | 8211688.2 | 7707978.1 | 8267212.6 | 7643144.1 | 8332046.6 | 7561353.5 | 8413837.2 | 7335930.8 | 8639259.9 |
| 7916001.0 | 338134.0 | 2049 | Number | 7688098.7 | 8143903.4 | 7631630.3 | 8200371.8 | 7565694.2 | 8266307.9 | 7482513.2 | 8349488.9 | 7253258.4 | 8578743.7 |
| 7842214.8 | 343511.0 | 2050 | Number | 7610688.4 | 8073741.2 | 7553322.0 | 8131107.5 | 7486337.4 | 8198092.2 | 7401833.7 | 8282595.9 | 7168933.2 | 8515496.4 |

**Table S8** Global projections of cancer-related DALYs: 1990–2050

| **Value** | **SD** | **Time** | **Group** | **Low_50** | **Up_50** | **Low_60** | **Up_60** | **Low_70** | **Up_70** | **Low_80** | **Up_80** | **Low_95** | **Up_95** |
| --- | --- | --- | --- | --- | --- | --- | --- | --- | --- | --- | --- | --- | --- |
| 211593087.1 | 22090.0 | 1990 | Number | 211578198.5 | 211607975.8 | 211574509.4 | 211611664.8 | 211570201.9 | 211615972.4 | 211564767.7 | 211621406.5 | 211549790.7 | 211636383.5 |
| 213616161.3 | 22146.8 | 1991 | Number | 213601234.4 | 213631088.2 | 213597535.9 | 213634786.8 | 213593217.2 | 213639105.4 | 213587769.1 | 213644553.5 | 213572753.6 | 213659569.0 |
| 215873818.8 | 22215.1 | 1992 | Number | 215858845.9 | 215888791.8 | 215855136.0 | 215892501.7 | 215850804.0 | 215896833.7 | 215845339.1 | 215902298.6 | 215830277.3 | 215917360.4 |
| 218398319.8 | 22298.2 | 1993 | Number | 218383290.8 | 218413348.7 | 218379567.0 | 218417072.5 | 218375218.8 | 218421420.7 | 218369733.5 | 218426906.0 | 218354615.3 | 218442024.2 |
| 220129956.8 | 22337.9 | 1994 | Number | 220114901.1 | 220145012.5 | 220111170.7 | 220148742.9 | 220106814.8 | 220153098.8 | 220101319.7 | 220158593.9 | 220086174.6 | 220173739.0 |
| 221544838.2 | 22365.1 | 1995 | Number | 221529764.2 | 221559912.3 | 221526029.2 | 221563647.2 | 221521668.0 | 221568008.4 | 221516166.2 | 221573510.2 | 221501002.7 | 221588673.7 |
| 221466541.6 | 22312.1 | 1996 | Number | 221451503.3 | 221481580.0 | 221447777.2 | 221485306.1 | 221443426.3 | 221489656.9 | 221437937.6 | 221495145.7 | 221422810.0 | 221510273.3 |
| 221269158.6 | 22247.3 | 1997 | Number | 221254164.0 | 221284153.3 | 221250448.7 | 221287868.6 | 221246110.4 | 221292206.8 | 221240637.6 | 221297679.7 | 221225553.9 | 221312763.3 |
| 221975714.6 | 22229.0 | 1998 | Number | 221960732.2 | 221990696.9 | 221957020.0 | 221994409.2 | 221952685.3 | 221998743.9 | 221947217.0 | 222004212.2 | 221932145.7 | 222019283.5 |
| 223368921.4 | 22239.9 | 1999 | Number | 223353931.7 | 223383911.0 | 223350217.6 | 223387625.1 | 223345880.8 | 223391961.9 | 223340409.8 | 223397432.9 | 223325331.2 | 223412511.5 |
| 225083122.0 | 22269.2 | 2000 | Number | 225068112.5 | 225098131.4 | 225064393.6 | 225101850.4 | 225060051.1 | 225106192.9 | 225054572.9 | 225111671.1 | 225039474.3 | 225126769.6 |
| 225475482.1 | 22229.0 | 2001 | Number | 225460499.7 | 225490464.4 | 225456787.5 | 225494176.7 | 225452452.8 | 225498511.3 | 225446984.5 | 225503979.7 | 225431913.2 | 225519050.9 |
| 225805445.5 | 22180.8 | 2002 | Number | 225790495.7 | 225820395.4 | 225786791.5 | 225824099.6 | 225782466.2 | 225828424.8 | 225777009.7 | 225833881.3 | 225761971.1 | 225848919.9 |
| 226543723.1 | 22155.6 | 2003 | Number | 226528790.3 | 226558656.0 | 226525090.3 | 226562356.0 | 226520769.9 | 226566676.3 | 226515319.7 | 226572126.6 | 226500298.2 | 226587148.1 |
| 226898041.8 | 22106.4 | 2004 | Number | 226883142.1 | 226912941.5 | 226879450.4 | 226916633.3 | 226875139.6 | 226920944.0 | 226869701.4 | 226926382.2 | 226854713.3 | 226941370.3 |
| 226797585.7 | 22036.2 | 2005 | Number | 226782733.3 | 226812438.1 | 226779053.3 | 226816118.1 | 226774756.3 | 226820415.2 | 226769335.4 | 226825836.1 | 226754394.8 | 226840776.6 |
| 224465497.6 | 21851.7 | 2006 | Number | 224450769.6 | 224480225.7 | 224447120.3 | 224483874.9 | 224442859.3 | 224488136.0 | 224437483.7 | 224493511.6 | 224422668.3 | 224508327.0 |
| 224061200.6 | 21758.6 | 2007 | Number | 224046535.2 | 224075865.9 | 224042901.6 | 224079499.6 | 224038658.6 | 224083742.5 | 224033306.0 | 224089095.1 | 224018553.7 | 224103847.5 |
| 225370741.1 | 21751.8 | 2008 | Number | 225356080.4 | 225385401.8 | 225352447.8 | 225389034.4 | 225348206.2 | 225393276.0 | 225342855.3 | 225398626.9 | 225328107.6 | 225413374.6 |
| 225976802.9 | 21709.4 | 2009 | Number | 225962170.8 | 225991435.0 | 225958545.3 | 225995060.5 | 225954312.0 | 225999293.8 | 225948971.5 | 226004634.3 | 225934252.5 | 226019353.2 |
| 226702524.3 | 21673.6 | 2010 | Number | 226687916.3 | 226717132.3 | 226684296.8 | 226720751.8 | 226680070.4 | 226724978.1 | 226674738.7 | 226730309.8 | 226660044.0 | 226745004.5 |
| 226871711.4 | 21607.3 | 2011 | Number | 226857148.1 | 226886274.7 | 226853539.7 | 226889883.1 | 226849326.2 | 226894096.5 | 226844010.9 | 226899411.9 | 226829361.1 | 226914061.6 |
| 226930580.3 | 21531.9 | 2012 | Number | 226916067.8 | 226945092.8 | 226912472.0 | 226948688.6 | 226908273.2 | 226952887.3 | 226902976.4 | 226958184.2 | 226888377.7 | 226972782.8 |
| 226917860.4 | 21456.9 | 2013 | Number | 226903398.4 | 226932322.3 | 226899815.1 | 226935905.6 | 226895631.0 | 226940089.7 | 226890352.6 | 226945368.1 | 226875804.8 | 226959915.9 |
| 227326217.1 | 21403.7 | 2014 | Number | 227311791.0 | 227340643.2 | 227308216.6 | 227344217.6 | 227304042.8 | 227348391.3 | 227298777.5 | 227353656.6 | 227284265.8 | 227368168.3 |
| 228593415.9 | 21395.4 | 2015 | Number | 228578995.4 | 228607836.4 | 228575422.4 | 228611409.5 | 228571250.3 | 228615581.6 | 228565987.0 | 228620844.8 | 228551481.0 | 228635350.9 |
| 230328032.9 | 21407.6 | 2016 | Number | 230313604.2 | 230342461.6 | 230310029.2 | 230346036.7 | 230305854.7 | 230350211.2 | 230300588.4 | 230355477.4 | 230286074.1 | 230369991.8 |
| 230787820.7 | 21358.2 | 2017 | Number | 230773425.3 | 230802216.1 | 230769858.4 | 230805782.9 | 230765693.6 | 230809947.8 | 230760439.5 | 230815201.9 | 230745958.6 | 230829682.7 |
| 231874678.2 | 21337.8 | 2018 | Number | 231860296.6 | 231889059.9 | 231856733.1 | 231892623.3 | 231852572.3 | 231896784.1 | 231847323.2 | 231902033.2 | 231832856.2 | 231916500.2 |
| 232685121.3 | 21304.7 | 2019 | Number | 232670762.0 | 232699480.7 | 232667204.1 | 232703038.5 | 232663049.7 | 232707193.0 | 232657808.8 | 232712433.9 | 232643364.2 | 232726878.5 |
| 231859713.4 | 21203.6 | 2020 | Number | 231845422.1 | 231874004.6 | 231841881.1 | 231877545.6 | 231837746.4 | 231881680.3 | 231832530.3 | 231886896.4 | 231818154.3 | 231901272.4 |
| 232723873.4 | 21187.4 | 2021 | Number | 232709593.1 | 232738153.7 | 232706054.8 | 232741692.0 | 232701923.3 | 232745823.5 | 232696711.2 | 232751035.6 | 232682346.1 | 232765400.6 |
| 234700406.6 | 2220770.7 | 2022 | Number | 233203607.2 | 236197206.1 | 232832738.5 | 236568074.8 | 232399688.2 | 237001125.0 | 231853378.7 | 237547434.6 | 230347696.2 | 239053117.1 |
| 234076028.8 | 2623103.3 | 2023 | Number | 232308057.2 | 235844000.5 | 231869998.9 | 236282058.7 | 231358493.8 | 236793563.9 | 230713210.4 | 237438847.3 | 228934746.3 | 239217311.4 |
| 233370946.5 | 2984638.5 | 2024 | Number | 231359300.1 | 235382592.8 | 230860865.5 | 235881027.4 | 230278861.0 | 236463031.9 | 229544640.0 | 237197253.0 | 227521055.1 | 239220837.9 |
| 232575446.1 | 3318859.3 | 2025 | Number | 230338534.9 | 234812357.3 | 229784285.4 | 235366606.8 | 229137107.8 | 236013784.4 | 228320668.4 | 236830223.8 | 226070481.8 | 239080410.4 |
| 231718516.9 | 3634248.7 | 2026 | Number | 229269033.3 | 234168000.6 | 228662113.7 | 234774920.1 | 227953435.2 | 235483598.6 | 227059410.0 | 236377623.8 | 224595389.4 | 238841644.4 |
| 230812476.4 | 3935845.2 | 2027 | Number | 228159716.7 | 233465236.1 | 227502430.5 | 234122522.2 | 226734940.7 | 234890012.1 | 225766722.8 | 235858230.0 | 223098219.7 | 238526733.1 |
| 229859728.8 | 4223418.4 | 2028 | Number | 227013144.8 | 232706312.8 | 226307833.9 | 233411623.6 | 225484267.3 | 234235190.2 | 224445306.4 | 235274151.1 | 221581828.7 | 238137628.8 |
| 228843708.2 | 4498387.9 | 2029 | Number | 225811794.8 | 231875621.7 | 225060564.0 | 232626852.4 | 224183378.4 | 233504038.1 | 223076775.0 | 234610641.5 | 220026868.0 | 237660548.5 |
| 227751652.4 | 4762918.8 | 2030 | Number | 224541445.2 | 230961859.7 | 223746037.7 | 231757267.2 | 222817268.5 | 232686036.3 | 221645590.5 | 233857714.4 | 218416331.5 | 237086973.3 |
| 226608198.2 | 5019295.1 | 2031 | Number | 223225193.3 | 229991203.0 | 222386971.0 | 230829425.3 | 221408208.4 | 231808187.9 | 220173461.8 | 233042934.5 | 216770379.8 | 236446016.5 |
| 225427905.4 | 5269292.5 | 2032 | Number | 221876402.3 | 228979408.6 | 220996430.4 | 229859380.4 | 219968918.4 | 230886892.4 | 218672672.5 | 232183138.4 | 215100092.2 | 235755718.7 |
| 224215170.6 | 5511360.4 | 2033 | Number | 220500513.7 | 227929827.5 | 219580116.5 | 228850224.7 | 218505401.3 | 229924939.9 | 217149606.6 | 231280734.6 | 213412904.3 | 235017436.9 |
| 222955880.0 | 5745475.1 | 2034 | Number | 219083429.8 | 226828330.2 | 218123935.4 | 227787824.5 | 217003567.8 | 228908192.2 | 215590180.9 | 230321579.0 | 211694748.8 | 234217011.1 |
| 221632013.2 | 5972282.2 | 2035 | Number | 217606695.0 | 225657331.4 | 216609323.8 | 226654702.5 | 215444728.8 | 227819297.5 | 213975547.4 | 229288479.0 | 209926340.1 | 233337686.3 |
| 220254837.7 | 6192792.7 | 2036 | Number | 216080895.4 | 224428780.0 | 215046699.0 | 225462976.4 | 213839104.4 | 226670571.0 | 212315677.4 | 228193998.0 | 208116963.9 | 232392711.5 |
| 218839862.7 | 6408323.5 | 2037 | Number | 214520652.6 | 223159072.8 | 213450462.6 | 224229262.8 | 212200839.5 | 225478885.9 | 210624391.9 | 227055333.5 | 206279548.6 | 231400176.8 |
| 217398584.8 | 6617573.8 | 2038 | Number | 212938340.0 | 221858829.5 | 211833205.2 | 222963964.3 | 210542778.3 | 224254391.2 | 208914855.1 | 225882314.4 | 204428140.1 | 230369029.4 |
| 215919286.9 | 6820207.8 | 2039 | Number | 211322466.9 | 220516107.0 | 210183492.2 | 221655081.7 | 208853551.6 | 222985022.3 | 207175780.5 | 224662793.4 | 202551679.6 | 229286894.3 |
| 214377262.5 | 7016135.3 | 2040 | Number | 209648387.3 | 219106137.7 | 208476692.7 | 220277832.3 | 207108546.3 | 221645978.7 | 205382577.0 | 223371948.0 | 200625637.3 | 228128887.7 |
| 212767977.9 | 7205534.0 | 2041 | Number | 207911447.9 | 217624507.8 | 206708123.8 | 218827832.0 | 205303044.6 | 220232911.1 | 203530483.3 | 222005472.5 | 198645131.2 | 226890824.5 |
| 211108174.7 | 7389650.0 | 2042 | Number | 206127550.6 | 216088798.8 | 204893479.0 | 217322870.4 | 203452497.2 | 218763852.1 | 201634643.3 | 220581706.0 | 196624460.6 | 225591888.8 |
| 209419699.6 | 7567760.2 | 2043 | Number | 204319029.3 | 214520370.0 | 203055213.3 | 215784186.0 | 201579500.1 | 217259899.2 | 199717831.1 | 219121568.2 | 194586889.7 | 224252509.6 |
| 207695550.5 | 7739500.6 | 2044 | Number | 202479127.1 | 212911973.9 | 201186630.5 | 214204470.5 | 199677427.9 | 215713673.1 | 197773510.8 | 217617590.2 | 192526129.4 | 222864971.6 |
| 205904115.3 | 7904146.9 | 2045 | Number | 200576720.3 | 211231510.4 | 199256727.8 | 212551502.9 | 197715419.1 | 214092811.6 | 195770999.0 | 216037231.7 | 190411987.3 | 221396243.3 |
| 204024455.9 | 8061036.7 | 2046 | Number | 198591317.2 | 209457594.6 | 197245124.0 | 210803787.8 | 195673221.9 | 212375689.9 | 193690206.9 | 214358705.0 | 188224824.0 | 219824087.9 |
| 202070757.8 | 8211279.0 | 2047 | Number | 196536355.7 | 207605159.8 | 195165072.1 | 208976443.4 | 193563872.7 | 210577642.8 | 191543898.1 | 212597617.5 | 185976650.9 | 218164864.7 |
| 200077073.2 | 8354891.8 | 2048 | Number | 194445876.2 | 205708270.3 | 193050609.2 | 207103537.2 | 191421405.3 | 208732741.1 | 189366102.0 | 210788044.4 | 183701485.4 | 216452661.1 |
| 198045417.7 | 8491777.6 | 2049 | Number | 192321959.6 | 203768875.8 | 190903832.8 | 205187002.7 | 189247936.2 | 206842899.3 | 187158958.9 | 208931876.6 | 181401533.7 | 214689301.8 |
| 195943338.3 | 8620898.8 | 2050 | Number | 190132852.5 | 201753824.1 | 188693162.4 | 203193514.2 | 187012087.1 | 204874589.4 | 184891346.0 | 206995330.5 | 179046376.7 | 212840299.9 |


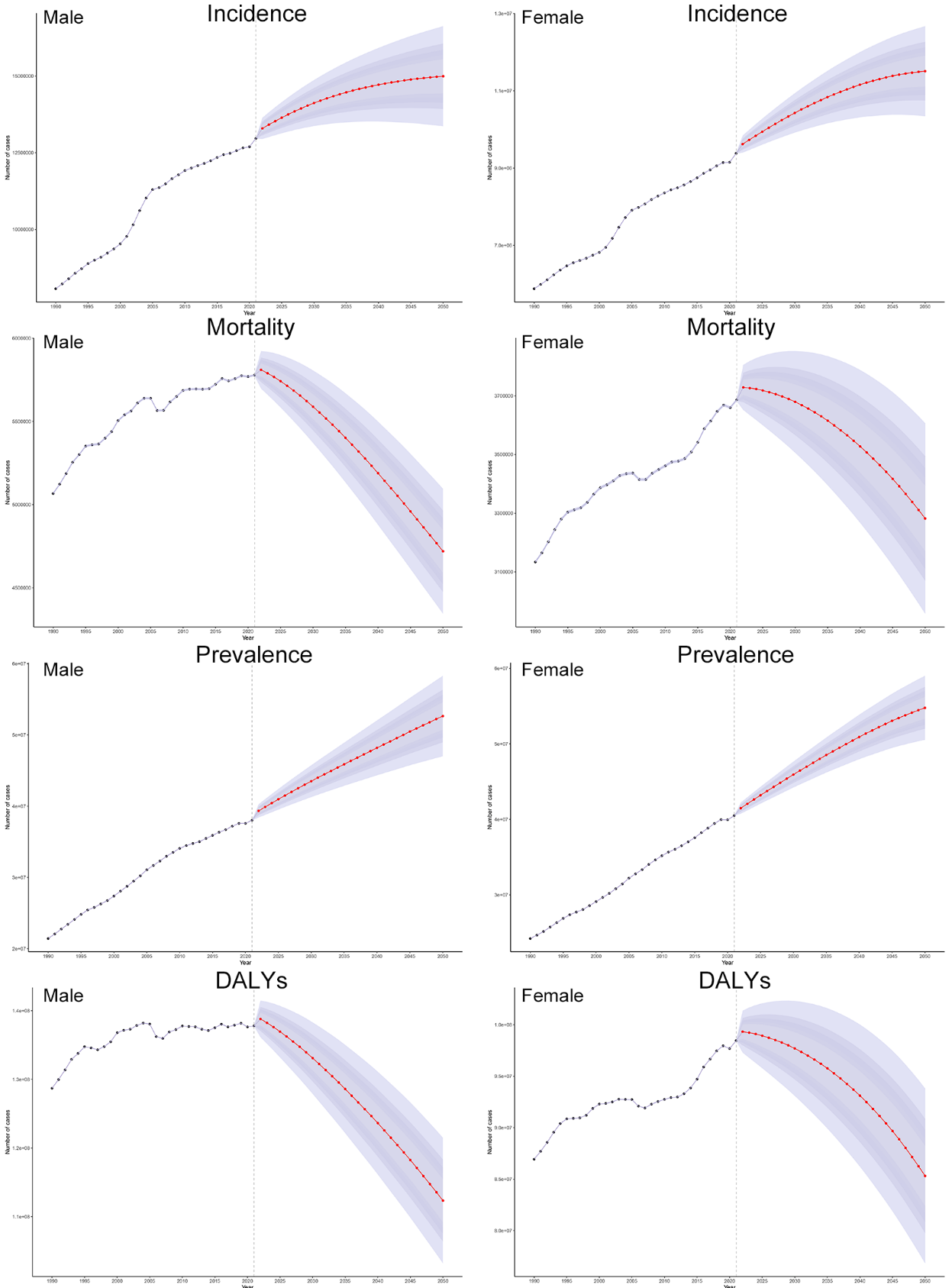


**Figure S15.** The historical trends and future projections of global cancer from 1990 to 2050: incidence, mortality, prevalence, and DALYs, by sex


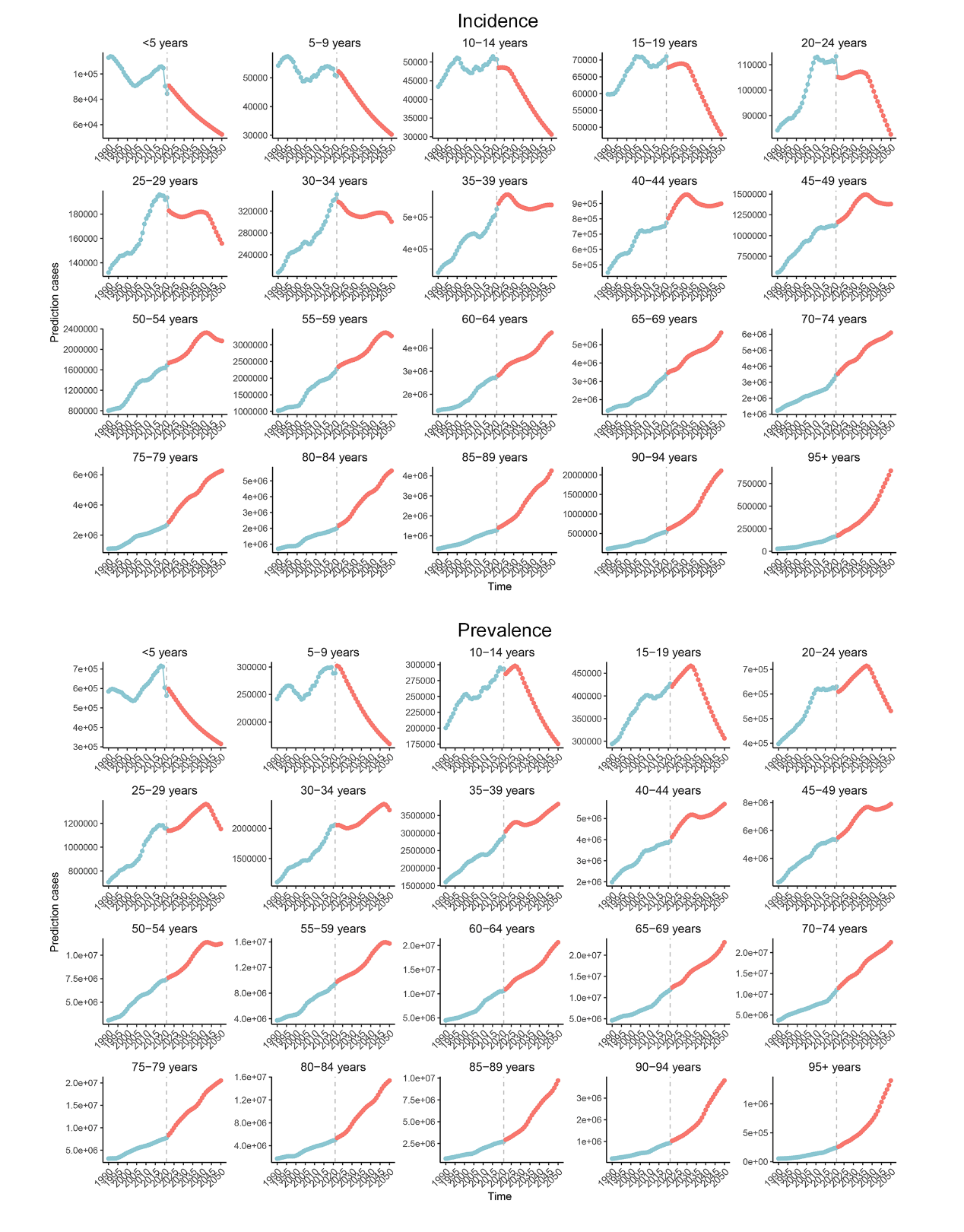


**Figure S16.** The historical trends and future projections of global cancer incidence and prevalence from 1990 to 2050, by age group


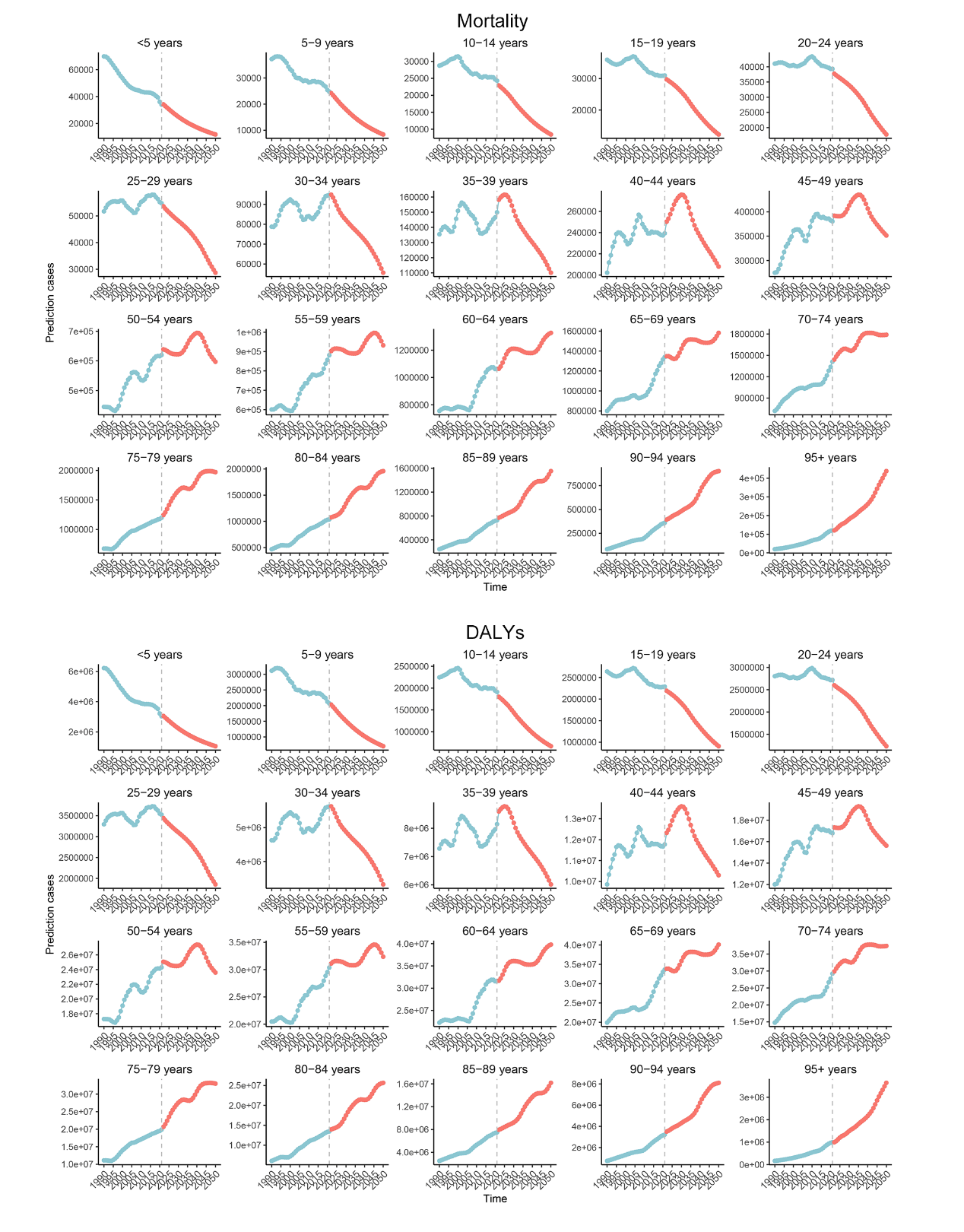


**Figure S17.** The historical trends and future projections of global cancer mortality and disability-adjusted life years (DALYs) from 1990 to 2050, by age group


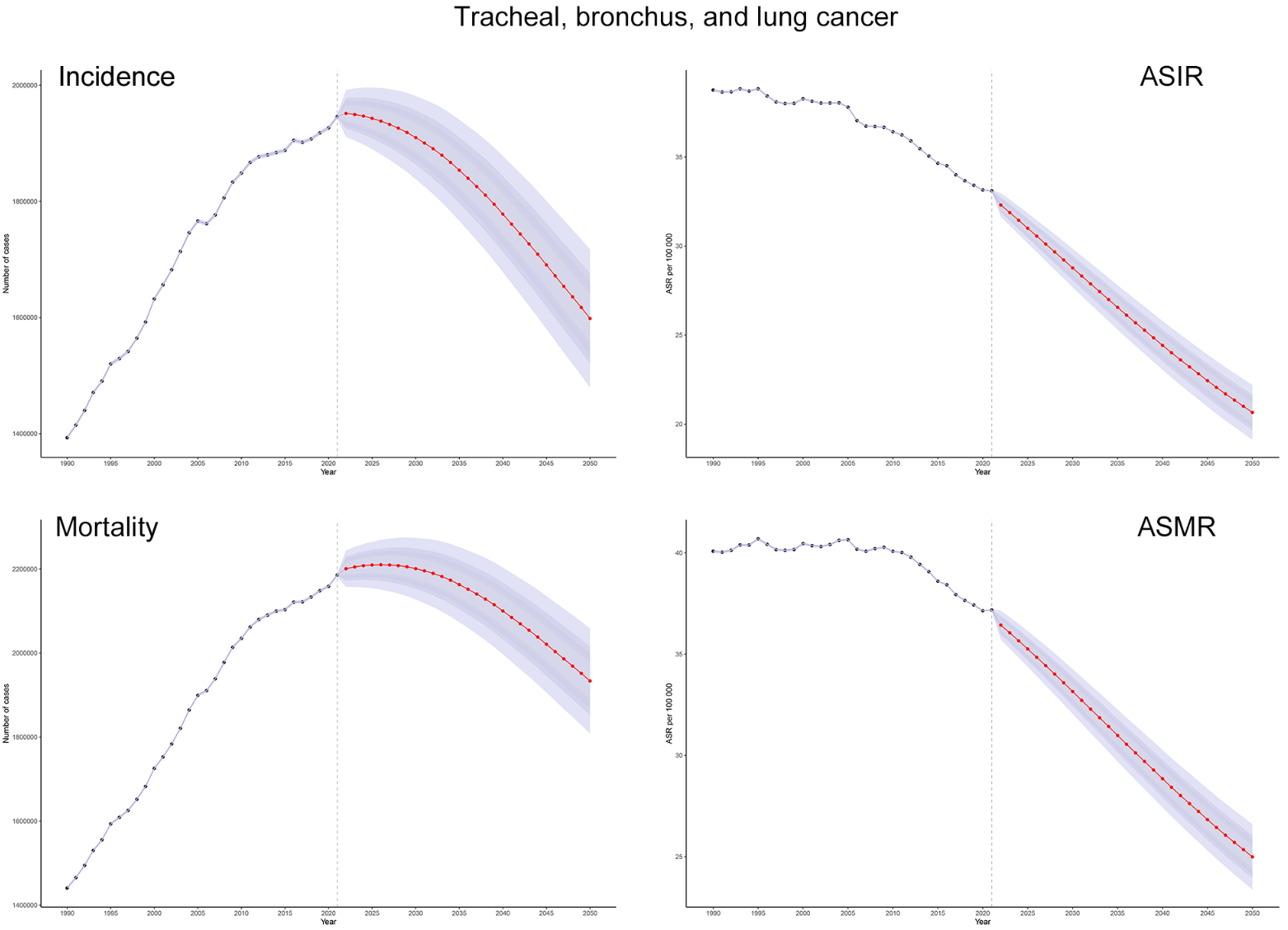


**Figure S18.** Projected burden of tracheal, bronchus, and lung cancer


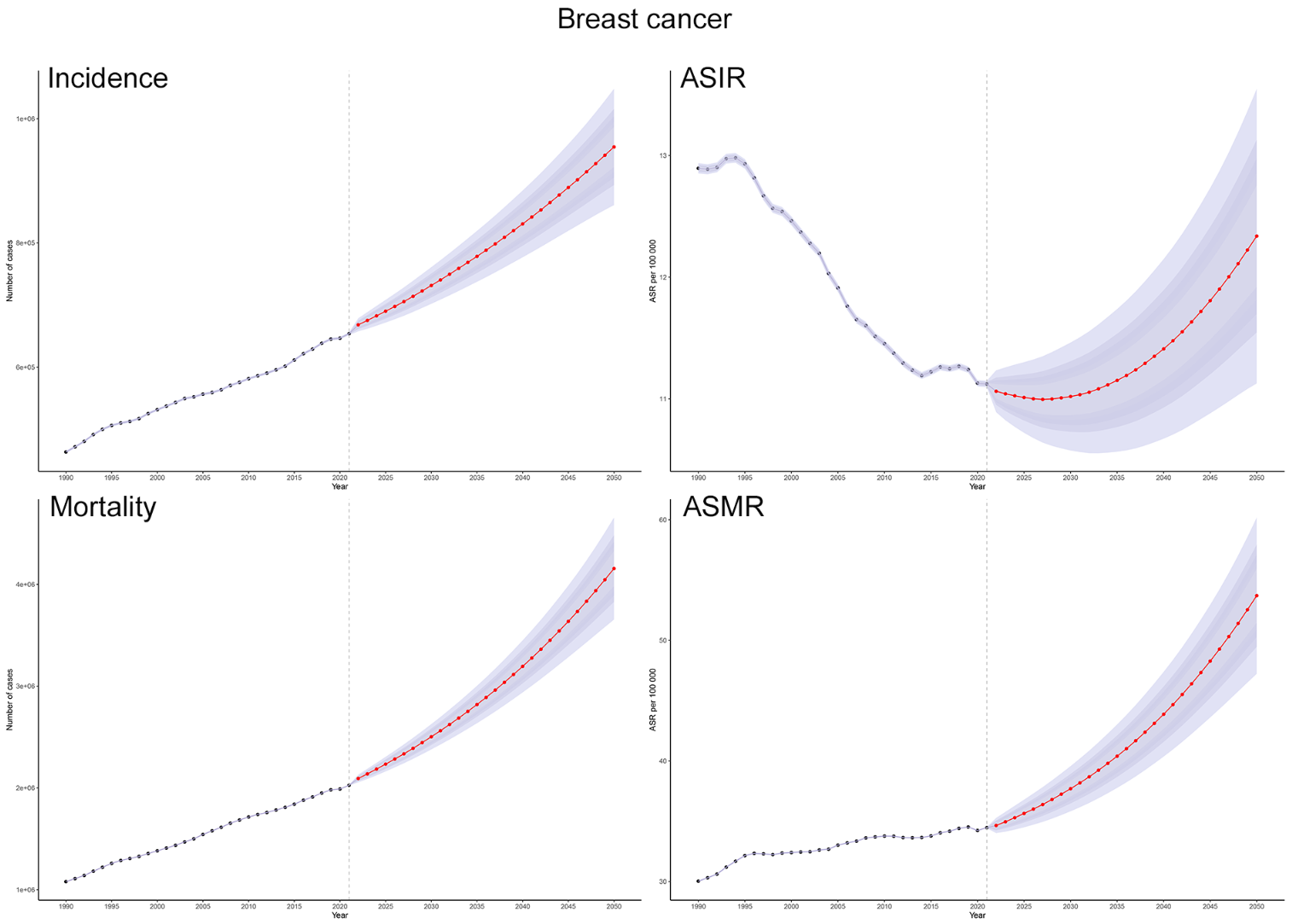


**Figure S19.** Projected burden of breast cancer


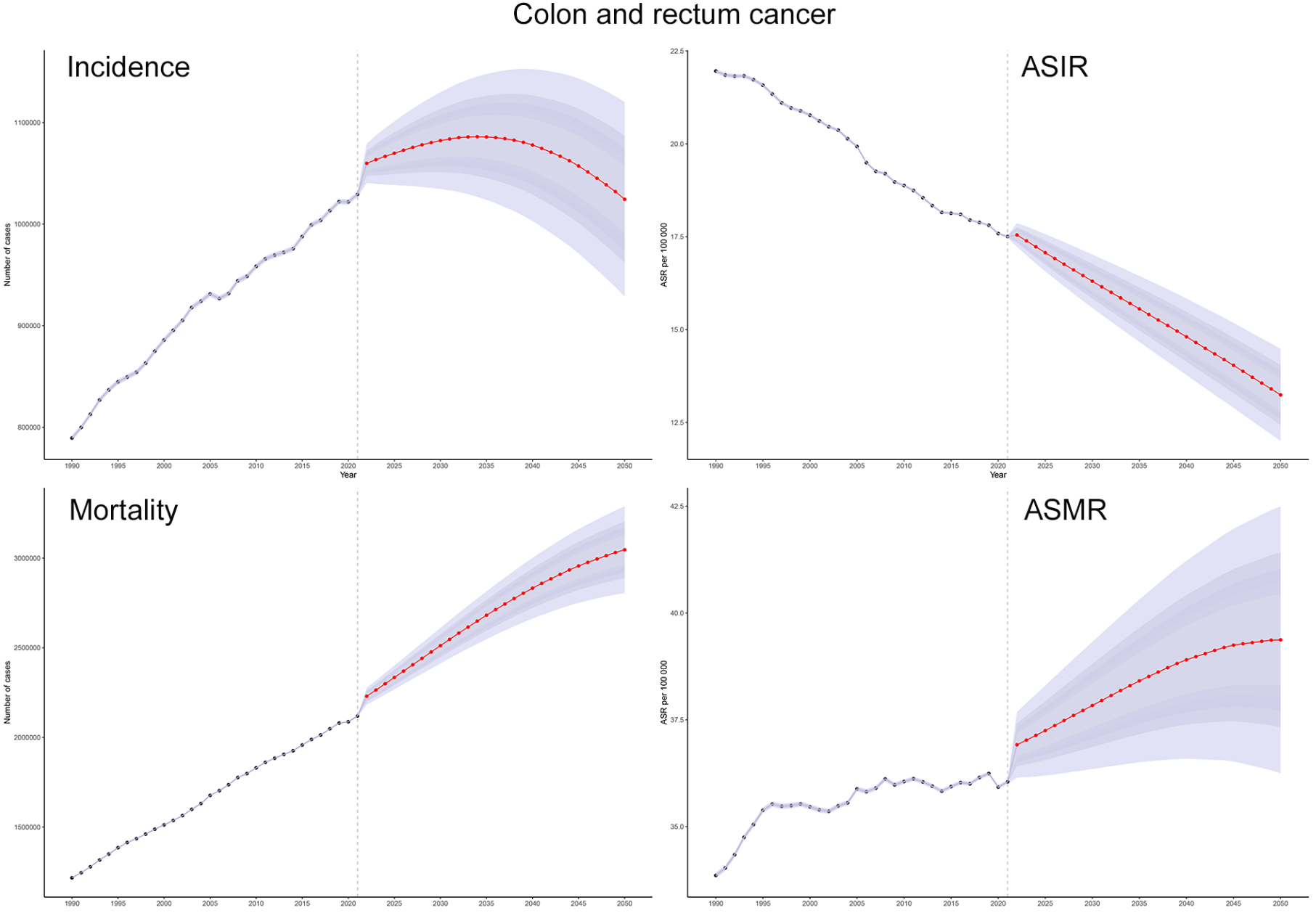


**Figure S20.** Projected burden of colon and rectum cancer


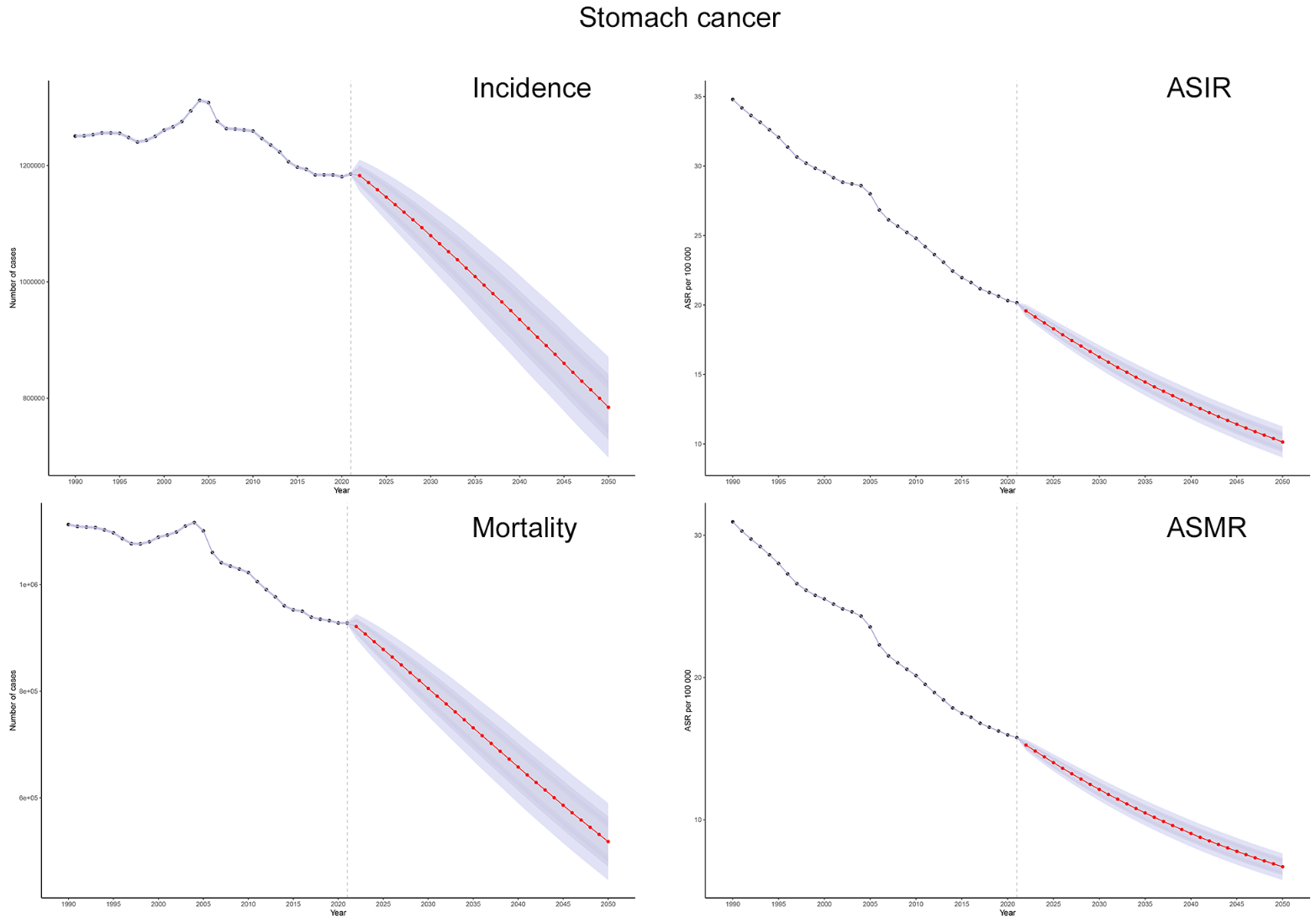


**Figure S21.** Projected burden of stomach cancer


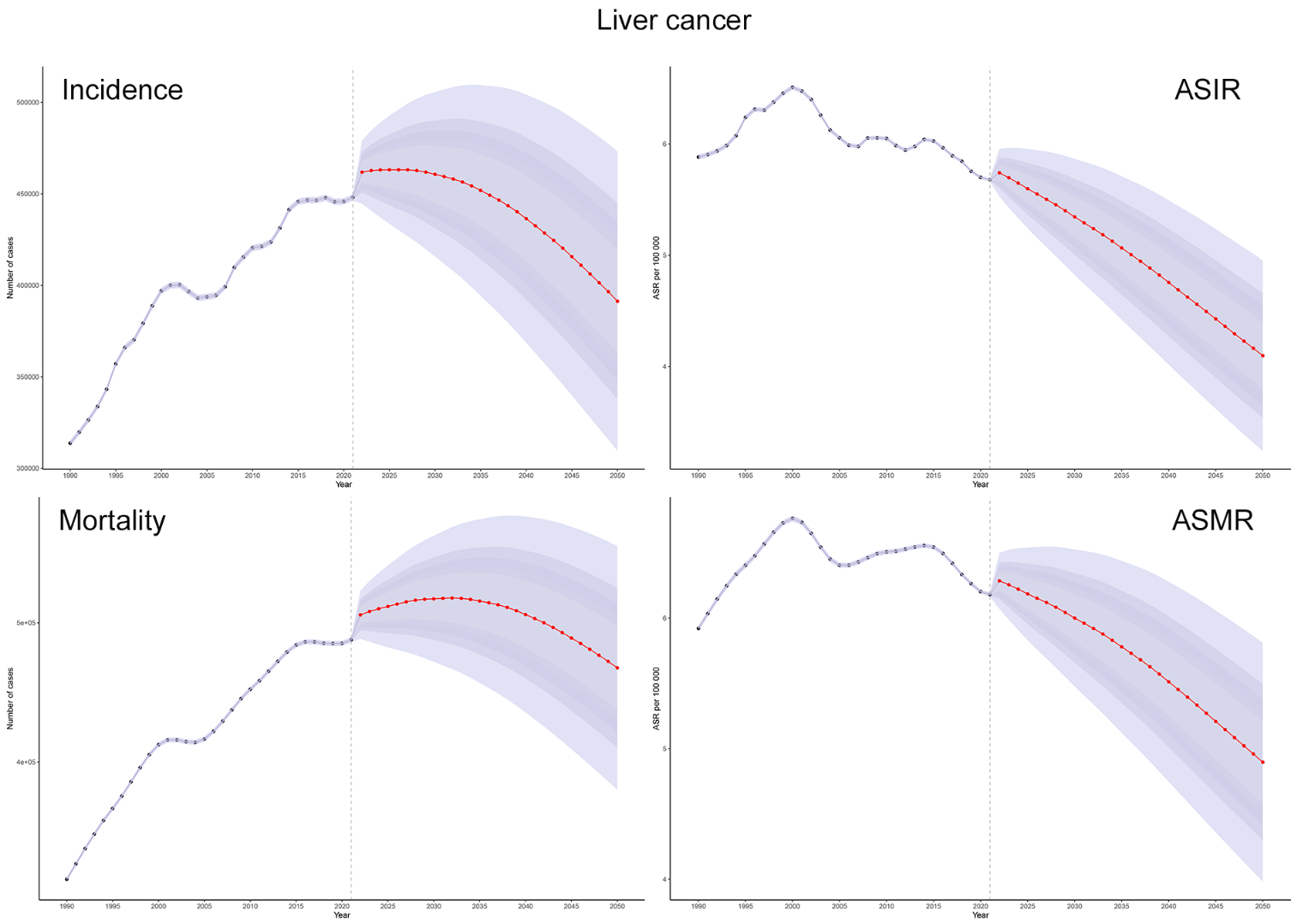


**Figure S22.** Projected burden of liver cancer


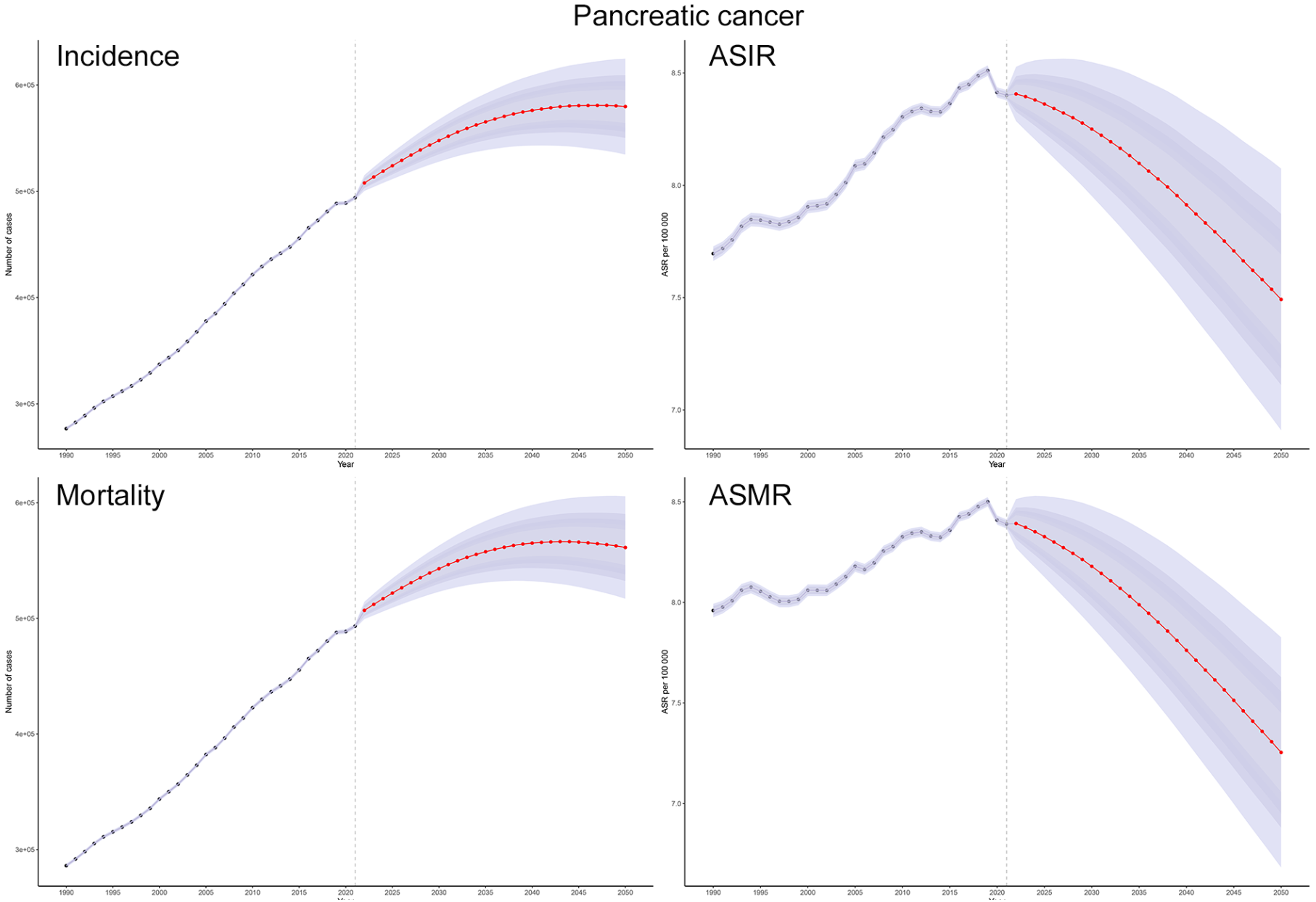


**Figure S23.** Projected burden of pancreatic cancer


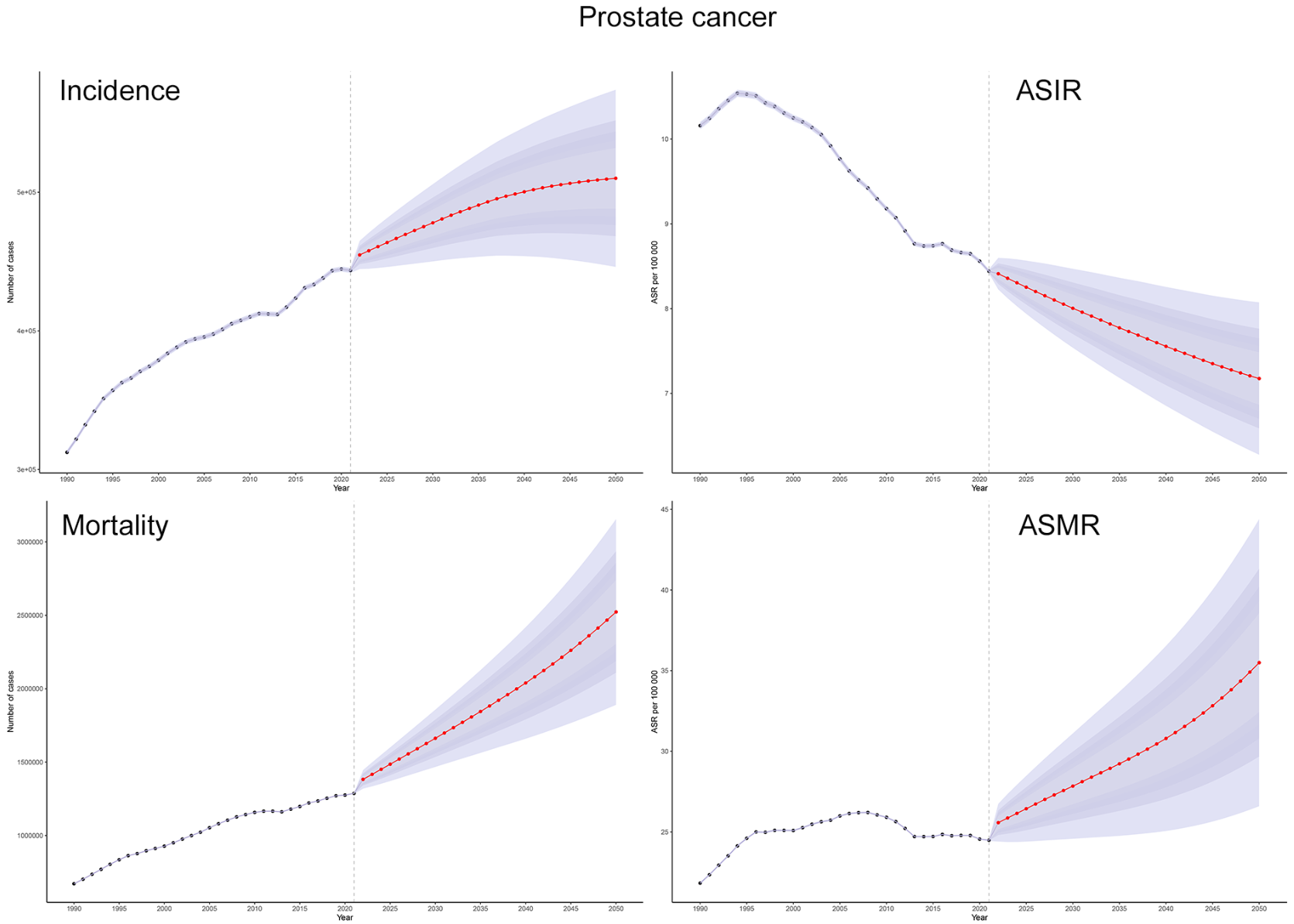


**Figure S24.** Projected burden of prostate cancer


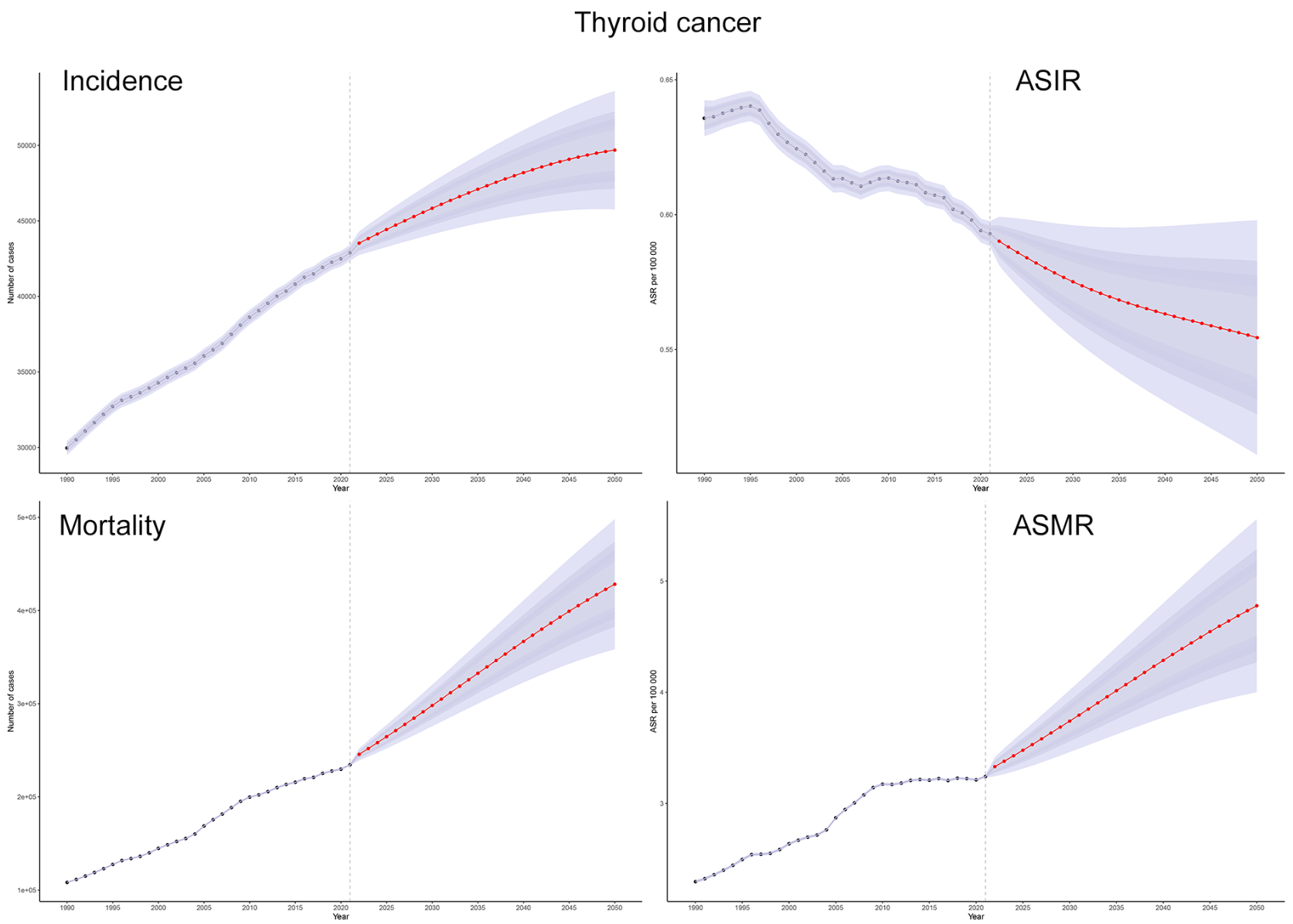


**Figure S25.** Projected burden of thyroid cancer
